# Wigwams: identifying gene modules co-regulated across multiple biological conditions

**DOI:** 10.1093/bioinformatics/btt728

**Published:** 2013-12-18

**Authors:** Krzysztof Polanski, Johanna Rhodes, Claire Hill, Peijun Zhang, Dafyd J. Jenkins, Steven J. Kiddle, Aleksey Jironkin, Jim Beynon, Vicky Buchanan-Wollaston, Sascha Ott, Katherine J. Denby

**Affiliations:** ^1^Warwick Systems Biology Centre and ^2^School of Life Sciences, University of Warwick, CV4 7AL, UK

## Abstract

**Motivation:** Identification of modules of co-regulated genes is a crucial first step towards dissecting the regulatory circuitry underlying biological processes. Co-regulated genes are likely to reveal themselves by showing tight co-expression, e.g. high correlation of expression profiles across multiple time series datasets. However, numbers of up- or downregulated genes are often large, making it difficult to discriminate between dependent co-expression resulting from co-regulation and independent co-expression. Furthermore, modules of co-regulated genes may only show tight co-expression across a subset of the time series, i.e. show condition-dependent regulation.

**Results:** Wigwams is a simple and efficient method to identify gene modules showing evidence for co-regulation in multiple time series of gene expression data. Wigwams analyzes similarities of gene expression patterns within each time series (condition) and directly tests the dependence or independence of these across different conditions. The expression pattern of each gene in each subset of conditions is tested statistically as a potential signature of a condition-dependent regulatory mechanism regulating multiple genes. Wigwams does not require particular time points and can process datasets that are on different time scales. Differential expression relative to control conditions can be taken into account. The output is succinct and non-redundant, enabling gene network reconstruction to be focused on those gene modules and combinations of conditions that show evidence for shared regulatory mechanisms. Wigwams was run using six *Arabidopsis* time series expression datasets, producing a set of biologically significant modules spanning different combinations of conditions.

**Availability and implementation:** A Matlab implementation of Wigwams, complete with graphical user interfaces and documentation, is available at: warwick.ac.uk/wigwams.

**Contact:** k.j.denby@warwick.ac.uk

**Supplementary Data:** Supplementary data are available at *Bioinformatics* online.

## 1 INTRODUCTION

Elucidating the regulatory mechanisms mediating biological processes is a key challenge in many eukaryotic organisms. Much regulation occurs at the transcriptional level; however, despite our ability to profile genome-wide gene expression and the availability of bioinformatics tools to analyze sequence information, our understanding of gene regulatory networks underlying biological processes is still relatively basic. Regulatory interactions are often common, meaning that the ability to understand the regulation of a response requires a mathematical or computational network model. Underlying these network models is the knowledge of regulatory mechanisms. Techniques to identify regulatory mechanisms, such as genome-wide chromatin immunoprecipitation sequencing (Robertson *et al.*, 2007) and matrix-based yeast one-hybrid (Y1H) (Deplancke *et al.*, 2006), have improved, but these techniques are not high-throughput. Therefore, it is crucial to be able to make high-quality predictions of regulatory mechanisms using existing data; these predictions can then be tested in focused experimental and modelling efforts.

Time series experiments are often used to examine the dynamics of gene expression (Belling *et al.*, 2013; Windram *et al.*, 2012), and with the decreasing cost of profiling techniques, more datasets covering multiple time series showing how an organism responds to different conditions are becoming available. Such data offer the opportunity to study shared regulatory mechanisms that are used to regulate genes under more than one condition. Such shared regulatory mechanisms may drive gene expression in the same way in each time series, or they may be modified to drive expression on a different time scale or to change the direction of regulation (activation versus repression). To facilitate gene network reconstruction, it is important to develop tools that can map out which gene modules are co-regulated in what combinations of conditions.

It is a long-standing assumption that co-expression may reflect co-regulation (Altman and Raychaudhuri, 2001), and using data from multiple conditions can improve the correlation between the two (Yeung *et al.*, 2004). However, in noisy biological systems, co-regulated genes may still show some differences in their expression, and there may be more than one regulatory mechanism that can drive genes with a particular expression pattern. Following a perturbation, such as infection or treatment with a chemical stimulus, many genes change in expression and even with high-resolution time series, large numbers of genes can show a similar expression profile (Weinstock-Guttman *et al.*, 2003; Windram *et al.*, 2012). As a result, it can be challenging to distinguish between *dependent co-expression* indicative of co-regulation and *independent co-expression* of genes that achieve a similar expression pattern in different ways. It is important to note that both dependent and independent co-expression may pass statistical tests that are geared towards testing the similarity of expression patterns and/or the tightness of a gene cluster relative to other clusters. Therefore, tools that solely aim to detect clusters of similarly expressed genes may not discriminate informative dependent co-expression from uninformative independent co-expression.

Multiple high-resolution time series of gene expression for a single organism under different conditions provide a powerful approach for identifying dependent co-expression of genes likely to be controlled by a common upstream regulator. However, while an increasing number of time series would help to improve the specificity of co-expression (i.e. co-expression across more time series is more likely to be dependent co-expression), it is unlikely that a single group of genes will be co-expressed across all the datasets. For example, it is known that there is significant cross-talk between signalling networks for different plant hormones in *Arabidopsis*, but not all of the components are playing a role in the response to every hormone (Robert-Seilaniantz *et al.*, 2011). Furthermore, some of the detected co-expression across multiple datasets may still be independent co-expression due to the abundance of particular expression profiles rather than due to a shared regulatory mechanism. Hence, there is a need for an algorithm that can identify modules of genes dependently co-expressed across subsets of time series data, combining the increased specificity of multiple time series datasets with biological reality.

A myriad of clustering algorithms have been developed to assign genes into clusters based on the similarity of their expression profiles across a single time series or multiple static (i.e. single or few time points) datasets (Madeira *et al.*, 2010; Maere *et al.*, 2008; Meng *et al.*, 2009; Reiss *et al.*, 2006). A few algorithms have also been developed to specifically cluster genes using two or more time series datasets, such as BHC (Savage *et al.*, 2009) and SplineCluster (Heard *et al.*, 2005). However, these algorithms partition genes into clusters and do not enable identification of genes co-expressed across subsets of the data. A few methods are capable of identifying co-expression across subsets of the data, but these come with their own drawbacks. Tensor methods (Li *et al.*, 2012; Zhang *et al.*, 2012) require the timescale of the experiments to be uniform throughout. EDISA (Supper *et al.*, 2007) does not require the same timescale across all of the datasets, but it is non-deterministic. None of these methods is able to incorporate differential expression relative to control time series into the analysis, and crucially, none statistically evaluates dependent co-expression versus independent co-expression. ENIGMA (Maere *et al.*, 2008) can account for genes’ differential expression, but the method was designed for a series of static expression data. CCC-Biclustering (Madeira *et al.*, 2010) tests biclusters for statistical significance against a null hypothesis of independent expression profile evolution, but the method is only capable of analyzing a single time course experiment.

Wigwams (*Wigwams identifies genes working across multiple situations*) is a simple, deterministic and efficient method capable of identifying groups of dependently co-expressed genes, termed gene modules, spanning subsets of the available time series datasets. Wigwams is a comprehensive method; all potential dataset combinations are scanned for gene modules by rigorously testing putative gene modules around each gene differentially expressed in a dataset. Wigwams evaluates each putative module for statistical significance and provides a non-redundant output of gene modules showing significant co-expression across varying combinations of the time series data. Each gene may be assigned to one or more gene modules or to no module at all. Wigwams requires little user input (further aided by easy-to-use graphical user interfaces) and is computationally inexpensive and relatively fast, making it a useful method to analyze multiple time series experiments for evidence of co-regulation. We demonstrate that gene modules identified by Wigwams are often enriched for Gene Ontology (GO) terms (Ashburner *et al.*, 2000) and known transcription factor (TF) binding motifs indicating biological relevance. We also provide experimental evidence of potential co-regulation. Wigwams is a powerful tool to utilize the resolution of time series expression data in a statistically rigorous approach for identification of co-regulated gene modules. It can make a direct contribution to gene regulatory network analysis and computational prediction of regulatory mechanisms.

## 2 MATERIALS AND METHODS

Here we outline the Wigwams algorithm indicating the various steps and decisions to be taken in applying this method to multiple time series expression datasets. A Matlab implementation is provided at warwick.ac.uk/wigwams along with relevant documentation.

### 2.1 Input

The input to Wigwams is a matrix of gene expression values for all the time series data to be analyzed with unique gene identifiers and annotation of each time series sample. Two additional matrices can be provided to improve the biological relevance and ease of interpretation of resulting modules: one indicating which genes are differentially expressed (DE) in each time series dataset (previously determined relative to a control time series) in a binary manner (0 for non-DE, 1 for DE), and the second providing annotation information for each unique gene identifier. If data on DE genes are not provided, then all genes are treated equally. A graphical user interface has been created to aid in the construction of data formats for use in Wigwams based on raw input files. A second graphical user interface facilitates running the individual steps of the Wigwams method described in Sections 2.2, 2.3 and 2.4 below.

The expression profiles are standardized on a per-gene basis in each dataset, and a matrix containing the expression profiles of all genes differentially expressed in at least one of the conditions is created. The expression profiles of non-DE genes within each condition are randomly shuffled across non-DE genes. This prevents non-DE genes from contributing to gene modules, as it unlinks dependencies of expression profiles across conditions for those genes (see Section 2.2 below). Any non-DE gene making a (coincidental) contribution to a gene module is removed from the module (see below). Therefore, although the randomization step eases the data processing, it has no effect on the eventual output of Wigwams, leaving the Wigwams output deterministic.

### 2.2 Identifying modules spanning multiple datasets

**Fig. 1.**
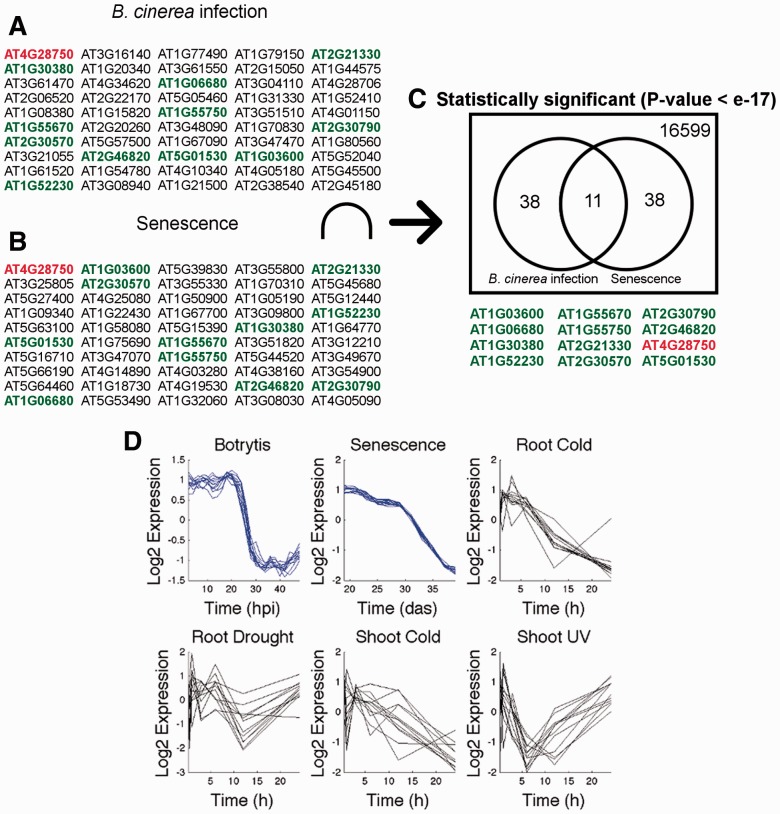
Strong evidence for dependent co-expression is detected during the module identification stage. (**A**) and (**B**) are two lists of 50 genes that are most correlated to the seed gene’s expression profile in *B.cinerea* infection and senescence, respectively (seed gene shown in red). (**C**) The overlap between the two lists (genes shown in green) is determined, and the expression profiles of the identified genes are shown in (**D**). To evaluate whether the observed overlap is likely to have occurred by chance a hypergeometric test is performed (yielding a *P*-value below 1 e-17 in this example). Overlaps deemed statistically significant are likely to discriminate dependently co-expressed genes from independently co-expressed genes. Such overlaps are therefore termed ‘modules’. In this example, the module is ‘spanning’ *B.cinerea* infection and senescence. Hpi, hours post inoculation; das, days after sowing, h, hours

This stage of Wigwams is outlined in Supplementary Figure S1, with an example shown in Figure 1. The aim of these steps is to detect all evidence of co-regulation (in the form of dependent co-expression) across the multiple time series datasets, regardless of the redundancy of resulting modules. Each gene that is DE in two or more conditions is deemed a ‘seed’ gene and is tested sequentially. For each condition in which the seed gene is DE, the other genes in the expression matrix are ranked on the basis of how well their expression profile in that time series is correlated with the expression profile of the seed gene. Genes are ranked with the most correlated gene at the top of the list. In the work presented in this article, the Pearson correlation coefficient was used as the similarity measure. Alternative metrics could be substituted without a need to change the Wigwams method itself.

For each combination of conditions in which the seed gene is DE, the size of the overlap between the top-ranked co-expressed genes in each dataset is tested statistically (Fig. 1 shows an example). This evaluates whether the similarities of gene expression observed within each time series are dependent across datasets. A significant *P*-value suggests that a regulatory mechanism is at work that (i) targets a significantly similar set of genes in each condition considered and (ii) induces expression profile similarity in each condition. However, no restriction is made regarding the similarity of gene expression profiles across different conditions. Therefore, a regulatory mechanism that targets a similar set of genes under different conditions, but with a different effect on expression (e.g. activating in one condition but repressing in another) can still be detected by this statistical test.

All possible combinations of conditions are tested (i.e. sets of two or more time series up to the number of independent time series used), using the top *n* most correlated genes in each dataset, where the user specifies a range of values for *n*. In our work, we have used *n* = 50, 100, 150, 200 and 250 to be able to detect regulatory mechanisms targeting <10 to >100 genes. Wigwams processes the detection of modules (described in this Section) independently for each *n* and pools the results.

To determine whether the observed overlap is statistically significant, the hypergeometric test is used. For the purposes of the Matlab implementation of Wigwams, the hypergeometric test function by Meng *et al.* (2009) was used. This was modified to enable the significance of overlaps between more than two time series to be assessed. Given a universe of size 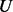 and two sets of size 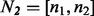, the probability of observing an overlap of at least size 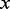 by chance equals

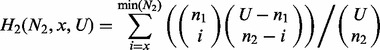

One can expand this for 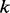 sets of size 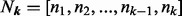 by assuming the probability of observing an overlap of at least size 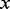 between the 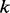 sets to be equal to the sum of the products of the probability of observing an overlap of exactly size 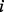 between 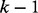 sets of size 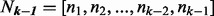 and the probability of observing an overlap at least of size 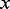 between two sets of size 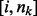, for 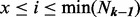. Owing to the nature of the hypergeometric test, the probability of observing an overlap of exactly size 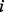 between 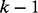 sets is equal to the difference of the probability of observing an overlap at least of size 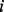 and the probability of observing an overlap of at least size 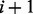 for 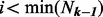, and to 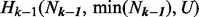 for 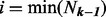. Combining that into a formula yields

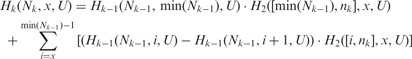



This modification makes it possible to evaluate the statistical significance of overlaps between three and more time series datasets.

The Bonferroni correction (Bland and Altman, 1995) is applied to the *P*-values from the hypergeometric test. Given a desired significance threshold α (0.05 was used for this study), the Bonferroni correction proposes an adjusted α

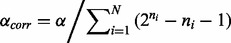

where 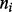 is the number of datasets in which gene 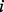 is differentially expressed. 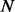 is the number of genes. Overlaps with a *P*-value below the adjusted significance threshold were deemed to have statistically significant dependent co-expression. Such overlap gene lists are considered gene modules and always include the seed gene by design. If any non-DE genes are included in these overlaps, these are removed from the putative gene modules before evaluating the statistical significance. Therefore, the output is a list of gene modules showing statistically significant dependent co-expression across two or more time series datasets. However, at this stage multiple modules may contain similar genes as if seed genes have similar expression profiles, similar gene modules will be created around these.

### 2.3 Merging similar modules spanning the same time series subset

**Fig. 2.**
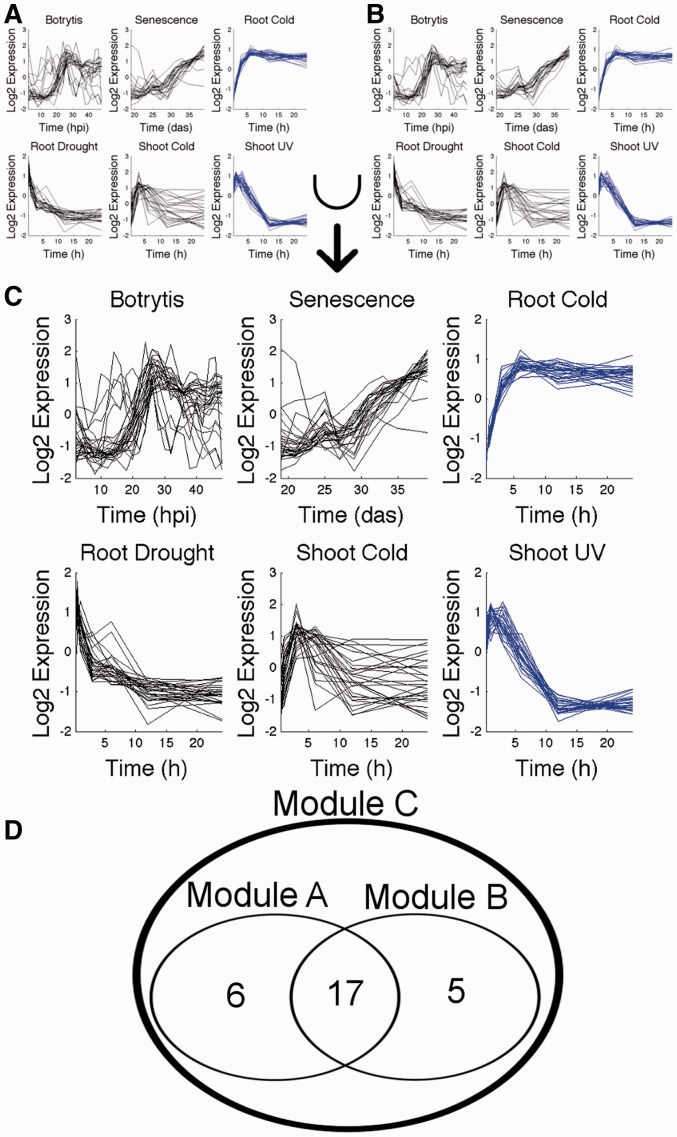
Merging. The modules shown in (**A** and **B**) span the same combination of conditions (depicted in blue; other time series shown in black), and feature a sizeable overlap in gene membership. Merging joins the two modules into a combined module shown in (**C**). The mean expression profiles of the larger module A are used to determine whether the five genes unique to module B are expressed with sufficient similarity to be included in the joint module, preserving the extra information that is contributed by module B. In this example, all genes of module B are accepted. Hpi, hours post-inoculation; das, days after sowing, h, hours

This process in Wigwams is outlined in Supplementary Figure S2, with an example shown in Figure 2. It is important that the output of Wigwams is in a useful format for biologists to use, and hence at this stage of the algorithm, gene modules with a sizeable overlap of gene membership are merged. However, this stage only reduces redundancy among modules that are spanning the same combination of conditions.

Owing to the way modules are formed in Wigwams, modules with large overlap will also have similar expression profiles. In this study, modules with an overlap >30% of the smaller module’s gene membership are merged. This simplifies the output because one module, containing genes co-expressed with similar profiles, is formed rather than two. The choice of overlap threshold was based on the distribution of overlap size (as a proportion of the smaller module) as seen in Supplementary Figure S1. The distribution of overlap size can be plotted within Wigwams providing a tool for the user to set this threshold. Genes from the smaller module are added to the larger module provided that their expression profile is sufficiently correlated with the mean expression profile of the larger module. We used a correlation threshold of 0.8, which needed to be fulfilled for each of the datasets the modules span. In addition, two modules whose mean expression profiles across relevant time series datasets are highly correlated (Pearson correlation coefficient of at least 0.9 for each of the datasets) are also merged, regardless of the overlap in gene membership. The merging stage produces a set of modules with greatly reduced redundancy but without loss of essential information (see Table 1). Broader regulatory phenomena are reconstructed using previously identified statistically significant modules. The user can adjust the thresholds to shift the trade-off between the ability to see subtle differences between similar modules and the ability to get a succinct overview of key signals in the data.

**Table 1.** Gene module information during Wigwams analysis

Wigwams stage	Raw	After merging	After sweeping	After thresholding
Modules	4434	161	128	78
Overlaps	38 964	4	3	3
Max overlap	50	19	19	19
Two conditions	3465	44	39	39
Three conditions	787	70	50	26
Four conditions	173	40	33	12
Five conditions	8	6	5	1
Six conditions	1	1	1	0
Mean module size	22	56	63	100
Total size	97 313	9030	8050	7827
Unique genes	4444	4239	4197	4194

*Note*: The table shows the number of modules, number of pairs of modules that span the same condition combination with at least 10 genes in common (overlaps), maximum number of genes shared by a pair of modules spanning the same conditions (maximum overlap), number of modules spanning two to six conditions, mean module size, total size of the identified modules and number of unique genes they feature (Unique genes) for the initial module list (raw) and at different stages of Wigwams analysis.

### 2.4 Sweeping redundant modules spanning different dataset subsets

**Fig. 3.**
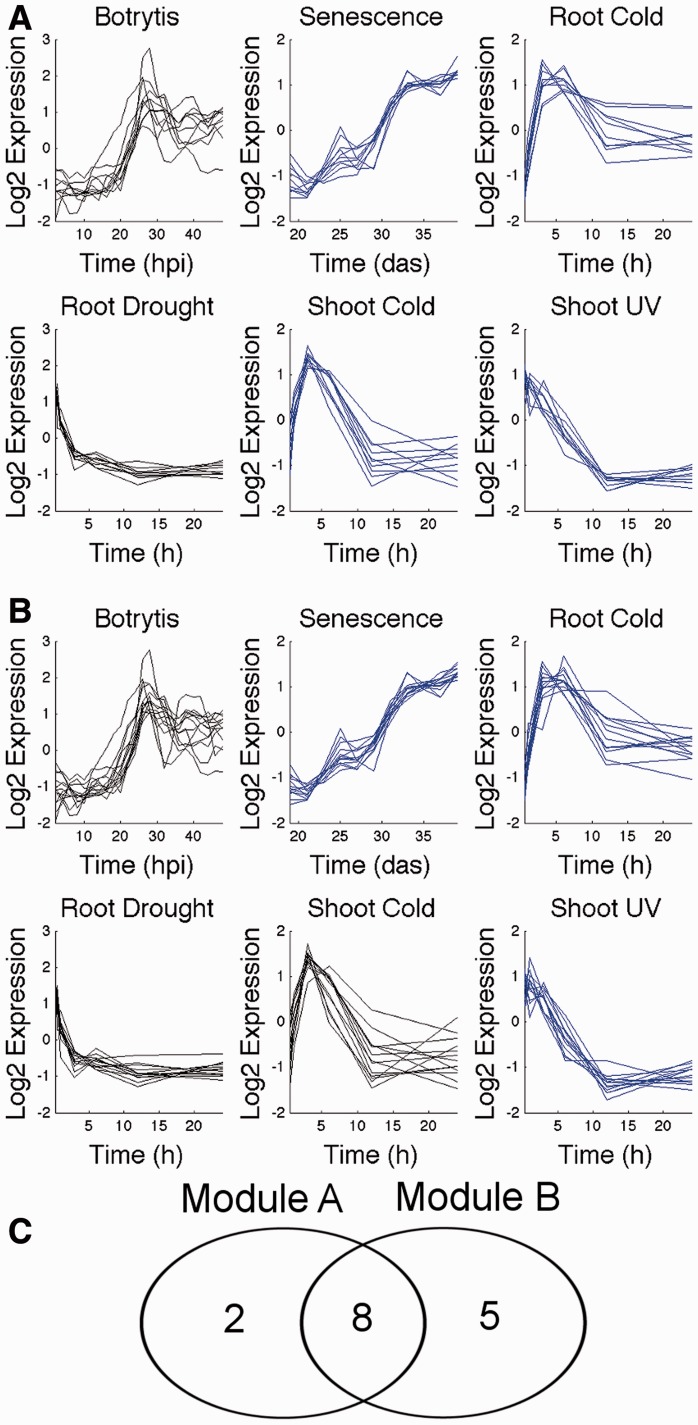
Sweeping. Sweeping evaluates those pairs of modules where one spans only a subset of conditions compared with the other. The module spanning fewer conditions is removed if it does not contribute enough new information. In this example, module A spans four conditions (senescence, root response to cold, shoot response to cold, shoot response to UV, shown in blue), while module B only spans three of these and contains only five genes that are not already members of module A. Module B is discarded. hpi, hours post-inoculation; das, days after sowing, h, hours

This stage of Wigwams is outlined in Supplementary Figure S3, with an example shown in Figure 3. Sweeping addresses a second kind of redundancy. For example, in the case of a module containing genes significantly co-expressed across three conditions, significant dependent co-expression is likely to be picked up for each pair of these time series as well, yielding another three modules. The gene membership of the module spanning more conditions is compared with that of modules spanning subsets of these time series. If the overlap is comparable with the size of the module spanning fewer conditions, then this module is discarded on the basis of it not contributing significant new information. In this study, the module spanning fewer conditions was discarded if the overlap featured at least 50% of its gene members.

The output at this final stage of Wigwams is a list of modules generated from genes showing statistically significant dependent co-expression, processed to optimize the number of different expression patterns contained in these modules and reduce redundancy between module gene membership.

### 2.5 GO term and TF binding motif enrichment testing

GO term (Ashburner *et al.*, 2000) enrichment was tested with the Cytoscape plugin BiNGO (Maere *et al.*, 2005) using the GO_Full ontology with the hypergeometric test and the Benjamini–Hochberg correction to control the false discovery rate (Benjamini and Hochberg, 1995). The whole genome *Arabidopsis* annotation was used as a reference set. Analysis of overrepresented TF binding motifs in module promoter sequences was carried out exactly as in Breeze *et al.* (2011), using information from the PLACE (Higo *et al.*, 1999) and TRANSFAC (Matys *et al.*, 2006) databases. *P*-values were adjusted using the Benjamini–Hochberg correction. For each gene, 500 bp of DNA upstream of the transcriptional start site was tested. As a control for GO term and TF binding motif analysis, 78 groups of genes were randomly generated from the 16 686 genes forming the Wigwams input. These 78 random modules were the same size as the 78 final modules identified by Wigwams.

### 2.6 Yeast one-hybrid technique

The yeast one-hybrid TF library screen was performed as described in Hickman *et al.* (2013) using three overlapping promoter fragments of ∼400 bp spanning ∼1 kb upstream of the transcription start site of each gene.

## 3 RESULTS

We applied Wigwams to analyze a set of six time series datasets of gene expression responses to environmental stress in the model plant *Arabidopsis thaliana*. Two of the datasets were high-resolution time series, one obtained from leaves following infection with the fungus *Botrytis cinerea* (Windram *et al.*, 2012), the other from leaves developing from maturity to senescence (ageing) (Breeze *et al.*, 2011). The other four datasets had fewer time points, and captured responses to abiotic stresses [shoot and roots after cold stress, roots during drought and shoots after ultraviolet (UV) light exposure]. These were obtained from AtGenExpress (Kilian *et al.*, 2007). The two groups of experiments were performed with different microarray platforms (Redman *et al.*, 2004; Sclep *et al.*, 2007), and the datasets were found to have 19 886 genes in common.

For the *B.cinerea* infection and senescence time series, the curated lists of DE genes were used (Breeze *et al.*, 2011; Windram *et al.*, 2012). For the AtGenExpress datasets, differential gene expression was determined using the GPTwoSample test (Stegle *et al.*, 2010), with a score threshold of four. In all, 16 686 genes were DE over time in at least one condition (Supplementary Dataset S1), with 12 447 genes DE in at least two conditions and hence eligible for inclusion into Wigwams modules.

### 3.1 Wigwams systematically scans the data for evidence of co-regulation

The module identification procedure uses one gene at a time (‘seed gene’) and each combination of conditions in turn and tests whether the expression pattern of the seed gene across these time series may be driven by a regulatory mechanism acting on a number of genes under more than one condition. This is illustrated in Figure 1 for the case of a set of two conditions. For each time series, gene expression similarity to the seed gene is evaluated and the list of genes that are most strongly correlated with the seed gene assembled. Genes in each list show co-expression (across multiple conditions), which could be dependent co-expression driven by a common mechanism, or independent co-expression where multiple mechanisms induce similar expression patterns. If the expression similarity observed in each time series is the result of a common regulatory mechanism, then it is likely that this mechanism will target a similar set of genes in each condition. Wigwams tests this hypothesis. In the example of Figure 1, of 50 genes in each list, 11 genes (plus the seed gene) are in common between the two lists. By the hypergeometric test, the likelihood of making this observation by chance is below 1e-17. This provides strong evidence that the co-expression observed is not independent co-expression, but dependent co-expression driven by a shared regulatory mechanism. Hence, the 11 genes in the overlap (plus the seed gene) are likely to be under a common regulatory influence and are considered a module.

The module identification procedure was run for all DE genes and all dataset combinations. This stage of the analysis took 2 h 53 min on a Dell Precision M4700 computer (2.8 GHz Intel Core i7-3840QM processor, 16 GB DDR3 SDRAM at 1600 MHz, 64-bit Windows 7 Professional, Matlab R2012b), producing a list of 4434 statistically significant gene modules likely to be showing dependent co-expression spanning two to six conditions (Table 1). Of the 12 447 DE genes in two or more conditions, 4444 were placed in at least one module.

### 3.2 Wigwams effectively removes redundancy among modules

As Wigwams considers every DE gene as a seed gene during the module identification stage, the method is comprehensive, but the output after the first stage is likely to have a high degree of redundancy. The merging algorithm merges modules with similar gene membership and/or highly similar expression profiles (exemplified in Fig. 2). The sweeping algorithm removes modules that have a large overlap with another module, but only show dependent co-expression across a smaller subset of conditions (exemplified in Fig. 3). In both cases, the essential information characterizing the expression phenomenon observed is maintained, while redundant information is removed.

After the merging stage, the initial 4434 modules were condensed into 161 modules (Table 1), while the number of unique genes assigned to at least one module only decreased from 4444 to 4239. The genes lost during merging had expression profiles not sufficiently similar to the mean expression profile of the larger module to be included. The average size of modules increased from 22 to 56 genes, while overlaps among modules were strongly reduced. Redundancy within the remaining 161 modules was further reduced by the sweeping stage. This reduced the list to 128 modules with an average size of 63, while the number of unique genes included in modules decreased only slightly from 4239 to 4197.

We decided to exclude small modules from further analyses, as (i) we wanted to get an overview of expression signatures driven by major regulatory mechanisms and (ii) although the excluded modules did pass rigorous statistical testing, the evidence base for these modules is not as wide as for the larger ones. We required a minimum of 10 genes for modules spanning two or three conditions, a minimum of 8 if spanning four, and a minimum of 5 genes if spanning five or six time series. These thresholds were simply chosen on the basis that fewer genes will be co-expressed across a larger number of datasets and small modules will provide little functional information. Our thresholding resulted in a final list featuring 78 modules spanning two to five conditions and covering 4194 unique genes (Table 1 and Supplementary Dataset S2).The mean module size is 100 genes.

### 3.3 Wigwams reveals expression signatures of regulatory mechanisms

**Fig. 4.**
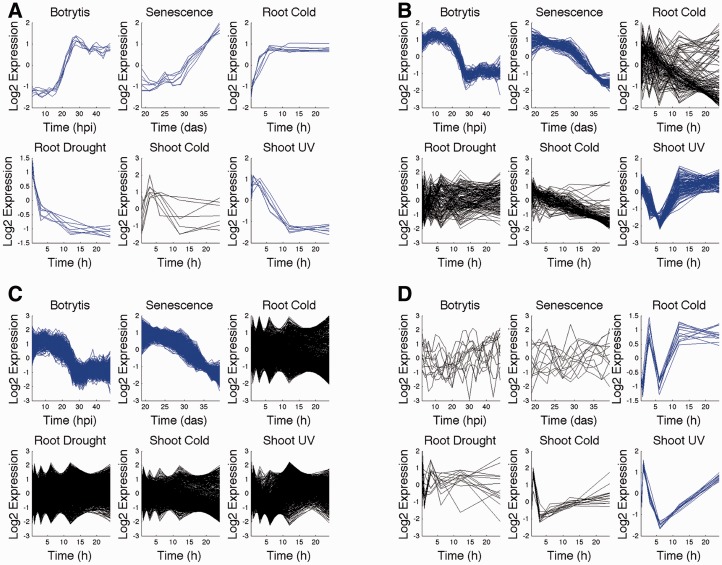
Four examples of modules showing different regulatory phenomena detected by Wigwams. Each module is represented by the gene expression profiles of its members across the six conditions. Shown in blue are conditions for which there is evidence for dependent co-expression. (**A**) is the smallest module, which has seven genes and appears to be dependently co-expressed in every condition except for shoot response to cold. The genes are activated in three conditions and repressed in two. (**B**) is a 131-gene module spanning *B.cinerea* infection, senescence and shoot response to UV. (**C**) is the largest module with 1238 genes, including all 131 genes from module (B), but only spans two conditions. (**D**) features 13 genes with unusual expression profiles in root response to cold and shoot response to UV. hpi, hours post inoculation; das, days after sowing; h, hours

Four modules from the set of 78 are shown in Figure 4 (expression profiles for all modules are in Supplementary Dataset S3). Strong evidence for dependent co-expression has been detected for time series coloured in blue. The evidence for co-regulation of genes in these modules does not merely stem from the tightness of expression patterns, but is further supported by the dependence of expression similarities across time series. Although some expression profiles appear correlated in conditions not part of the module (e.g. shoot cold in Fig. 4D), we have not found evidence for dependence of co-expression in these time series. Expression similarity arises from a large number of genes sharing a similar profile in that condition.

Interestingly, module A shows regulation in different directions depending on conditions. During *B.cinerea* infection, senescence and root response to cold genes in the module are upregulated, while they are downregulated during root response to drought and shoot response to UV, consistent with the idea that the mechanism regulating these genes operates in a different mode under different conditions.

**Fig. 5.**
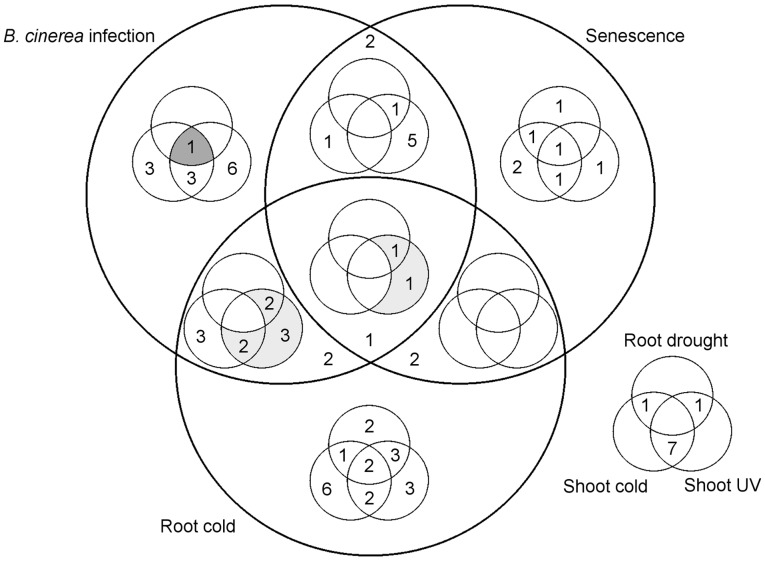
The number of modules identified for each combination of conditions. Three conditions are represented by large circles; the other three by small circles. Evidence for dependent co-expression was found across a range of combinations of conditions, ranging from two time series up to five time series. By analyzing the number of modules detected for different combinations of conditions, network reconstruction efforts can be focused on time series combinations showing evidence for shared regulatory mechanisms. The nine modules featuring *B.cinerea* infection, root response to cold and shoot response to UV light are shaded light grey. The single module spanning *B.cinerea* infection, root response to drought and shoot response to cold is shaded dark grey

The full set of 78 modules contains modules dependently co-expressed across different combinations of conditions (Fig. 5 and Table 1). The modules detected by Wigwams can be considered the result of regulatory networks active during different stress responses. By analyzing the distribution and function of modules across different combinations of conditions, hypotheses can be made about the function and complexity of the networks underlying these, and network reconstruction attempts can be directed towards suitable time series combinations. For example, a researcher interested in unravelling shared regulatory networks between the biotic stress of *B.cinerea* infection and abiotic stress can see from Figure 5 that only one module spans *B.cinerea* infection, root response to drought and shoot response to cold. As such, attempts to reconstruct a common regulatory network spanning these three stress responses do not appear to be well supported by the available data, as we see little evidence for a complex shared network. In contrast, there are nine modules spanning *B.cinerea* infection, root response to cold and shoot response to UV light, making this combination of stresses much more promising for elucidating a common regulatory network. Wigwams allows the researcher to rigorously examine available data for evidence of a regulatory network before embarking on modelling or experimental efforts.

### 3.4 Biological validation of detected modules

**Fig. 6.**
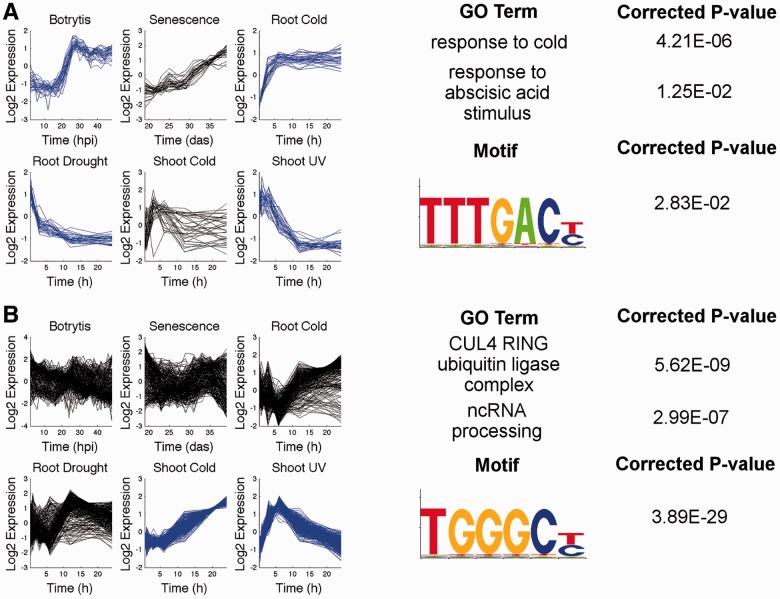
Wigwams modules are enriched in GO-terms and TF binding motifs. Shown above is an excerpt of the biological information obtained for two modules, showing dependent co-expression (in blue) and overrepresentation of GO terms and TF motifs in the promoters for genes in each module. (**A**) A 29-gene module spanning four conditions suggests a role for abscisic acid in linking the transcriptional responses to these four conditions. (**B**) A 269-gene module spanning shoot response to cold and UV light shows highly significant overrepresentation for the TCP binding motif, suggesting this motif may be underlying the dependent co-expression driving ubiquitination and non-coding RNA processing. hpi, hours post-inoculation; das, days after sowing, h, hours

The enrichment of genes involved in the same biological process is often used as an indication of co-regulation of a gene module. Therefore, we tested Wigwams modules using BiNGO (Maere *et al.*, 2005) and found that 71 of the 78 gene modules were overrepresented for GO terms (Ashburner *et al.*, 2000) relating to biological processes vis-à-vis 24 of the 78 random modules. This further supports the case for co-regulation of genes in each Wigwams module. Two examples of such modules, along with the identified overrepresented GO terms, are shown in Figure 6 (overrepresented GO terms for all modules are given in Supplementary Dataset S4).

The module shown in Figure 6A features 29 genes spanning four datasets and is enriched in GO terms ‘response to abscisic acid (ABA)’ and ‘response to cold’. This suggests a wider role of genes responding to cold and a role of ABA, a plant hormone, in mediating the link between the four conditions. The module shown in Figure 6B contains 269 genes dependently co-expressed across shoot response to cold and UV light, and is enriched in GO terms corresponding to the CUL4 RING ubiquitin ligase complex and non-coding RNA processing. The enrichment of genes with a shared function (members of the same complex) suggests that a specific mechanism acts to co-ordinate expression of the genes in this module.

Transcriptional gene regulation occurs by the binding of TFs to specific DNA sequences in promoters of genes. The same or similar TF binding motifs are often present in the promoters of co-regulated genes. We tested the Wigwams modules for enrichment of known TF binding motifs and found that 51 of the 78 modules had at least one overrepresented motif (Supplementary Dataset S5), suggesting that the module members were co-regulated. In comparison, 6 of the 78 random modules were overrepresented for a motif. The promoters of genes in the module shown in Figure 6A were overrepresented for the W box (de Pater *et al.*, 1996), suggesting that the dependent co-expression is driven by binding of TFs from the WRKY TF family (Eulgem *et al.*, 2000), known to play a key role in regulating plant stress responses (Chen *et al.*, 2012; Eulgem and Somssich, 2007). The module shown in Figure 6B was strongly enriched in a motif bound by the TCP TF family (Cubas *et al.*, 1999; Welchen and Gonzalez, 2006), again suggesting a mechanism for shared regulation.

Finally, we tested the validity of the Wigwams modules using the Y1H technique to test for direct binding of the same TF(s) to multiple genes within a module. As we are interested in gene regulatory networks, we targeted TF gene promoters from Wigwams modules. The promoters of two genes (At3g15210 and At5g05410) from a 26-gene module spanning *B.cinerea* infection and root and shoot responses to cold (Supplementary Fig. S4a) were screened against a TF library to identify TFs able to bind these DNA sequences. Both of these promoters were bound by TCP3 (At1g53230) and TCP1 (At1g67260), two members of the TCP TF family (Martín-Trillo and Cubas, 2010). In a 38-gene module spanning senescence and the root response to drought (Supplementary Fig. S4b), the promoters of three genes (At1g19180, At1g80840 and At3g23250) were screened and were bound by WRKY41, a member of the WRKY TF family (Eulgem *et al.*, 2000). Direct binding of these TFs to multiple gene promoters from the same module is a strong indication that the Wigwams algorithm is detecting dependent co-expression reflecting co-regulation.

## 4 DISCUSSION

Wigwams is a simple deterministic method capable of identifying groups of genes exhibiting statistically significant dependent co-expression across subsets of time series datasets, and using that information to construct larger non-redundant modules capturing broader transcriptional phenomena. Its comprehensive nature minimizes the odds of missing evidence of co-regulation, and the redundancy removal procedures provide the researcher with a succinct biologically informative output. In some cases, when examining the expression plots of gene modules, the module appears to exhibit co-expression in conditions that are not deemed significant. This demonstrates the power of Wigwams to select modules with statistically significant dependent co-expression. In these non-significant conditions, the given expression profile may have been abundant and/or the module members are not DE in that time series (i.e. expression profile in the control samples was similar).

When comparing Wigwams with other methods capable of identifying groups of genes co-expressed across different subsets of time series data, its main advantages are flexibility, statistical significance testing and relevance of the provided output. Additionally, Wigwams is able to account for differential expression of genes in each of the time series, and ensure that gene profiles are only tested for statistically significant dependent co-expression in relevant conditions. The value of testing the statistical significance of detected co-expression can be seen when comparing Wigwams with the EDISA algorithm (Supper *et al.*, 2007). When run on a permuted dataset, EDISA identified several co-expressed gene modules, whereas Wigwams did not identify any. Furthermore, we have shown the value of the comprehensive nature of Wigwams; it is capable of detecting dependent co-expression that EDISA misses (see Supplementary Material for details on this analysis). To our knowledge, Wigwams is the only algorithm capable of mining multiple time series (on varying time scales) for dependent co-expression across subsets of the time series.

The modules produced by Wigwams were demonstrated to be biologically relevant due to enrichment of GO terms (Ashburner *et al.*, 2000) and known TF binding sites, suggesting shared function and regulation between module members. We also provide experimental evidence for co-regulation showing that in yeast, a set of similar TFs bind to the promoters of multiple genes from a single Wigwams module. Although Y1H does not indicate the conditions under which these TFs bind to the gene promoters, or whether they bind *in planta*, it does indicate the potential for co-regulation.

The Wigwams tool is easy to use, with intuitive graphical user interfaces, comprehensive documentation and output provided in a clear manner that can be readily analyzed by tools such as BiNGO (Maere *et al.*, 2005) and MEME-LaB (Brown *et al.*, 2013). The algorithm is flexible, and intuitive parameters can be used to tailor the output as desired. Additionally, the module lists are saved as Matlab cell structures, enabling access to intermediate stages of Wigwams analysis, e.g. to identify the most statistically significant original smaller gene modules.

A more computationally tractable version of the modified hypergeometric test could enable modification of the Wigwams method. To obtain the *P*-values for an overlap spanning *k* sets, all the *P*-values for 2,3, … ,*k-*1 sets need to be generated. If the tests were more efficient, the algorithm could be modified to use correlation thresholds instead of pre-defined set sizes when evaluating overlaps, and non-DE genes could be excluded from any analysis without large adverse effects on run time due to varying universe size between dataset combinations.

Owing to the time and cost of experimental approaches to genome-wide network elucidation, computational inference of regulatory networks from time series expression data is a useful approach. However, despite the multitude of inference methods available, these methods are still only capable of inferring ‘moderately large dynamic networks’ (Kim *et al.*, 2013). Wigwams provides output that can be used to extend network models built with a subset of genes (e.g. using TFs only). Integrating Wigwams modules with a transcriptional network model can also provide condition-dependent information, such as indicating network neighbourhoods active during particular conditions. Wigwams modules can be viewed as the footprint of flux through regulatory networks under different conditions, and examining the abundance and functionality of modules for various combinations of conditions can provide insight into the commonality between the responses to different conditions at a more nuanced level than simple differential expression. Identification of modules showing contradictory expression under different conditions (e.g. upregulated in one dataset and downregulated in another) also suggests points of cross-talk within the regulatory network.

*Funding*: K.P., S.J.K. and A.J. were funded for this work by the Engineering and Physical Sciences Research Council (EPSRC)/Biotechnology and Biological Sciences Research Council (BBSRC) funded Warwick Systems Biology Doctoral Training Centre; J.R. was funded by a BBSRC Systems Approaches to Biological Research studentship; C.H., D.J., P.Z., J.B., V.B-W., S.O. and K.J.D. are part of the BBRSC funded grant Plant Response to Environmental Stress *Arabidopsis* (BB/F005806/1). The TF library was a gift from Franziska Turck, Max Planck Institute, Cologne, Germany.

*Conflict of Interest*: none declared.

## Supplementary Material

Supplementary Data
