# Supplementary material for: Wigwams: identifying gene modules co-regulated across multiple biological conditions
Source: Bioinformatics. 2013 Dec 18;30(7):962–70. doi: 10.1093/bioinformatics/btt728 (PMC3967106; doi:10.1093/bioinformatics/btt728)
Supplement: Supplementary Data [file supp_30_7_962__index.html]

Wigwams: identifying gene modules co-regulated across multiple biological conditions — Wigwams: identifying gene modules co-regulated across multiple biological conditions — Wigwams: identifying gene modules co-regulated across multiple biological conditions — Supplementary Data 

# Wigwams: identifying gene modules co-regulated across multiple biological conditions

## Supplementary Data

files

**Files in this Data Supplement:**

- Supplementary Data - pdf file
- Supplementary Data - pdf file
- Supplementary Data - xlsx file
- Supplementary Data - xlsx file
- Supplementary Data - xlsx file
- Supplementary Data - xlsx file
