# Supplementary material for: Wigwams: identifying gene modules co-regulated across multiple biological conditions
Source: Bioinformatics. 2013 Dec 18;30(7):962–70. doi: 10.1093/bioinformatics/btt728 (PMC3967106; doi:10.1093/bioinformatics/btt728)
Supplement: Supplementary Data [file supp_btt728_Supplementary_Material.pdf]

Supplementary Material

Supplementary Figure 1:

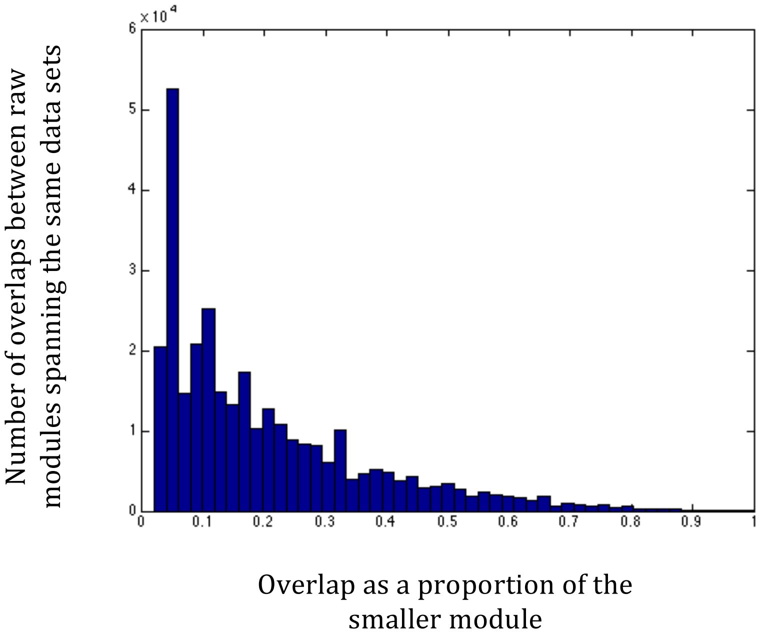

Supp. Figure 1: Histogram showing the size of the overlap between raw Wigwams modules spanning the same data set combinations (as a proportion of the smaller module) and the number of overlaps at each proportion. This allows the user to test for a suitable threshold to be used in merging modules. In our case, we selected a threshold of 0.3 as the number of overlaps greatly increased at lower proportions. The functionality to produce this histogram is included in the Wigwams software.

Supplementary Methods:

We compared the output of Wigwams with that of EDISA (Supper et al. 2007) as the only other algorithm capable of analyzing co-expression across subsets of multiple time series data sets across different time scales.

Firstly, we ran the whole 6 time series data set in EDISA. EDISA was only able to identify modules spanning two of the time series (Botrytis and senescence), none of the other data sets contributed to significant modules. Hence we generated a truncated data set of the 4 shorter time series, and the 2345 genes that are differentially expressed in at least three of these time series. Unlike Wigwams, EDISA does not take differential expression into account so this criterion should minimize the impact of differential expression on the results. Wigwams identified 37 final modules from this data set. EDISA identified 26 modules spanning 2 to 4 time series. The gene membership of modules spanning the same time series, or a subset of the same time series, was compared. Wigwams identified 11 modules that had no gene membership overlap to EDISA modules indicating that the comprehensive Wigwams approach is capable of identifying dependent co-expression that EDISA misses.

To assess the ability of Wigwams to detect dependent co-expression, we then

permuted the 2345 gene expression profiles across genes and independently within each time series, i.e. the expression profiles within each time series were randomly assigned to gene IDs. This permuted data was run through Wigwams and EDISA. There should be no evidence of co-regulation in the permuted data sets. As expected, Wigwams did not find any evidence of dependent co-expression (i.e. did not identify any significant modules). EDISA, however, identified 8 modules of co-expression (with a score threshold of 0.4). These modules reflect cases of independent co-expression. The difference in performance reflects the fact that EDISA is merely testing for tight co-expression whereas Wigwams is specifically testing for dependent co-expression.
