# Supplementary material for: Wigwams: identifying gene modules co-regulated across multiple biological conditions
Source: Bioinformatics. 2013 Dec 18;30(7):962–70. doi: 10.1093/bioinformatics/btt728 (PMC3967106; doi:10.1093/bioinformatics/btt728)
Supplement: Supplementary Data [file supp_btt728_Supp_Data_Set_3_expression_profiles.pdf]

Supplementary Data Set 3:

The expression profiles of genes in each of the final 78 modules identified by Wigwams. The time series data sets spanned by each module are highlighted in blue and included in the module identifier, with 1 to 6 corresponding to *B. cinerea* infection, senescence, root response to cold, root response to drought, shoot response to cold and shoot response to UV light respectively.

Module1 – 1,2,6

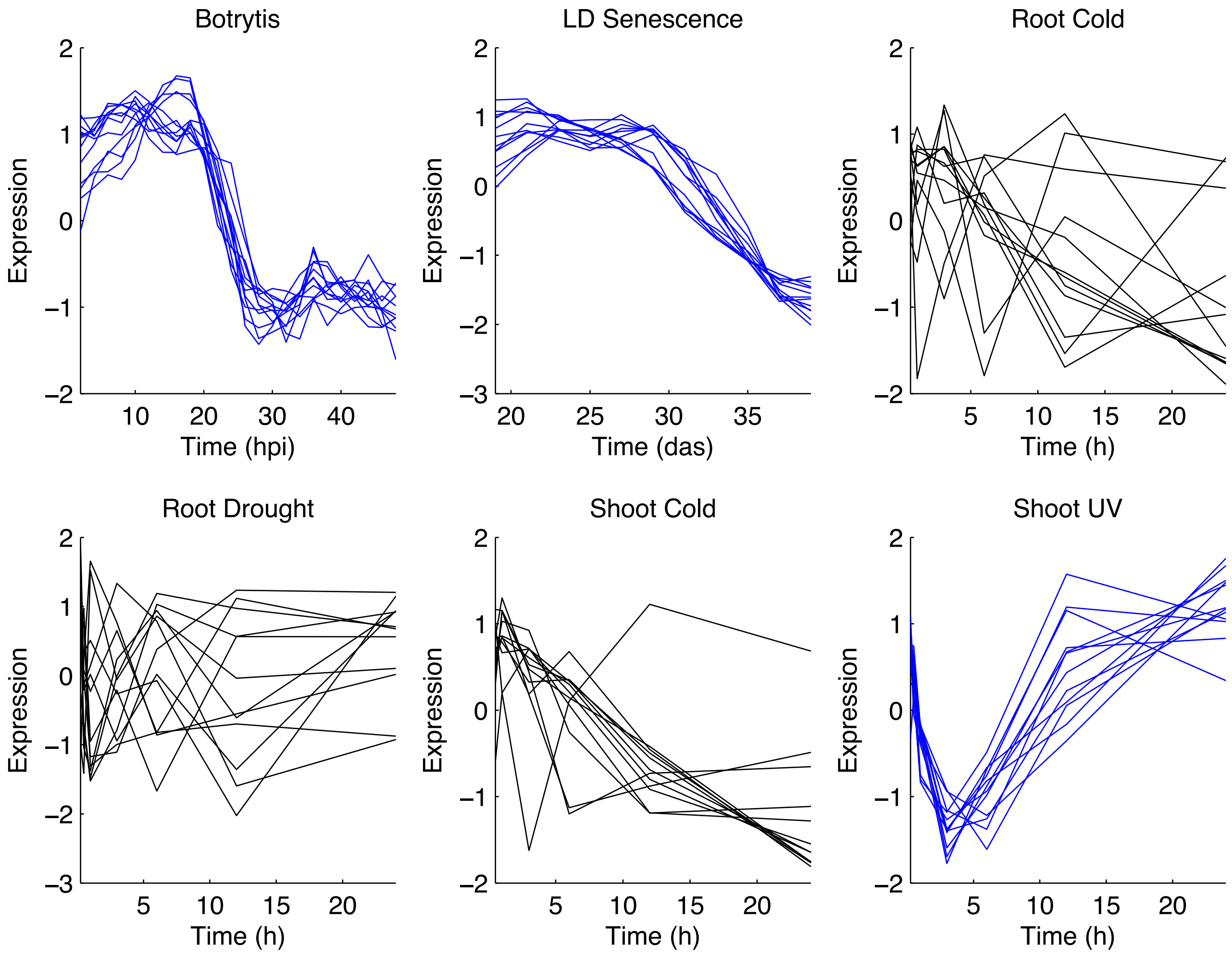
Botrytis
LD Senescence
Root Cold
Root Drought
Shoot Cold
Shoot UV
Expression
Time (hpi)
Time (das)
Time (h)
2
1
0
−1
−2
−3
10
20
30
40
25
35
5
15

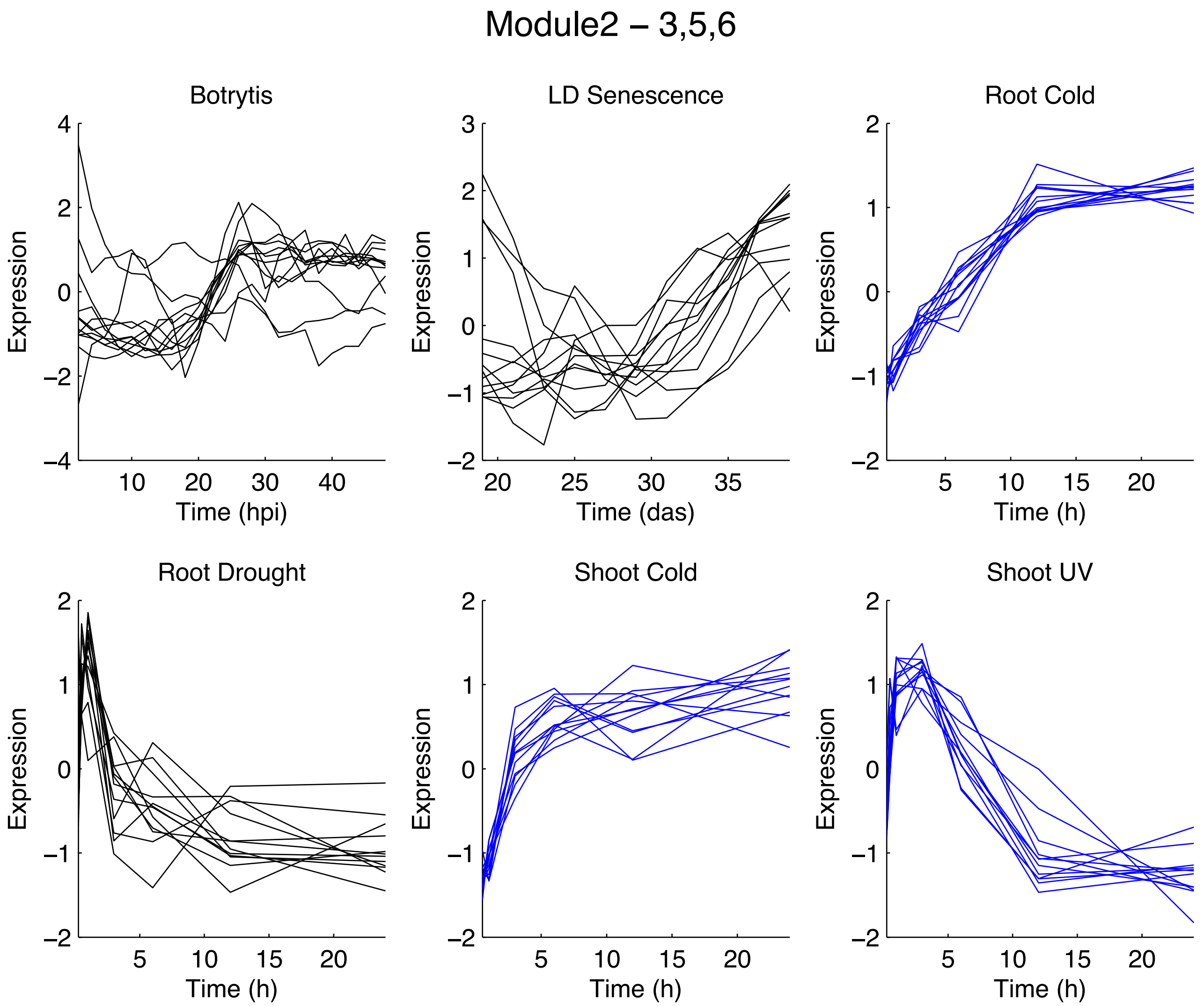
Module2 – 3,5,6
Botrytis
LD Senescence
Root Cold
Root Drought
Shoot Cold
Shoot UV
Expression
Time (hpi)
Time (das)
Time (h)

Module3 – 5,6

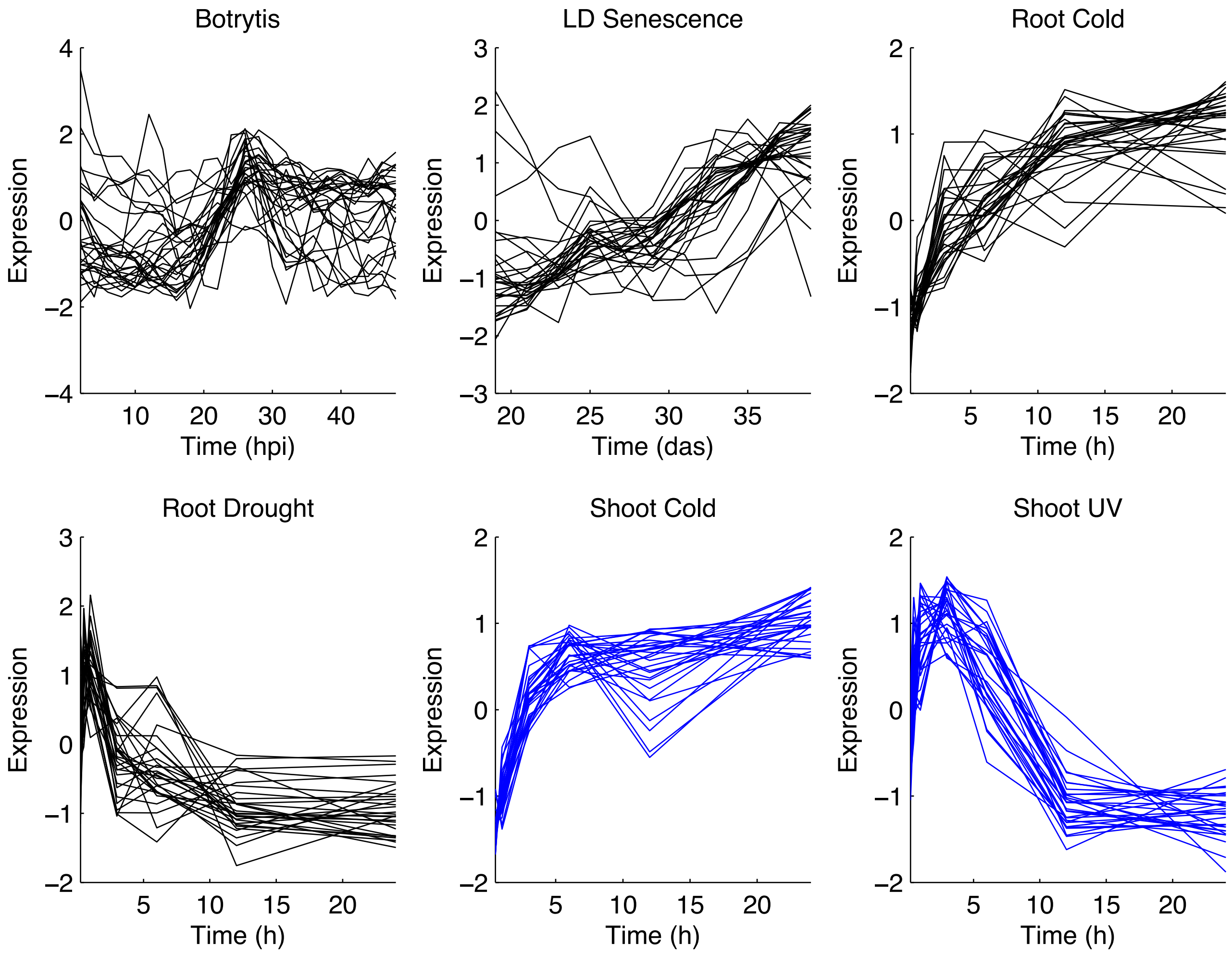
Botrytis
LD Senescence
Root Cold
Root Drought
Shoot Cold
Shoot UV
Expression
Time (hpi)
Time (das)
Time (h)
4
2
0
−2
−4
10
20
30
40
3
1
−1
−3
25
35
5
15

Module4 – 3,6

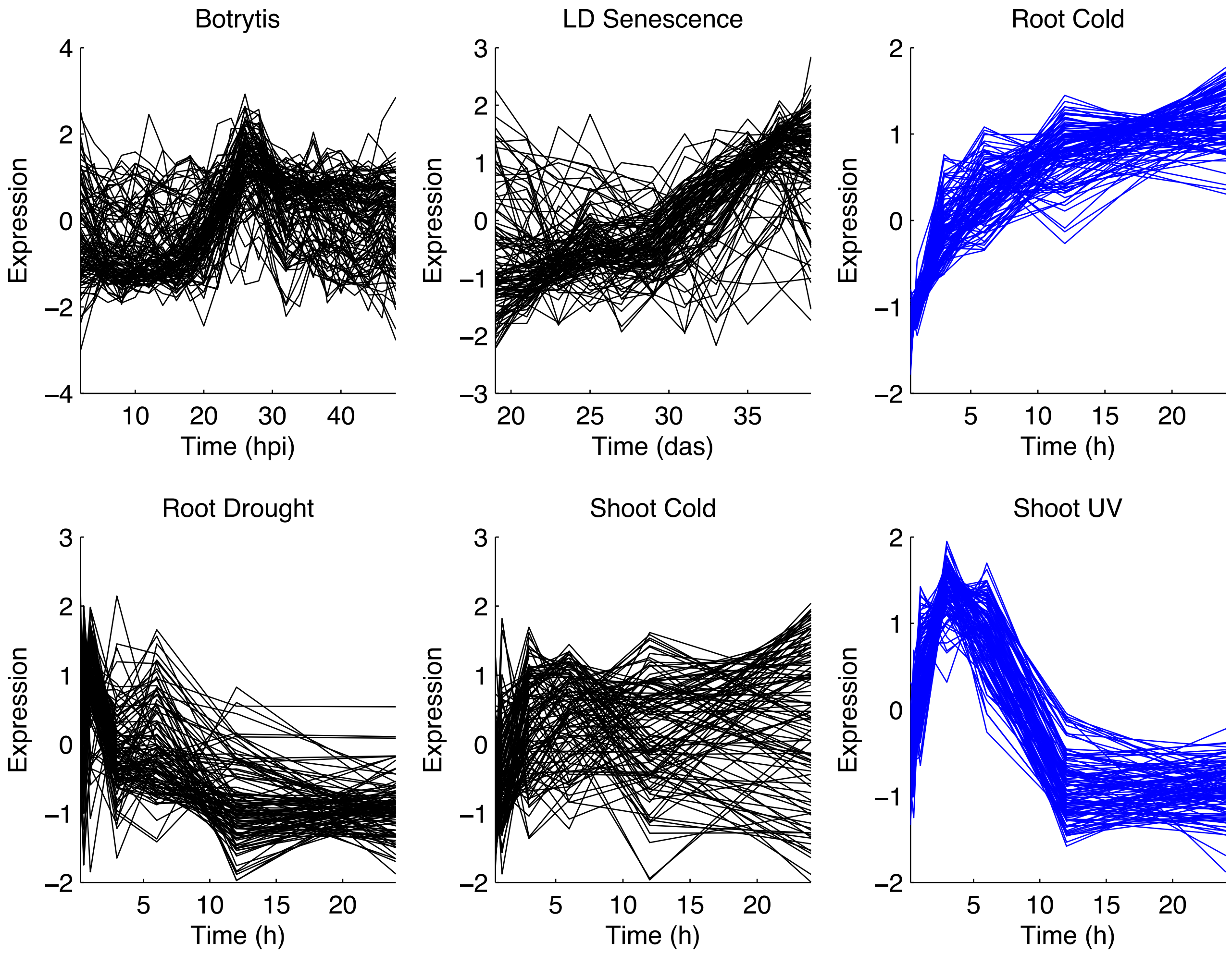
Botrytis
Expression
Time (hpi)
LD Senescence
Time (das)
Root Cold
Time (h)
Root Drought
Shoot Cold
Shoot UV

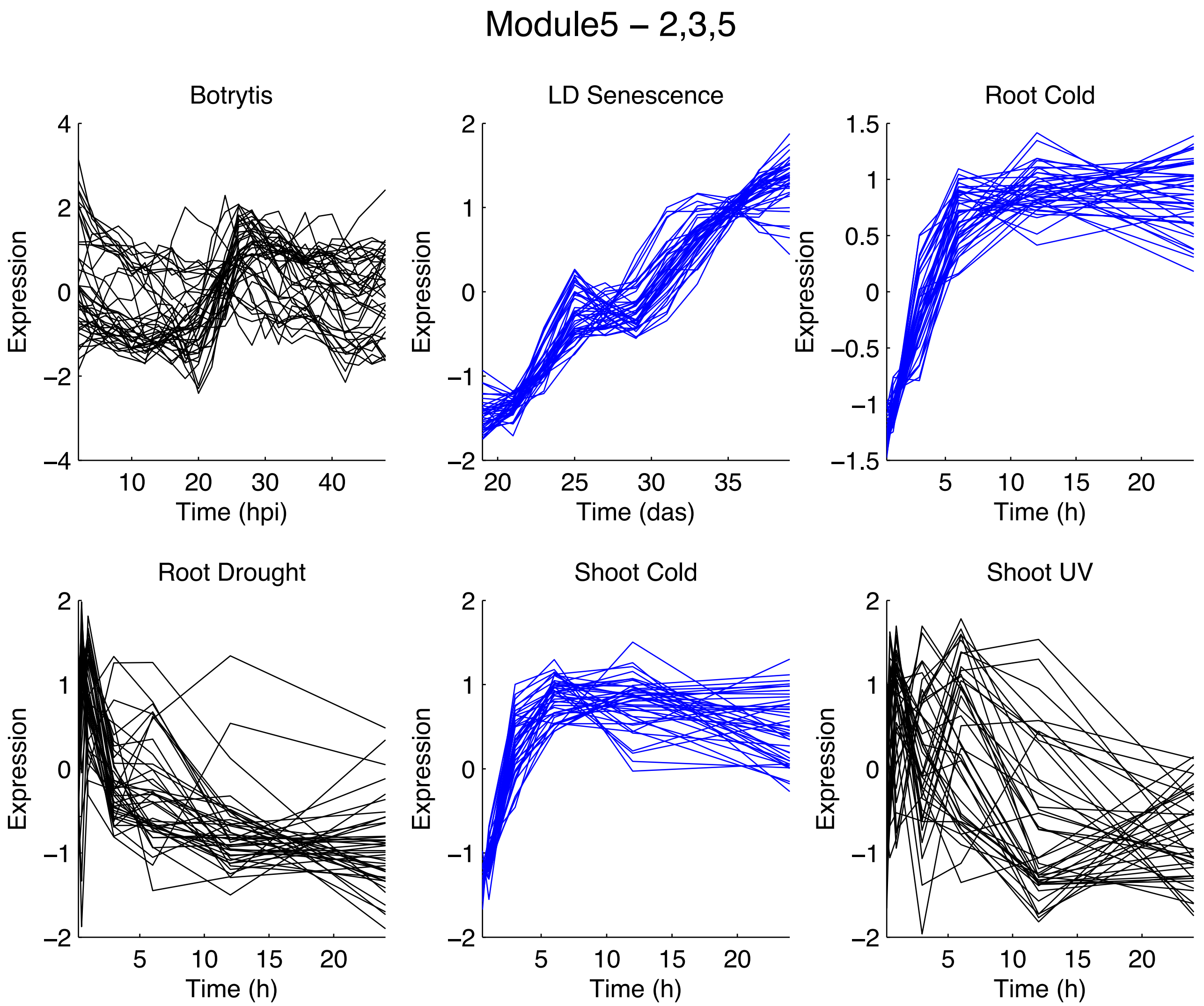
Module5 – 2,3,5
Botrytis
LD Senescence
Root Cold
Root Drought
Shoot Cold
Shoot UV
Expression
Time (hpi)
Time (das)
Time (h)

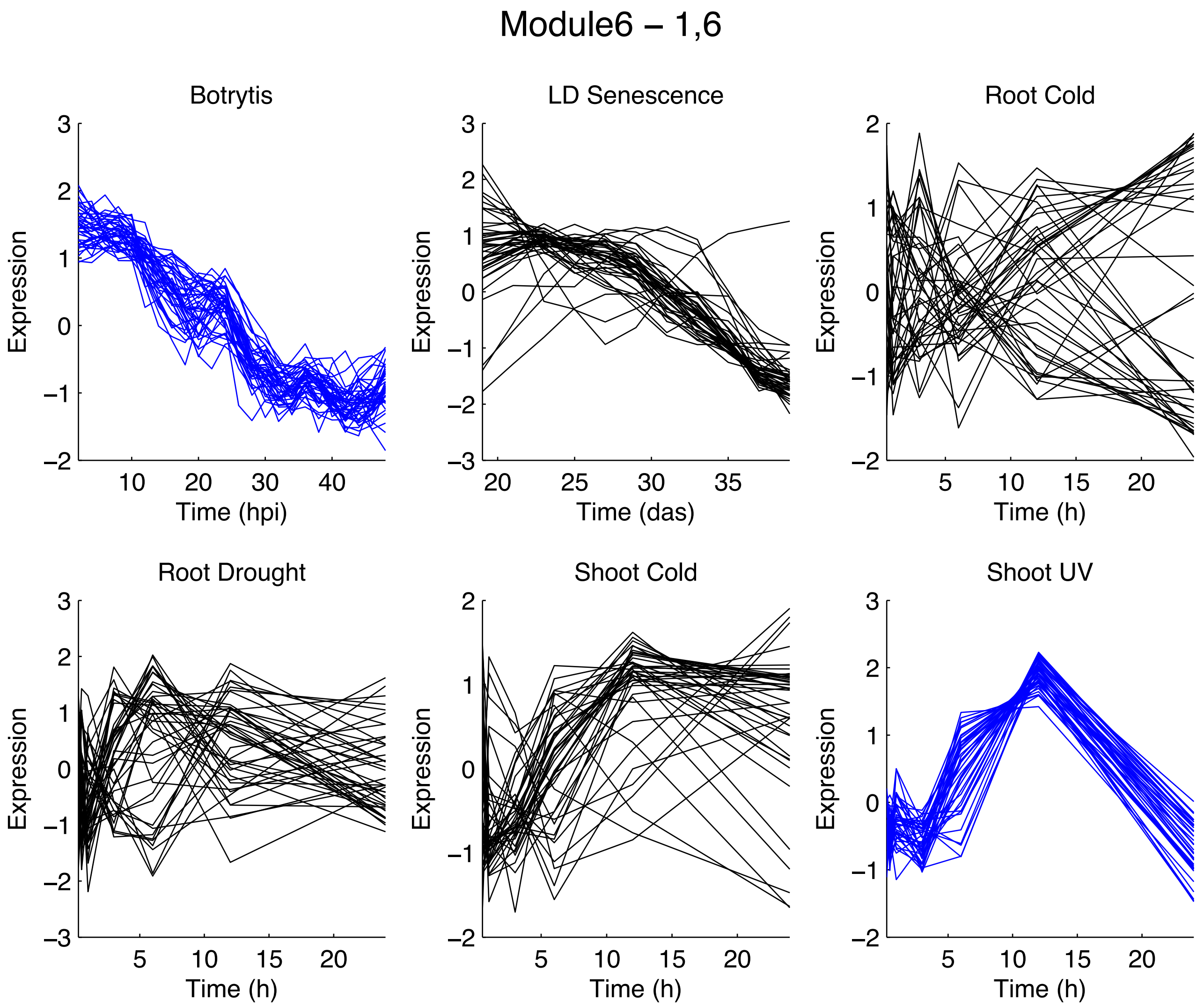

Module6 – 1,6
Botrytis
LD Senescence
Root Cold
Root Drought
Shoot Cold
Shoot UV
Expression
Time (hpi)
Time (das)
Time (h)

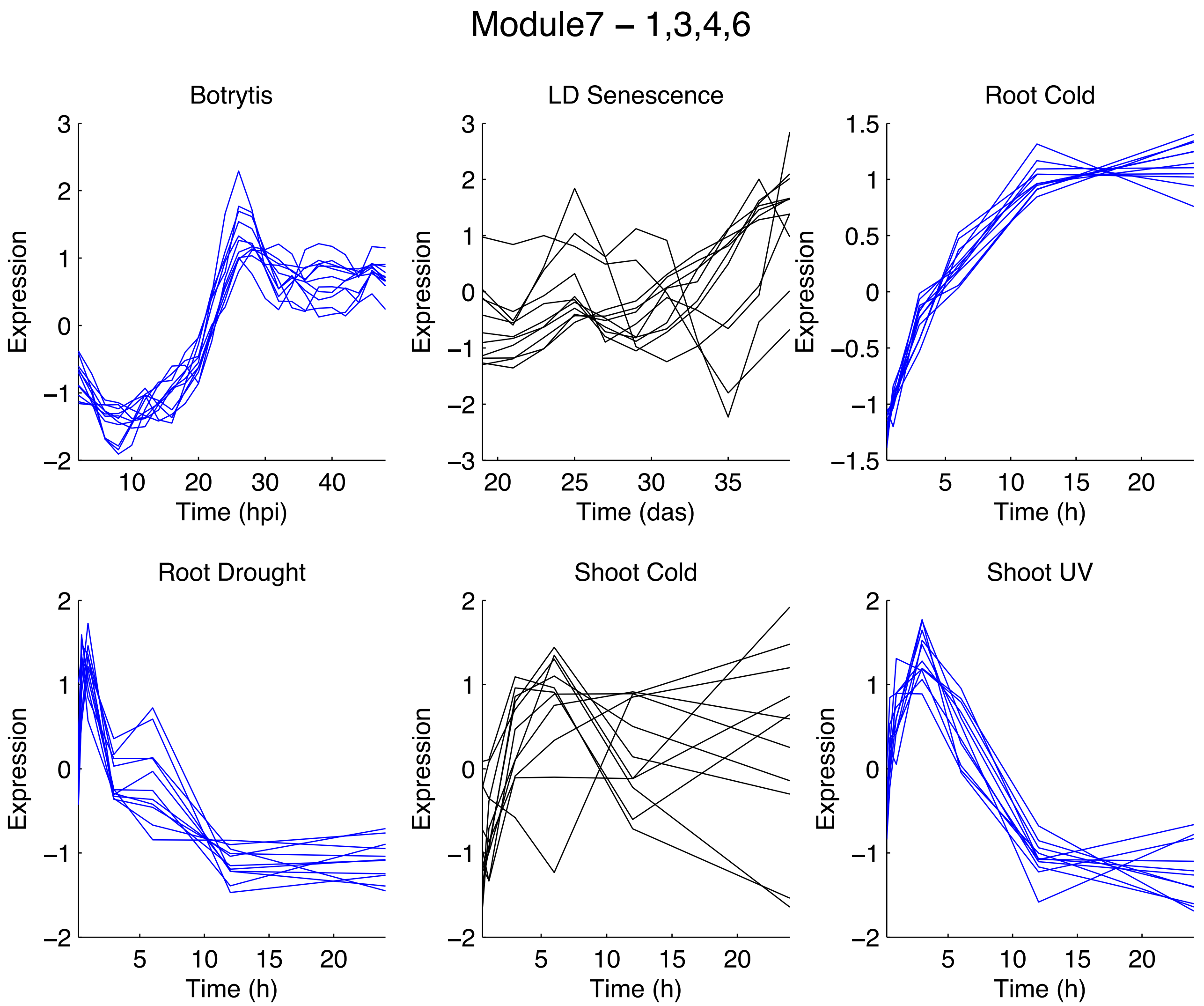
Module7 – 1,3,4,6
Botrytis
LD Senescence
Root Cold
Root Drought
Shoot Cold
Shoot UV
Expression
Time (hpi)
Time (das)
Time (h)

Module8 – 3,4,6

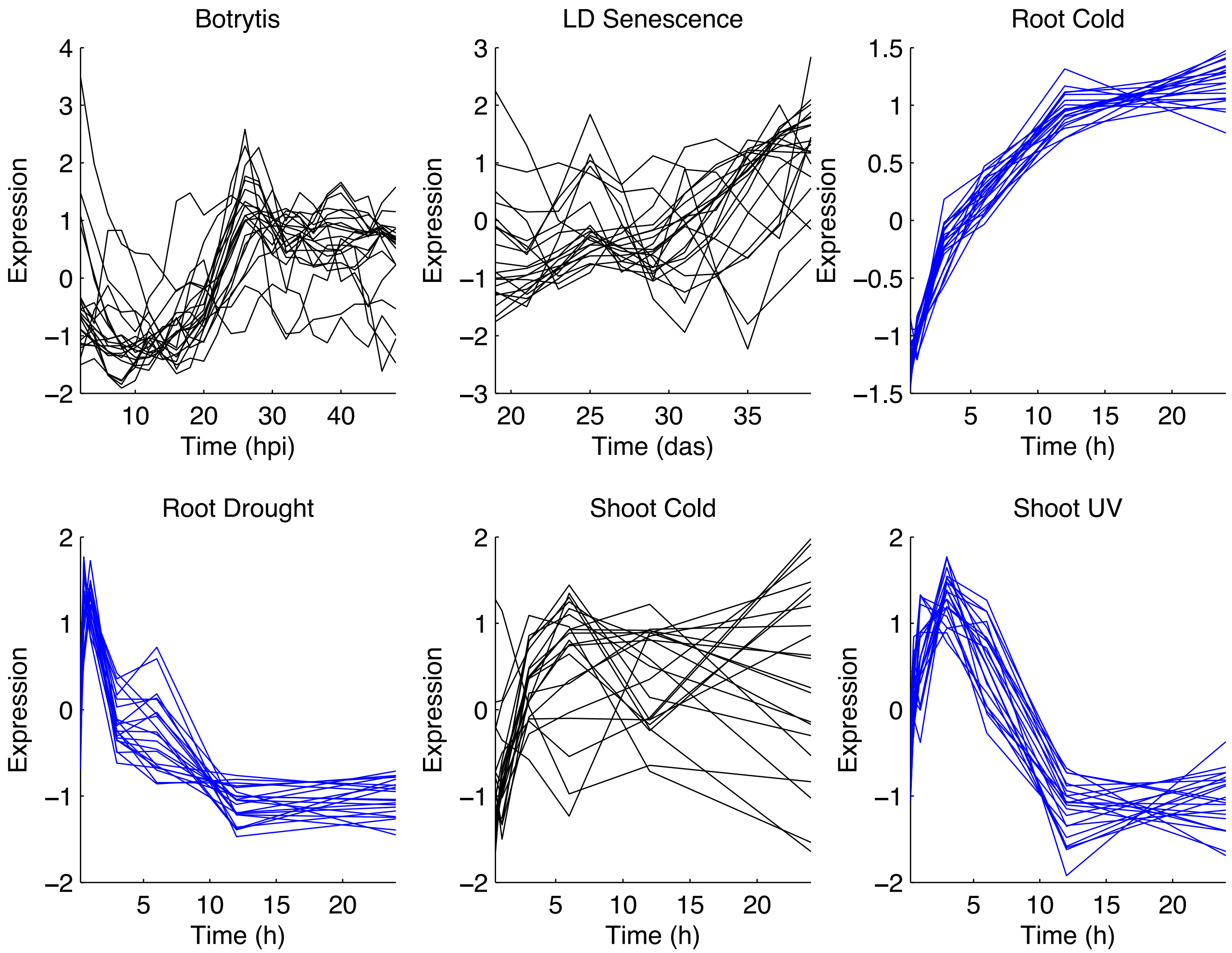

Botrytis
LD Senescence
Root Cold
Root Drought
Shoot Cold
Shoot UV
Expression
Time (hpi)
Time (das)
Time (h)

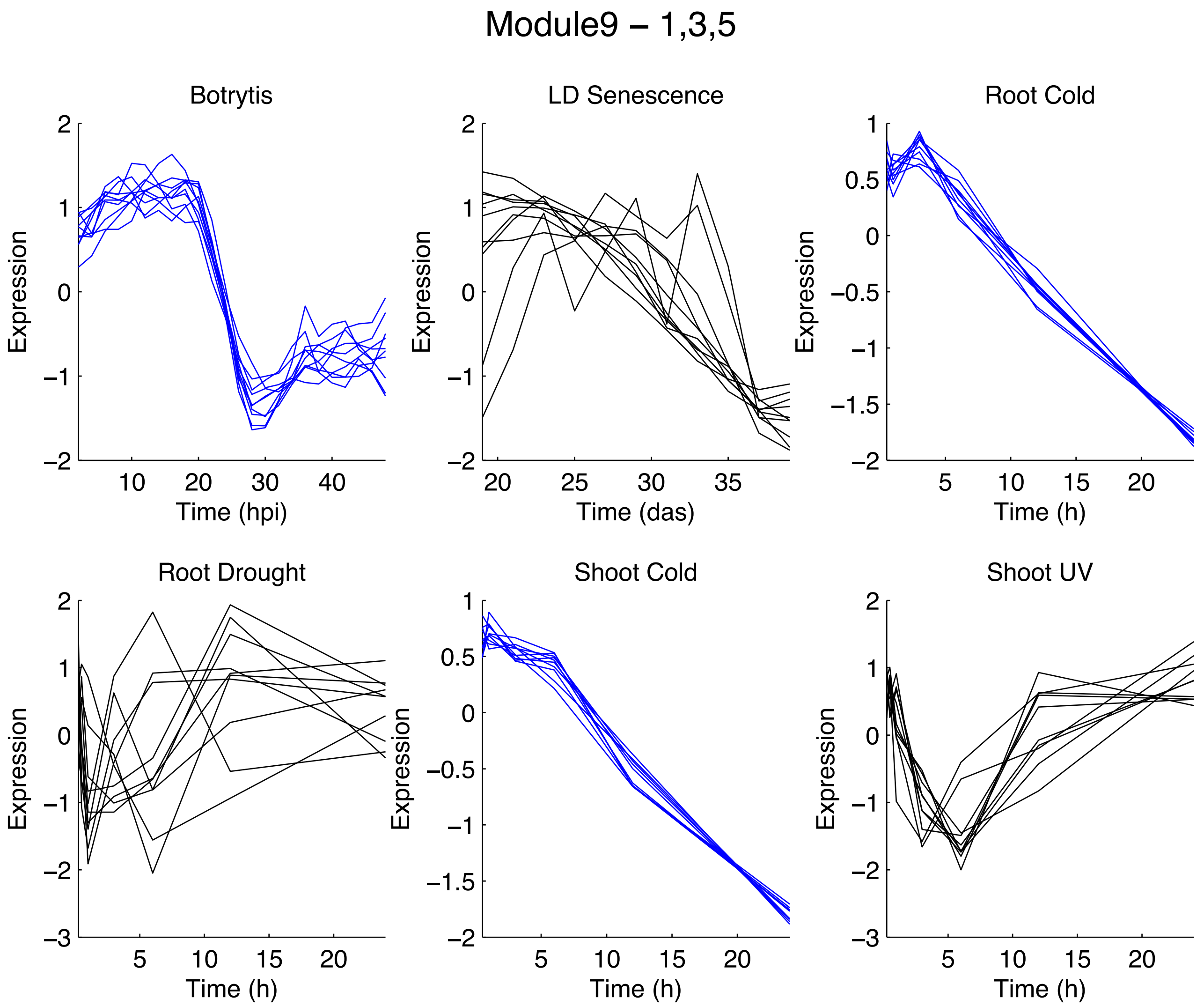
Module9 – 1,3,5
Botrytis
LD Senescence
Root Cold
Root Drought
Shoot Cold
Shoot UV
Expression
Time (hpi)
Time (das)
Time (h)
2
1
0
−1
−2
−3
0.5
−0.5
−1.5
10
20
30
40
25
35
5
15

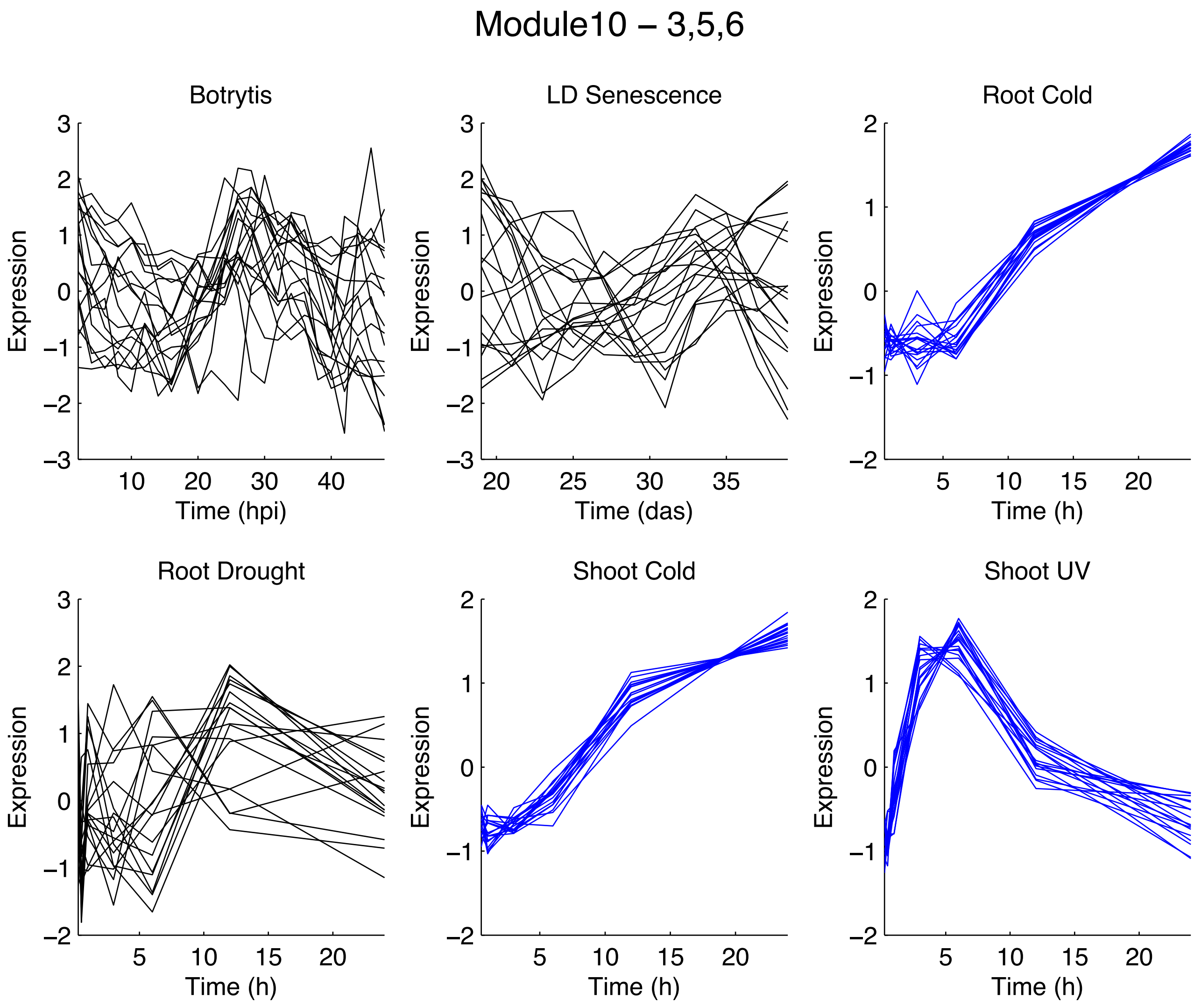

Module10 – 3,5,6
Botrytis
LD Senescence
Root Cold
Root Drought
Shoot Cold
Shoot UV
Expression
Time (hpi)
Time (das)
Time (h)

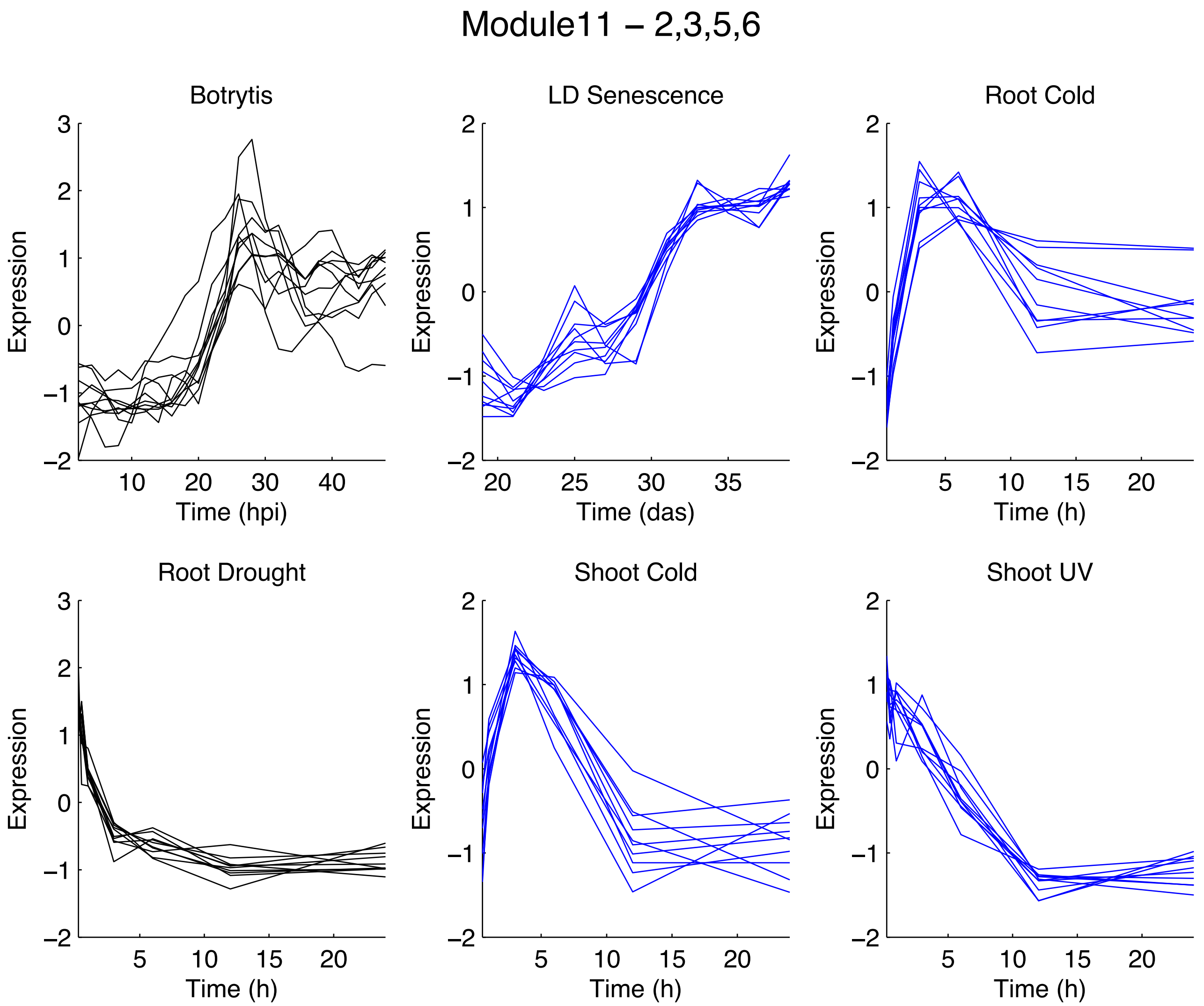

Module11 – 2,3,5,6
Botrytis
LD Senescence
Root Cold
Root Drought
Shoot Cold
Shoot UV
Expression
Time (hpi)
Time (das)
Time (h)

Module12 – 1,2,6

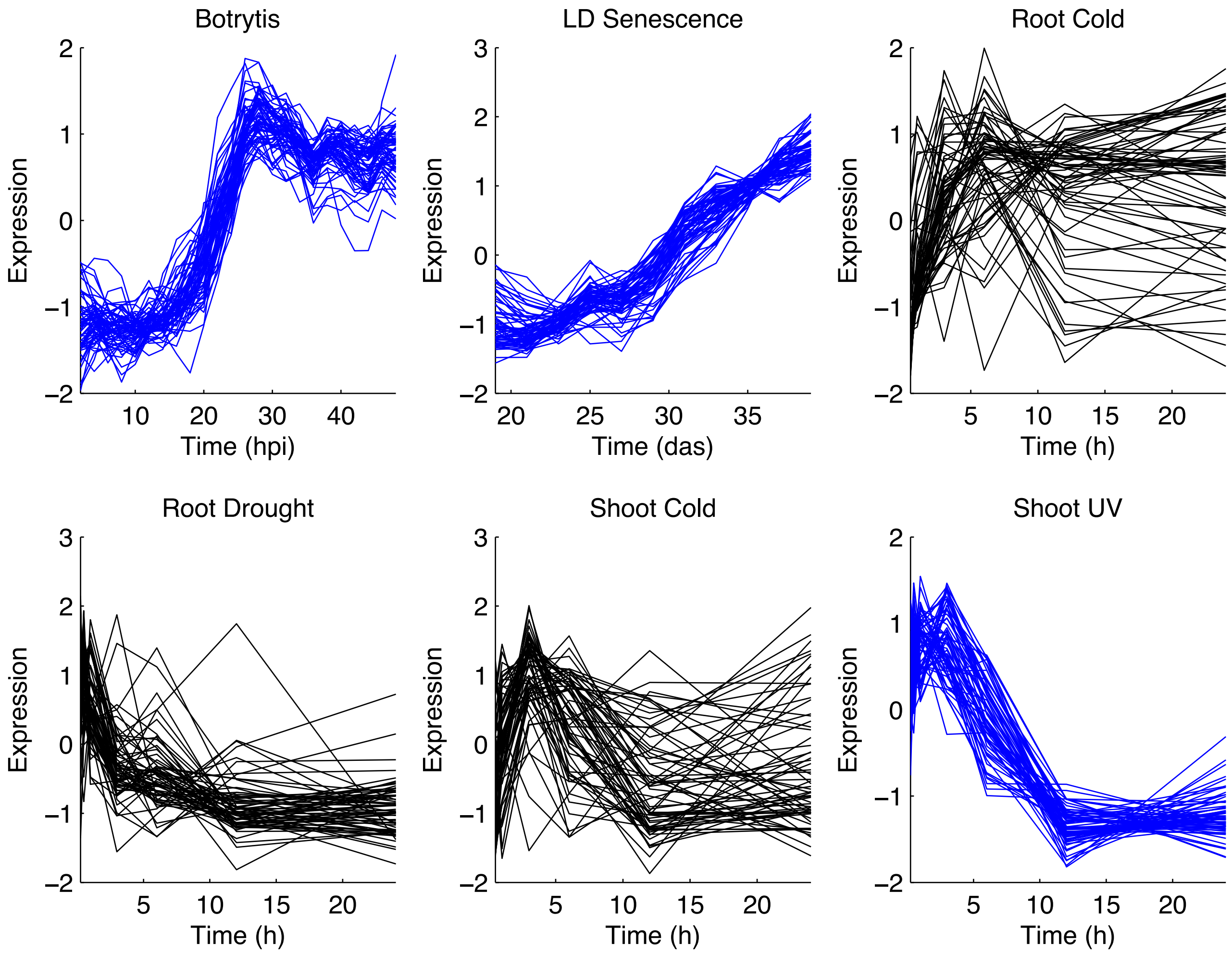

Module13 – 5,6

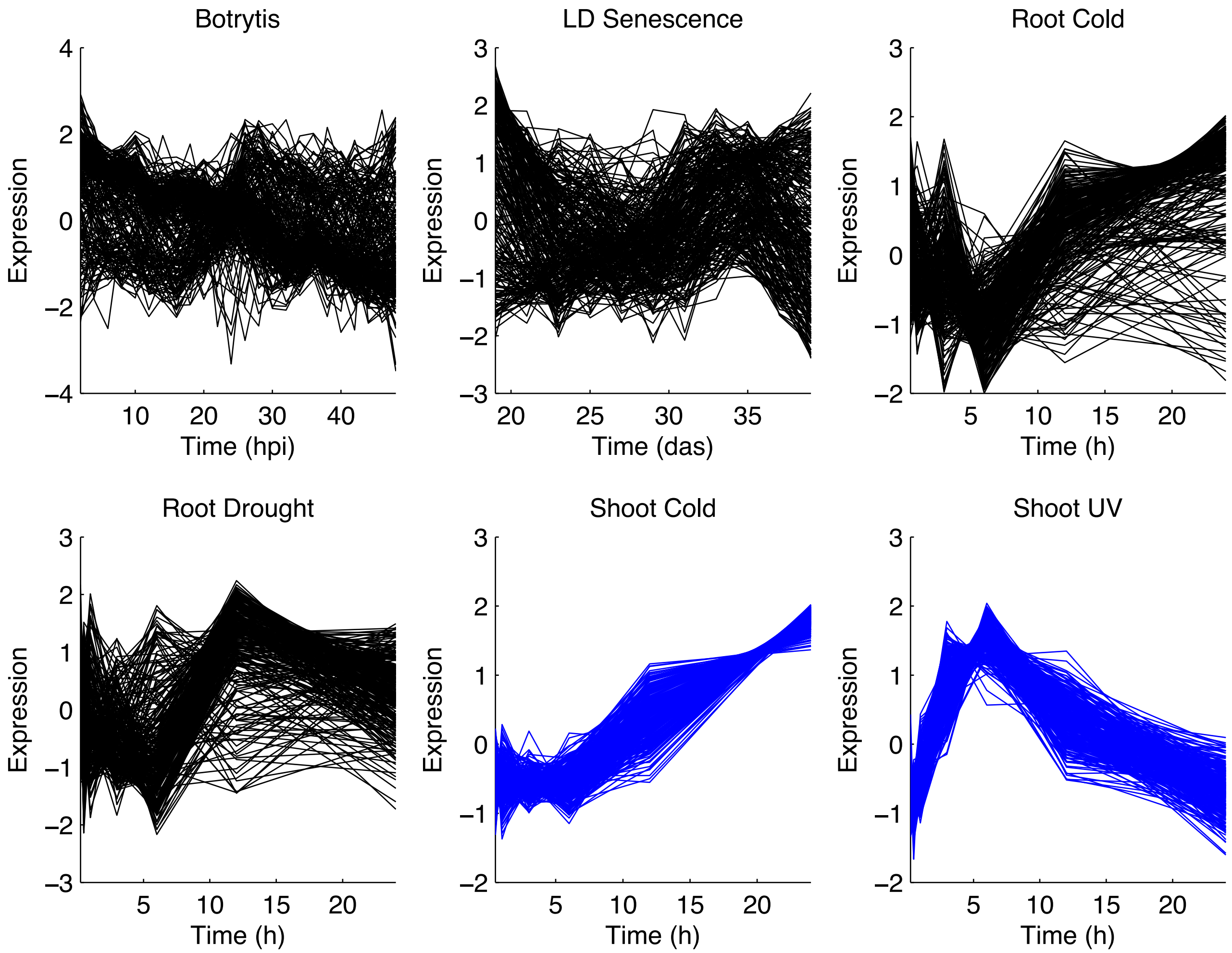

Botrytis
Expression
Time (hpi)
LD Senescence
Expression
Time (das)
Root Cold
Expression
Time (h)
Root Drought
Expression
Time (h)
Shoot Cold
Expression
Time (h)
Shoot UV
Expression
Time (h)

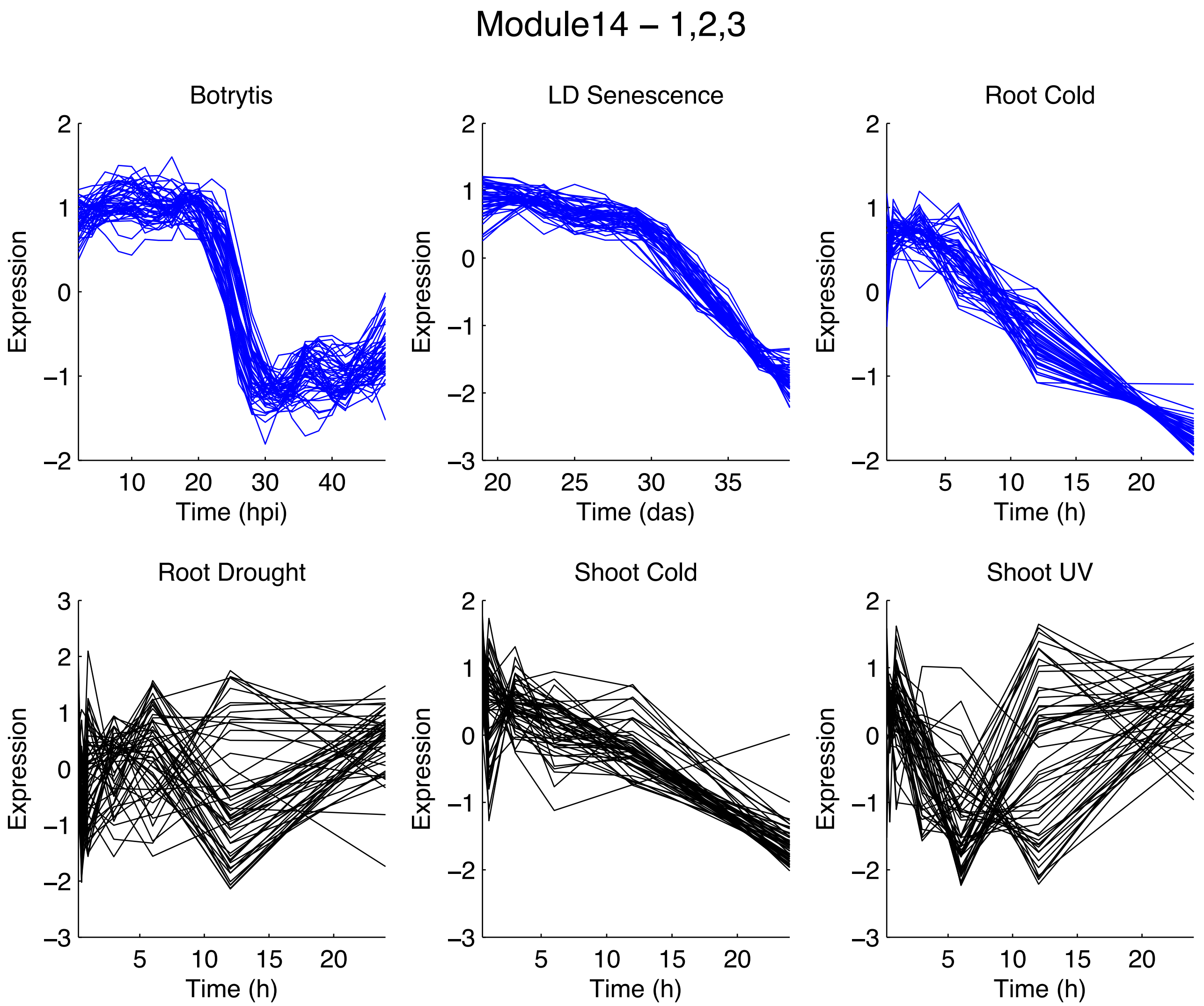

Module14 – 1,2,3
Botrytis
LD Senescence
Root Cold
Root Drought
Shoot Cold
Shoot UV
Expression
Time (hpi)
Time (das)
Time (h)

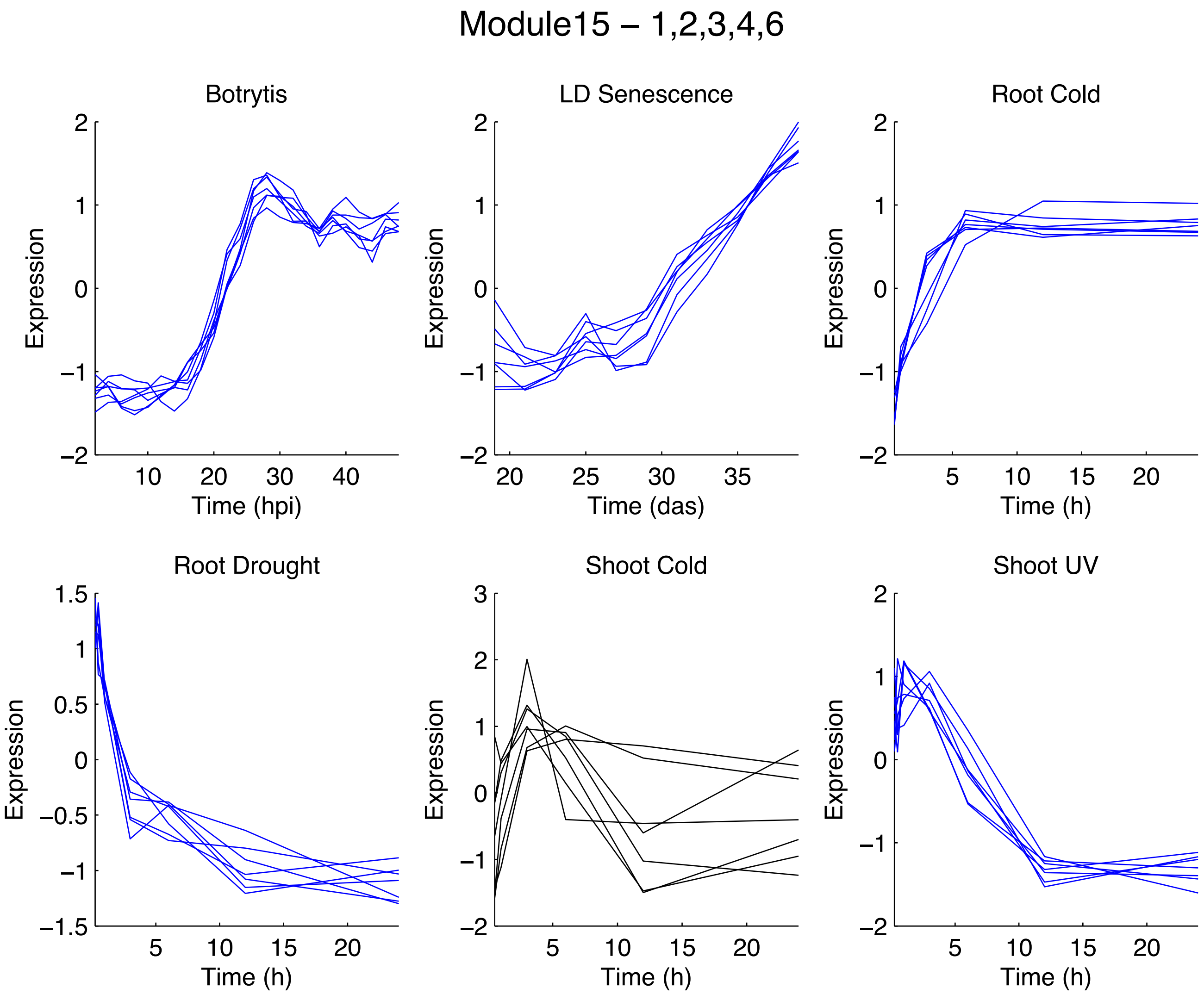

Module15 – 1,2,3,4,6
Botrytis
LD Senescence
Root Cold
Root Drought
Shoot Cold
Shoot UV
Expression
Time (hpi)
Time (das)
Time (h)

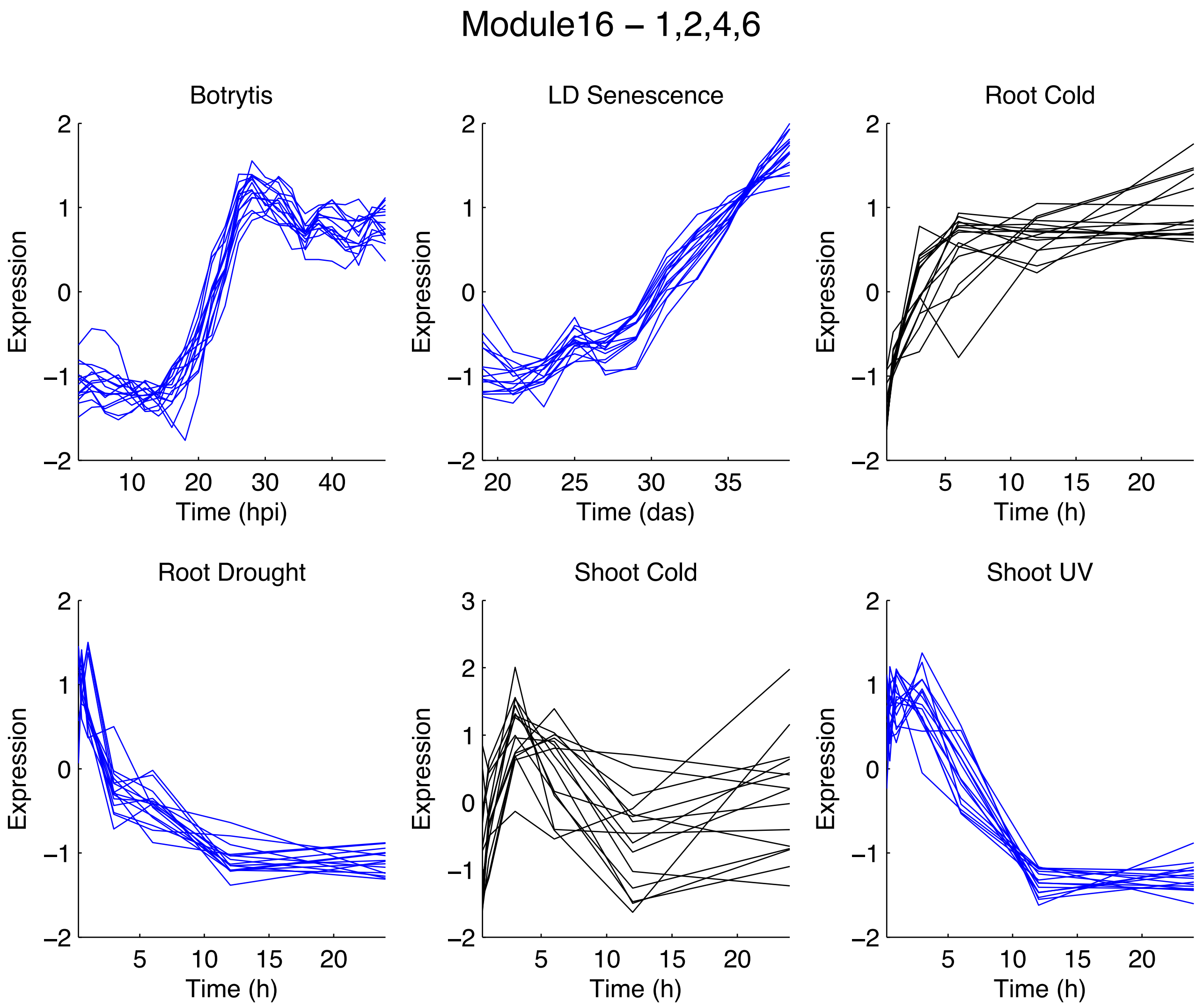
Module16 – 1,2,4,6
Botrytis
LD Senescence
Root Cold
Root Drought
Shoot Cold
Shoot UV
Expression
Time (hpi)
Time (das)
Time (h)

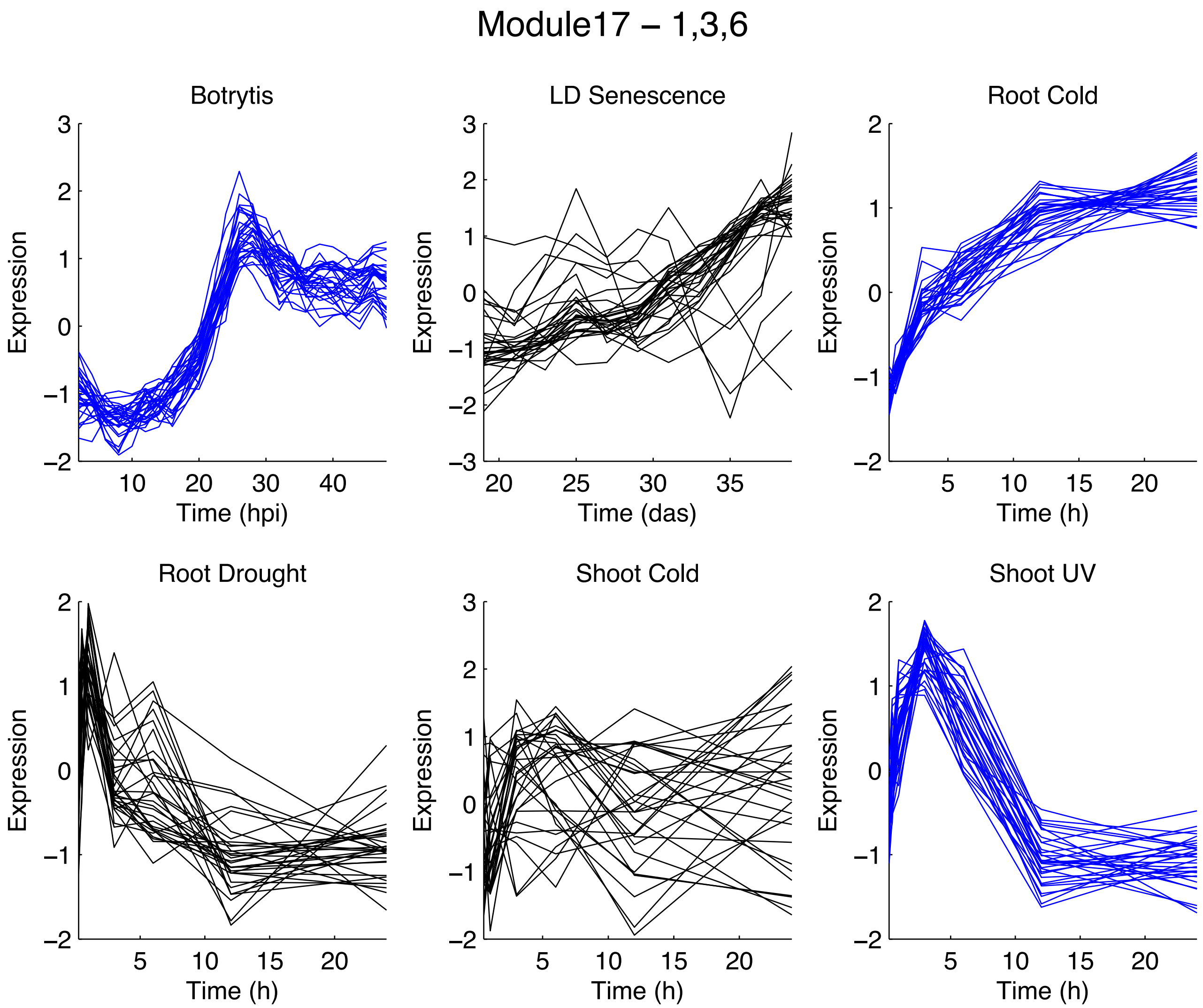
Module17 – 1,3,6
Botrytis
Expression
Time (hpi)
LD Senescence
Time (das)
Root Cold
Time (h)
Root Drought
Shoot Cold
Shoot UV

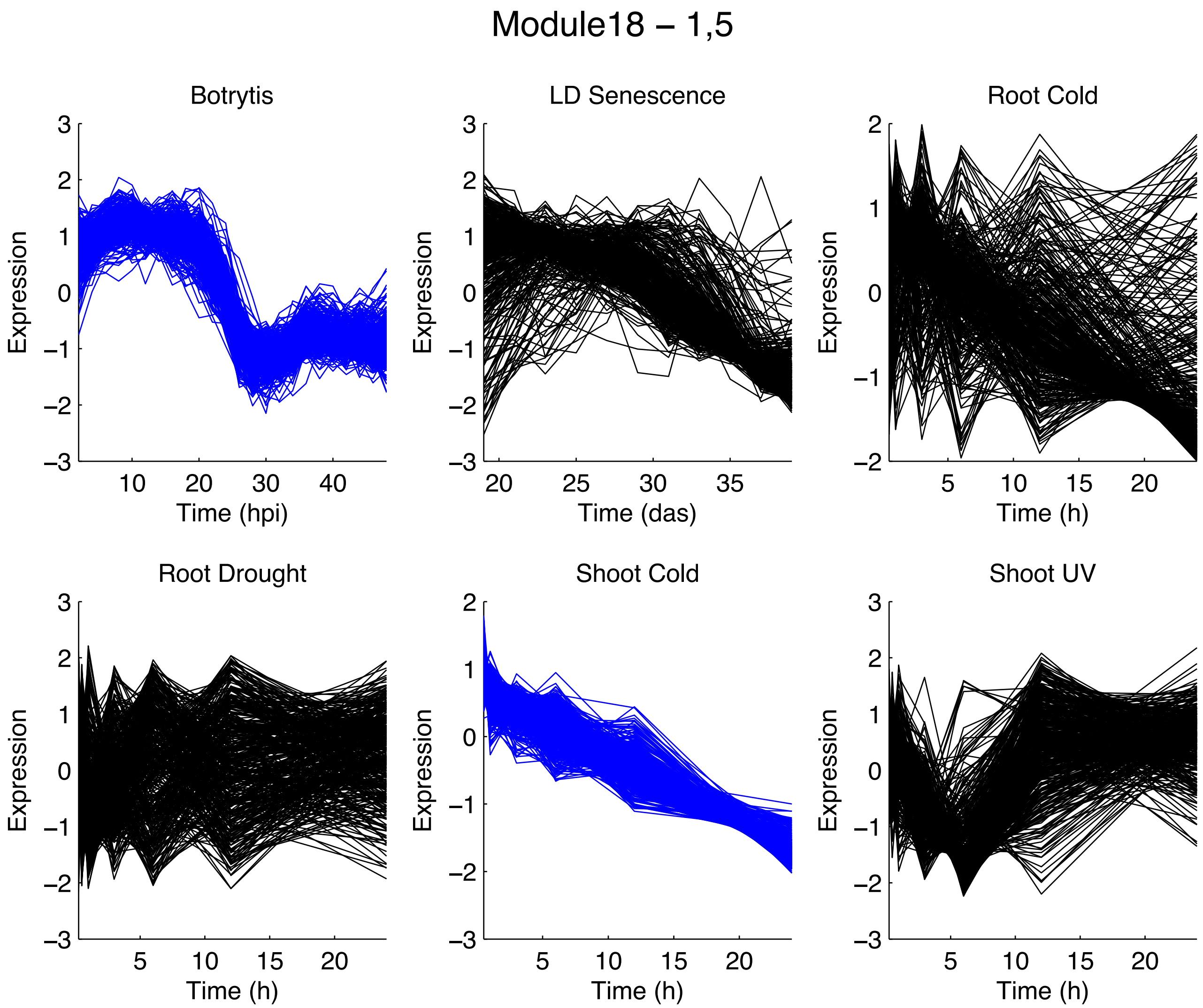
Module18 – 1,5
Botrytis
LD Senescence
Root Cold
Root Drought
Shoot Cold
Shoot UV
Expression
Time (hpi)
Time (das)
Time (h)

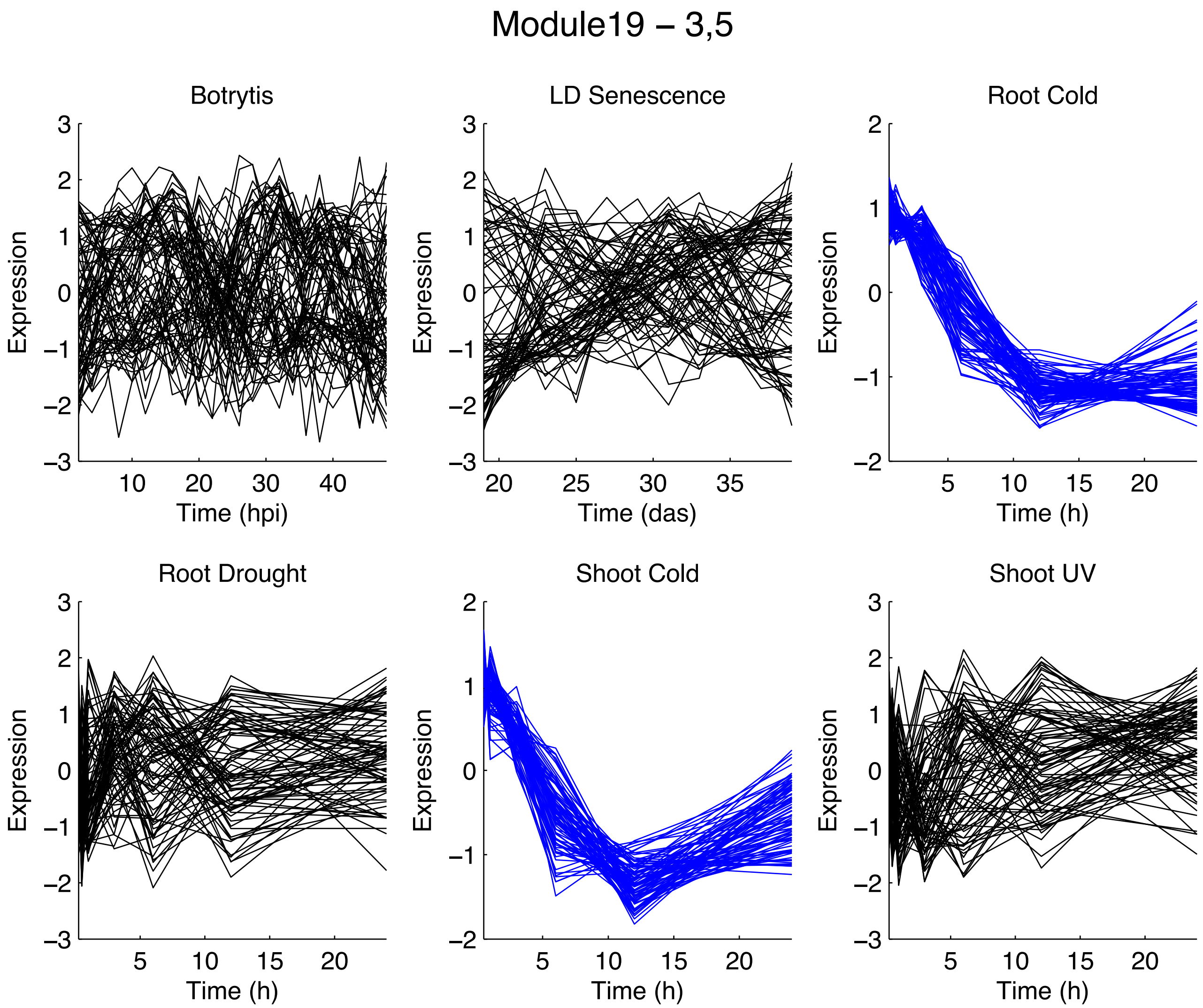
Module19 – 3,5
Botrytis
LD Senescence
Root Cold
Root Drought
Shoot Cold
Shoot UV
Expression
Time (hpi)
Time (das)
Time (h)

Module20 – 1,5,6

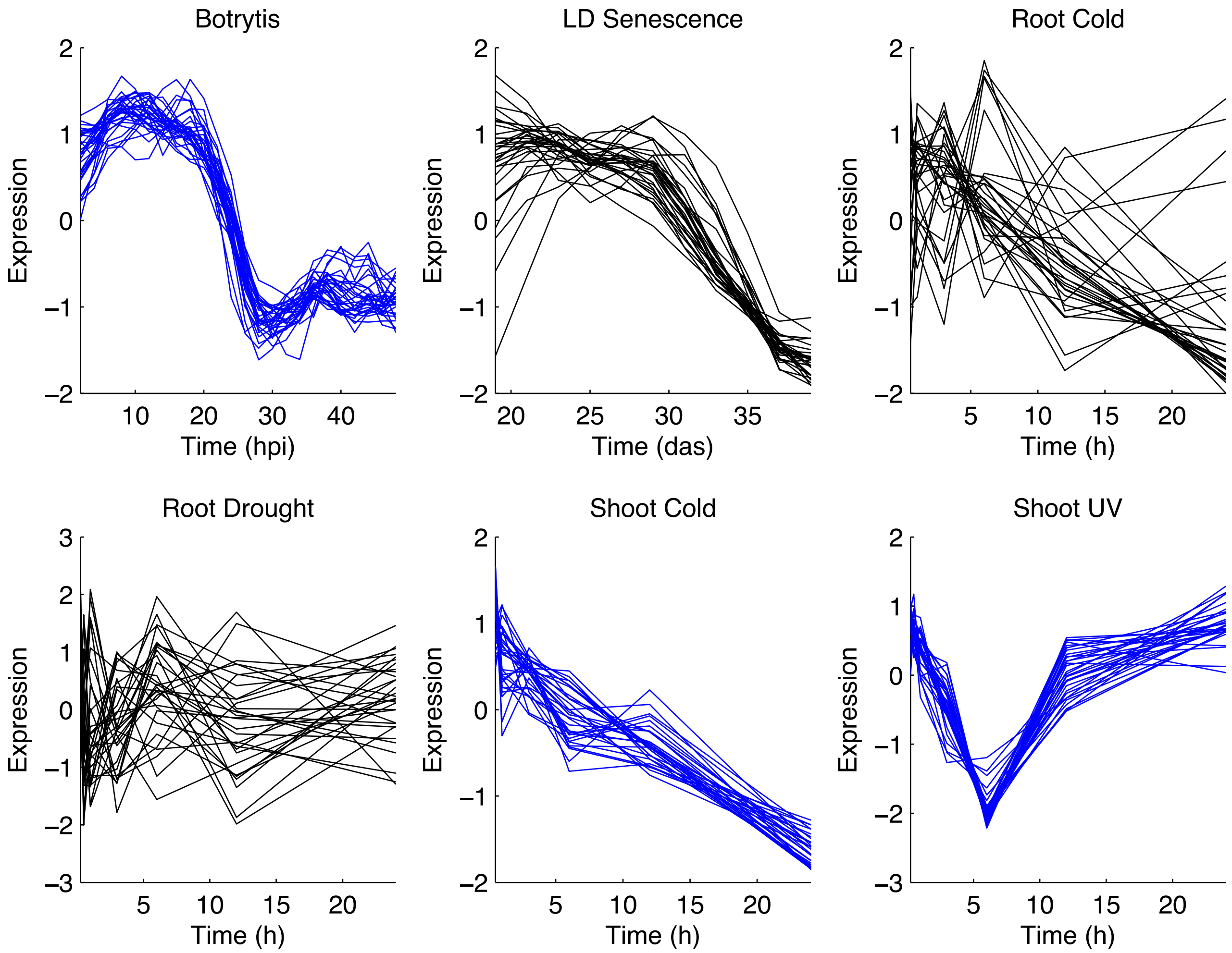

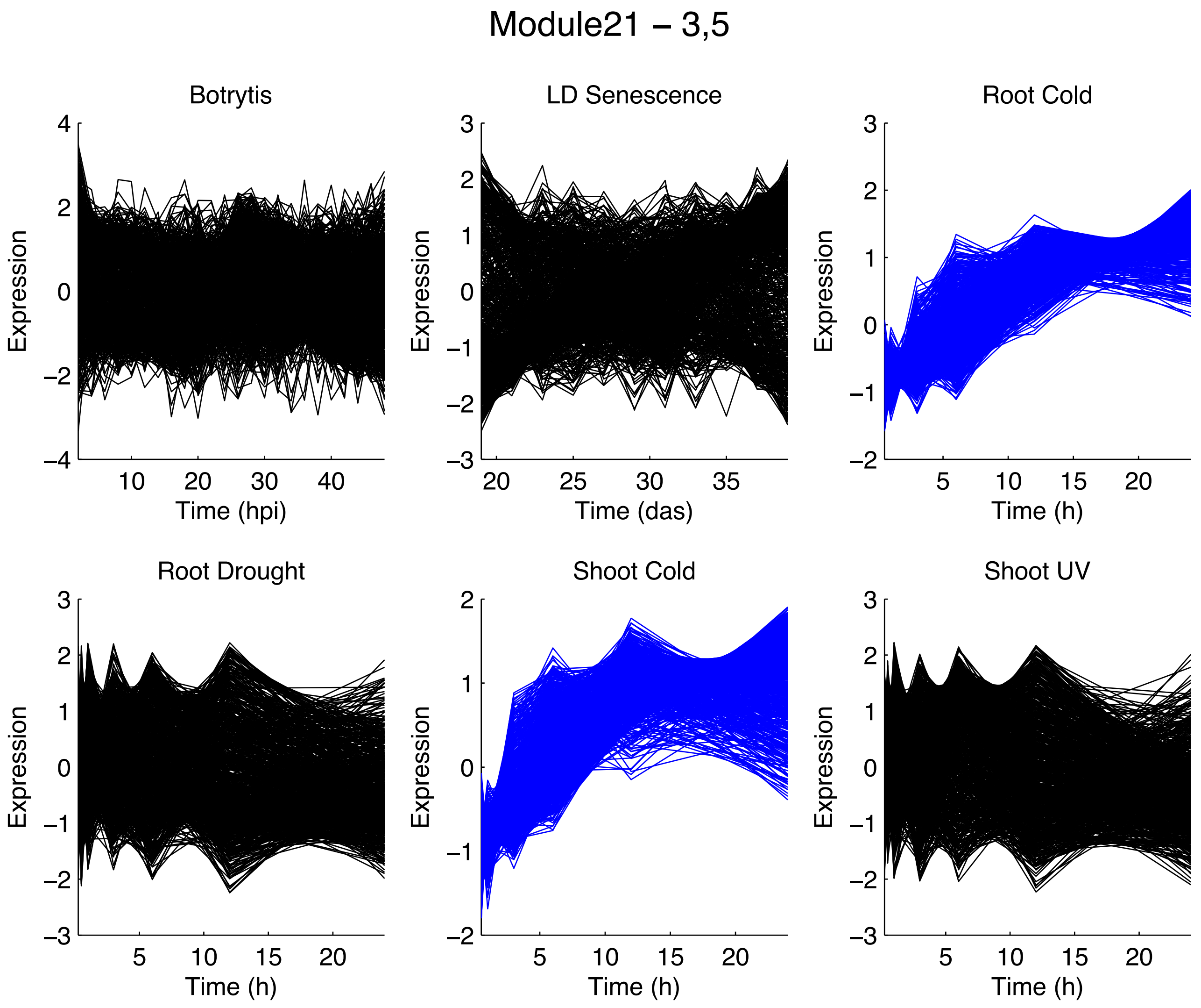
Module21 – 3,5
Botrytis
LD Senescence
Root Cold
Root Drought
Shoot Cold
Shoot UV
Expression
Time (hpi)
Time (das)
Time (h)
4
2
0
−2
−4
10
20
30
40
3
1
−1
−3
25
35
5
15

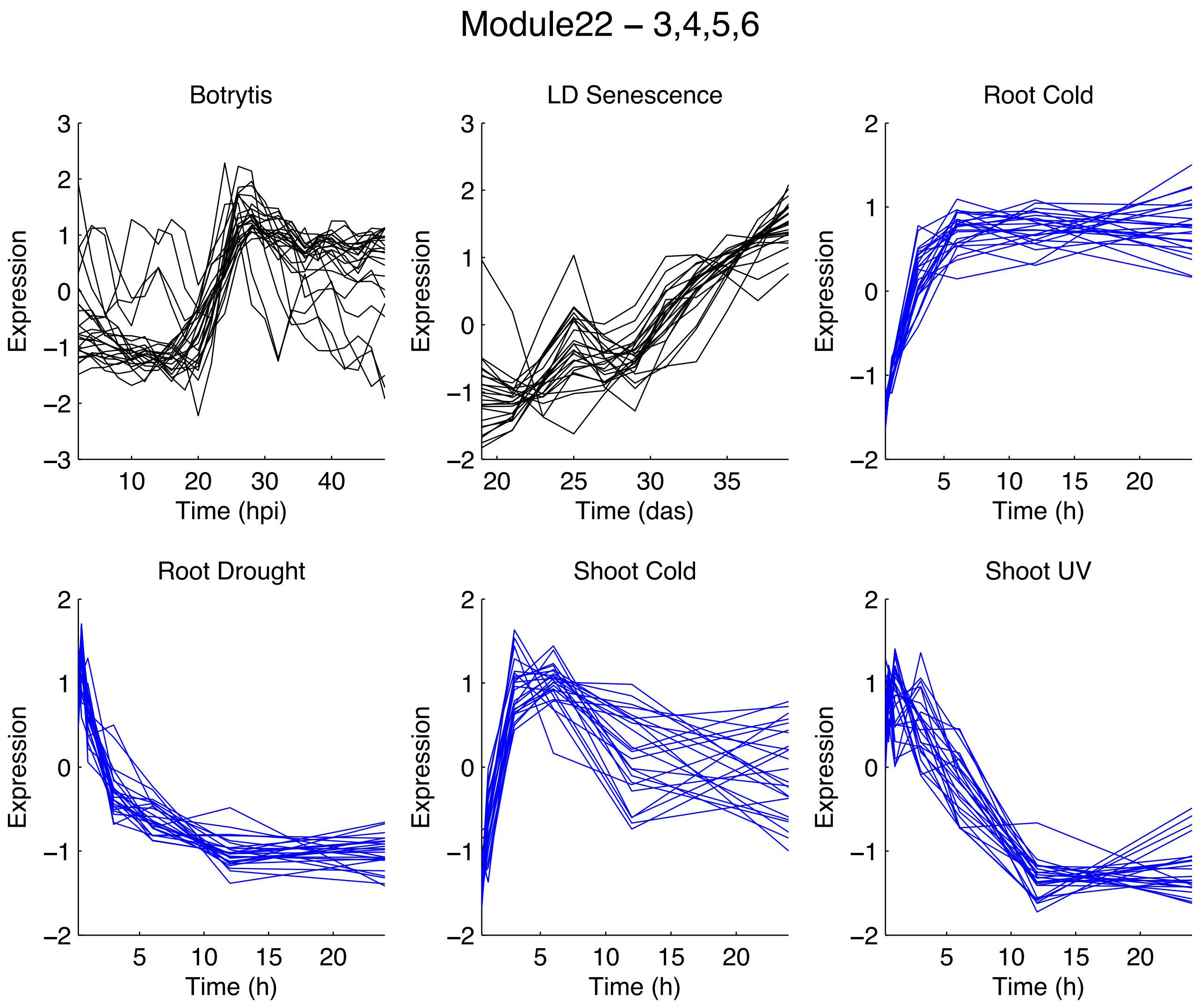

Module22 – 3,4,5,6
Botrytis
LD Senescence
Root Cold
Root Drought
Shoot Cold
Shoot UV
Expression
Time (hpi)
Time (das)
Time (h)
3
2
1
0
−1
−2
−3
10
20
30
40
25
35
5
15

Module23 – 5,6

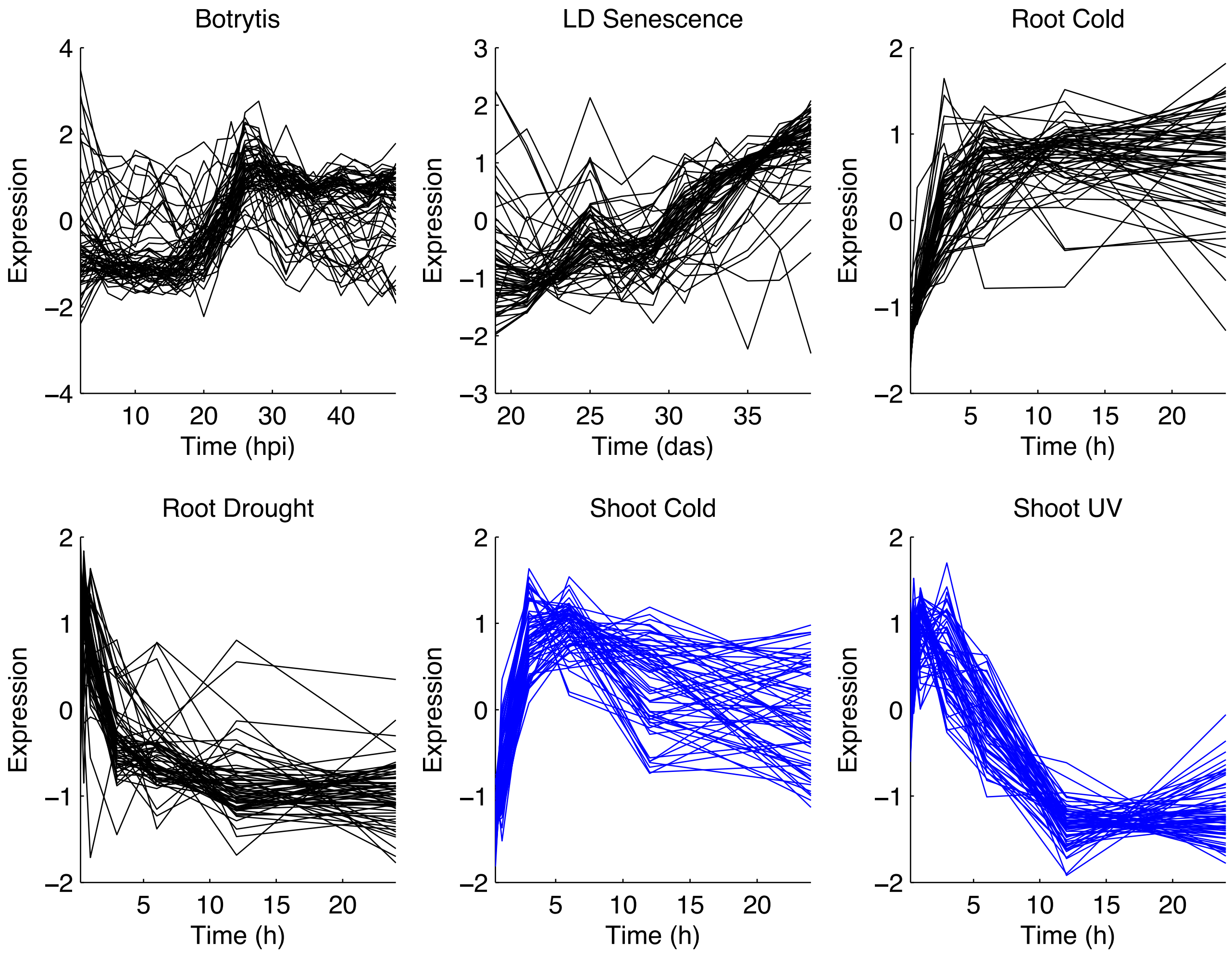
Botrytis
LD Senescence
Root Cold
Root Drought
Shoot Cold
Shoot UV
Expression
Time (hpi)
Time (das)
Time (h)
4
2
0
−2
−4
10
20
30
40
3
1
−1
−3
25
35
5
15

Module24 – 4,5

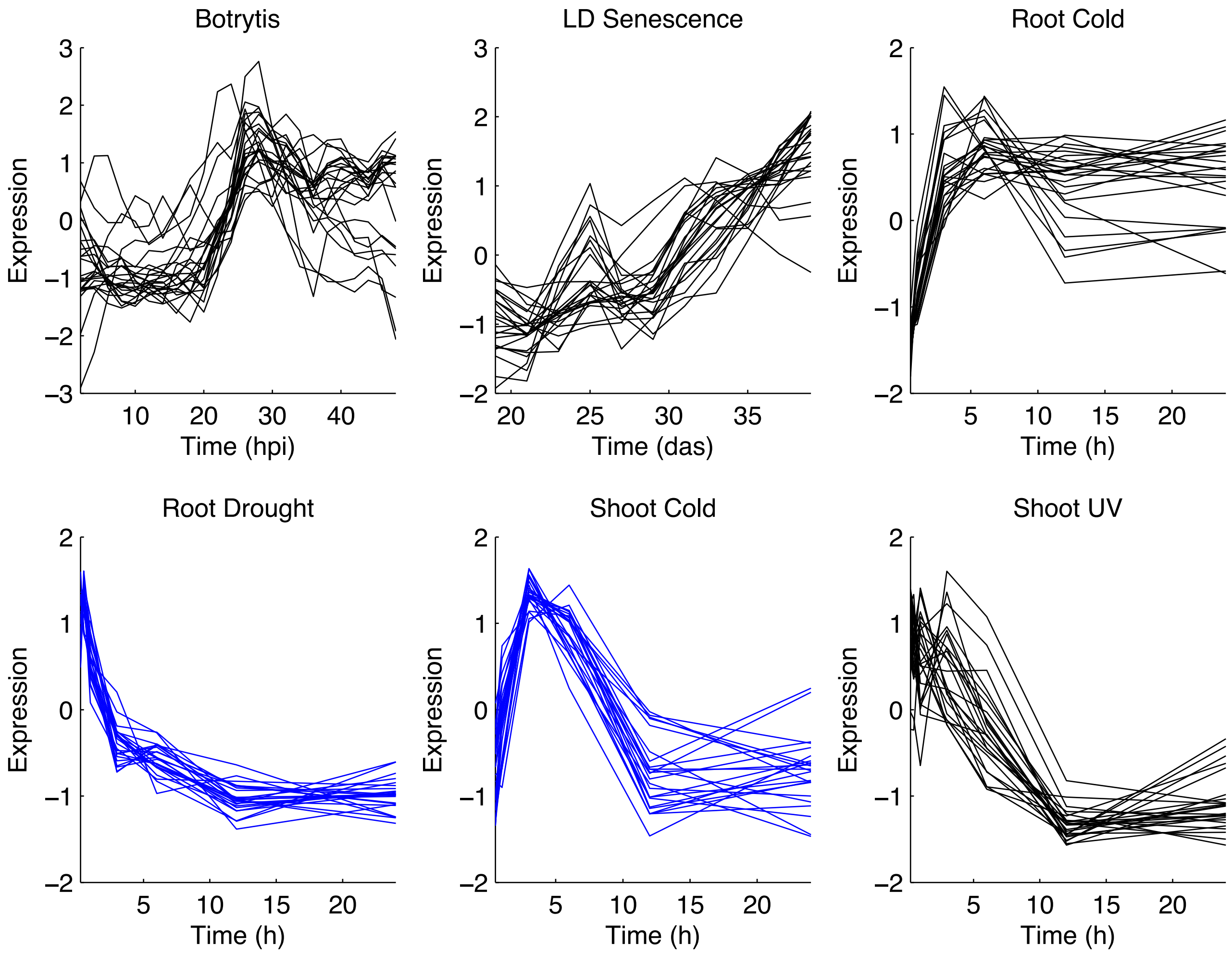
Botrytis
LD Senescence
Root Cold
Root Drought
Shoot Cold
Shoot UV
Expression
Time (hpi)
Time (das)
Time (h)
3
2
1
0
−1
−2
−3
10
20
30
40
25
35
5
15

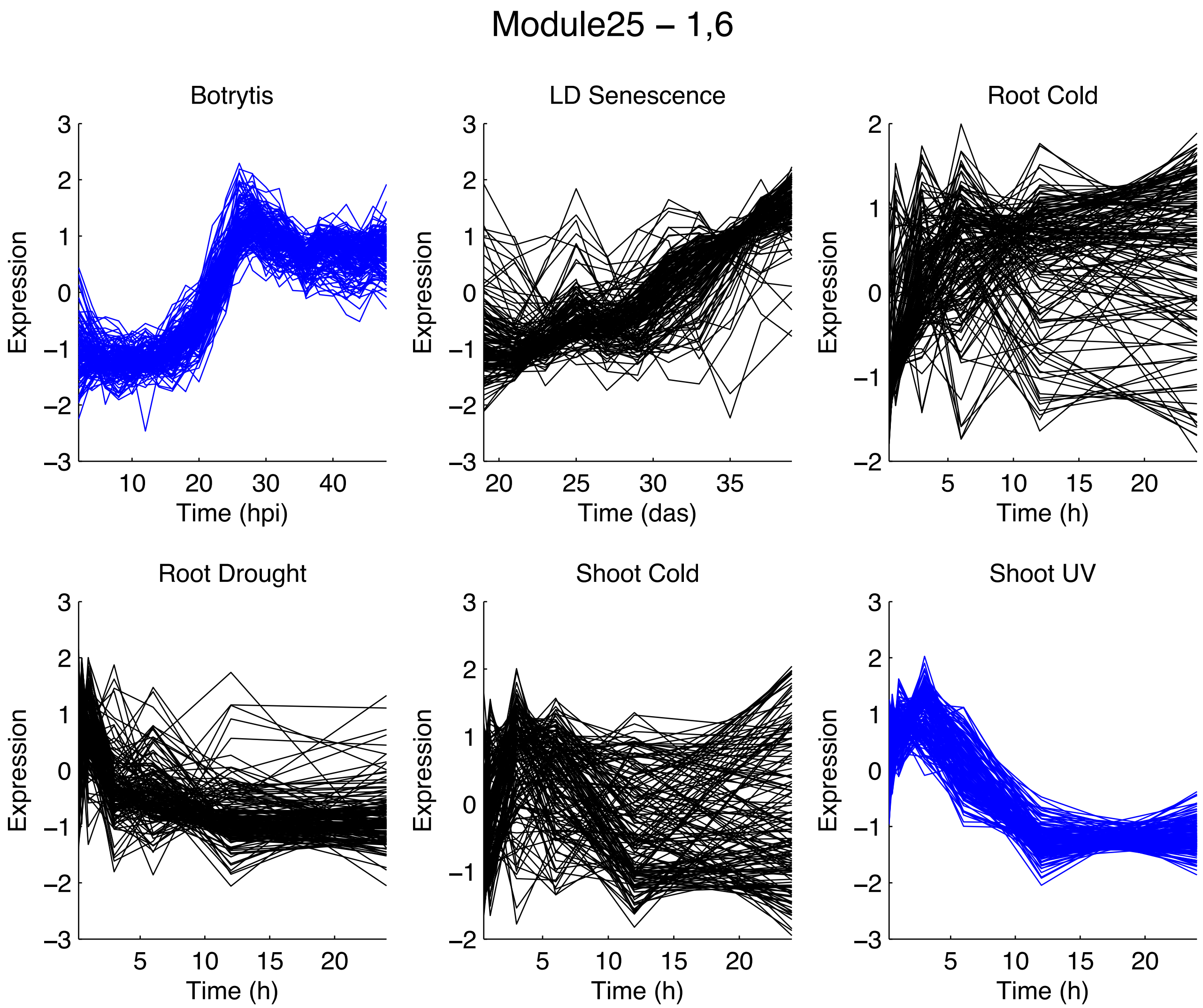
Module25 – 1,6
Botrytis
LD Senescence
Root Cold
Root Drought
Shoot Cold
Shoot UV
Expression
Time (hpi)
Time (das)
Time (h)

Module26 – 1,6

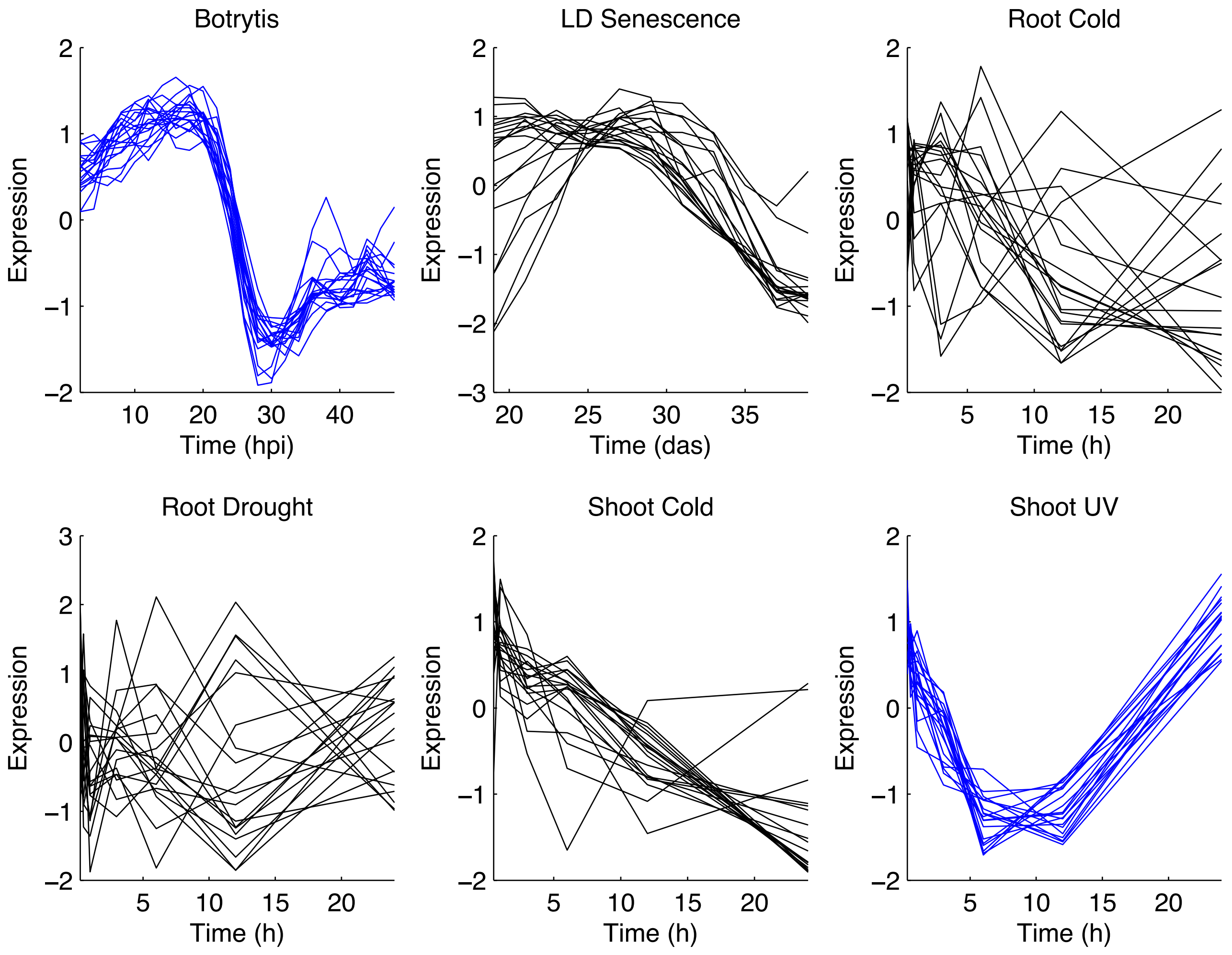
Botrytis
LD Senescence
Root Cold
Root Drought
Shoot Cold
Shoot UV
Expression
Time (hpi)
Time (das)
Time (h)

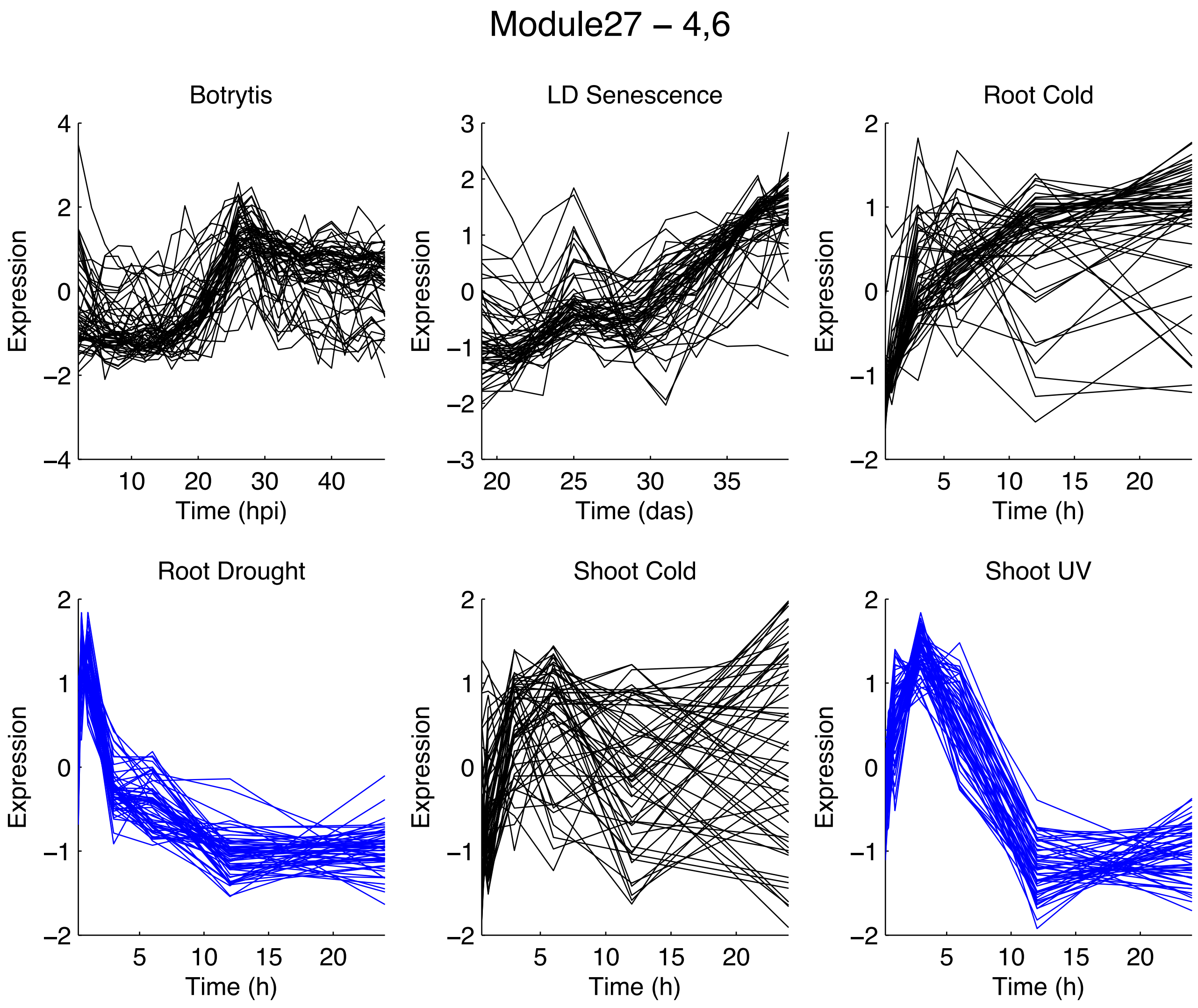
Module27 – 4,6
Botrytis
LD Senescence
Root Cold
Root Drought
Shoot Cold
Shoot UV
Expression
Time (hpi)
Time (das)
Time (h)

Module28 – 1,2,3,6

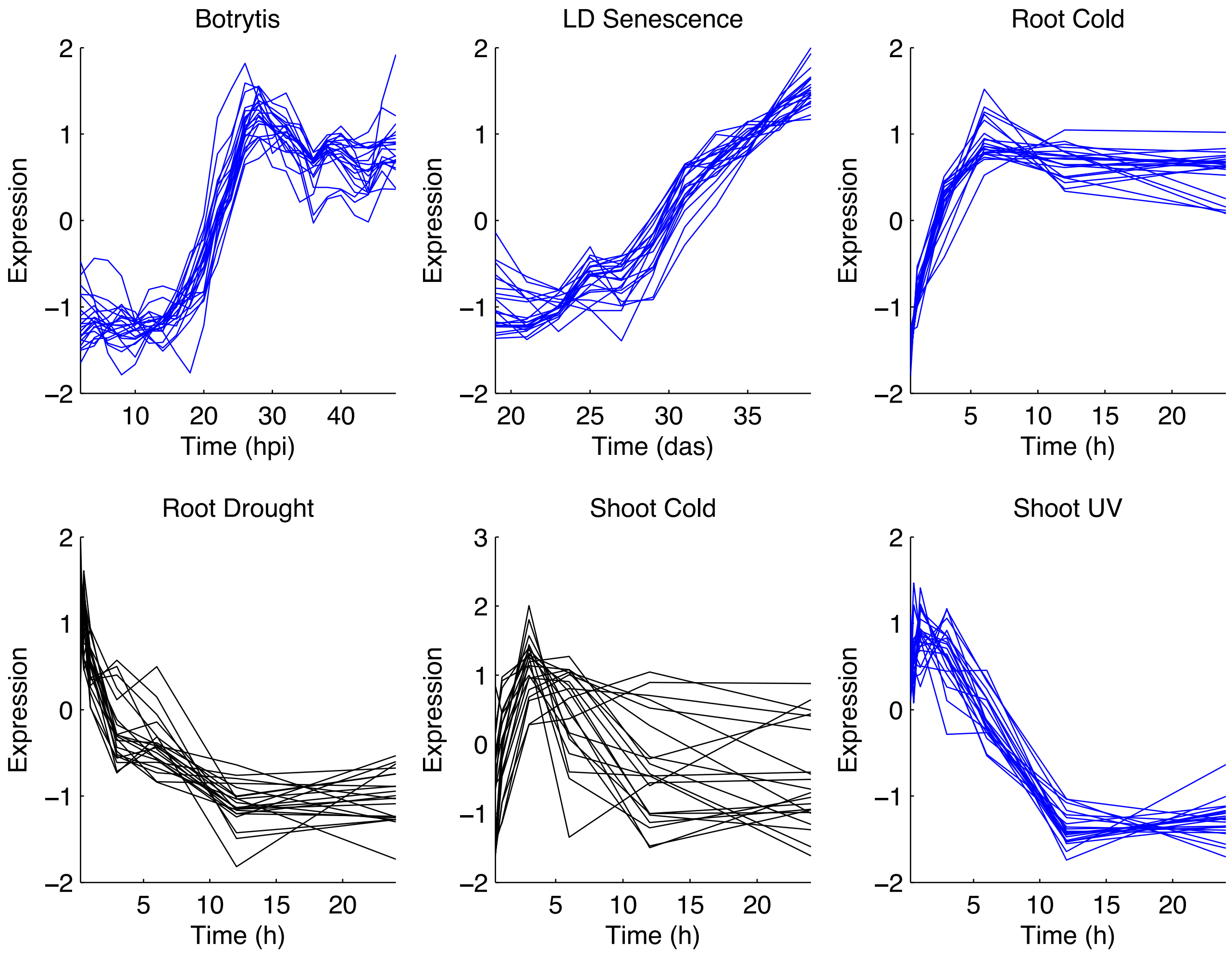

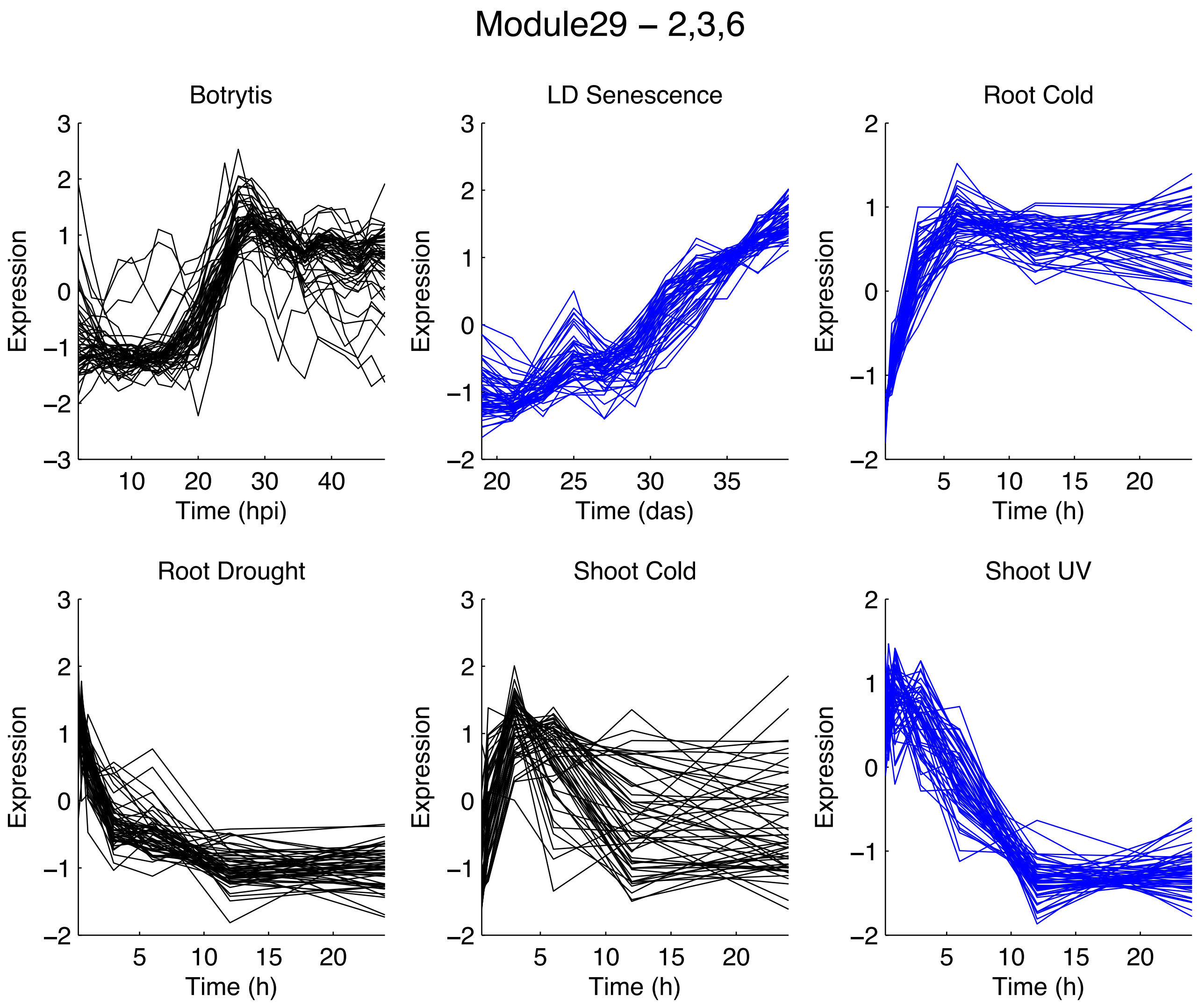

Module29 – 2,3,6
Botrytis
LD Senescence
Root Cold
Root Drought
Shoot Cold
Shoot UV
Expression
Time (hpi)
Time (das)
Time (h)

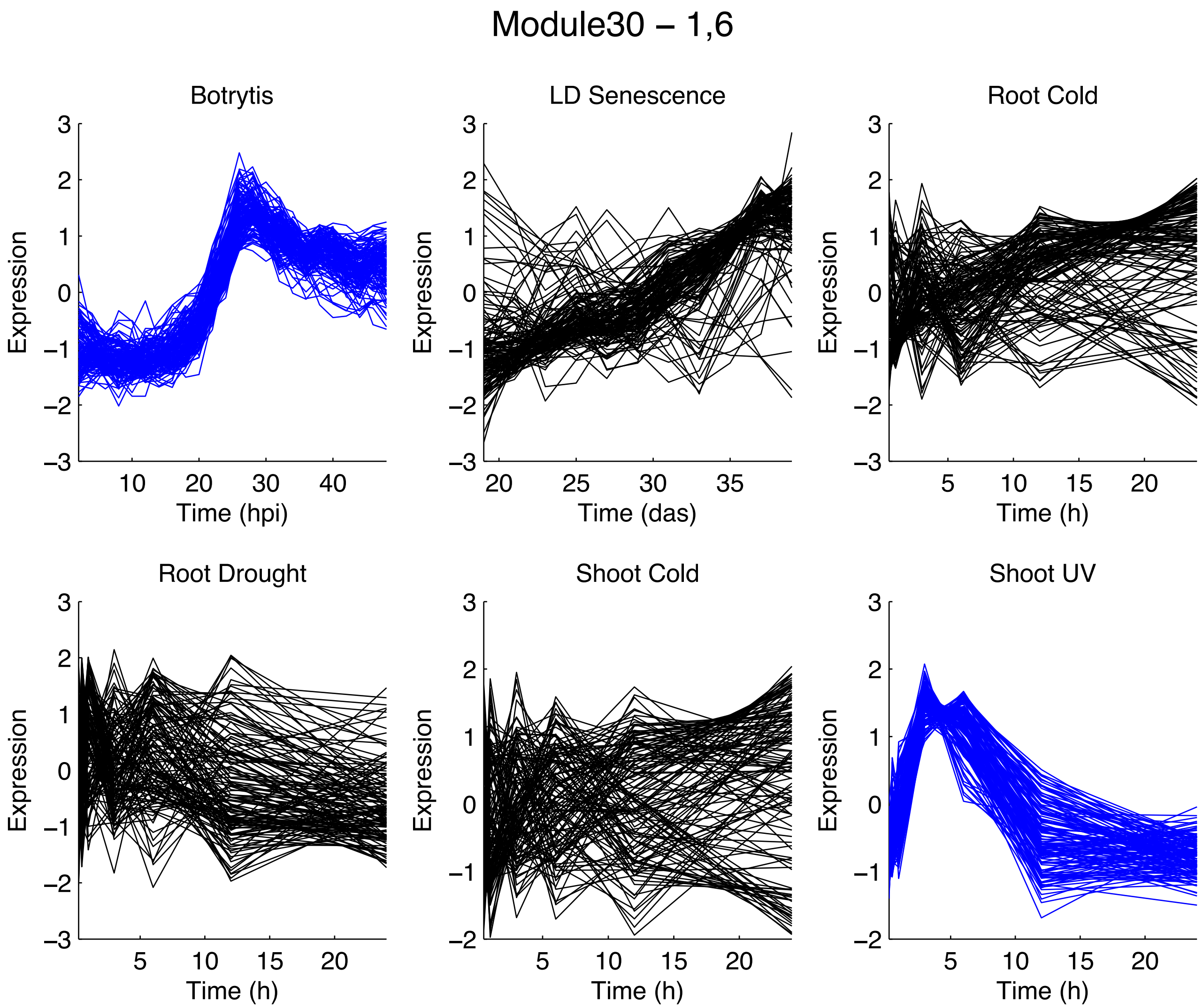
Module30 – 1,6
Botrytis
LD Senescence
Root Cold
Root Drought
Shoot Cold
Shoot UV
Expression
Time (hpi)
Time (das)
Time (h)

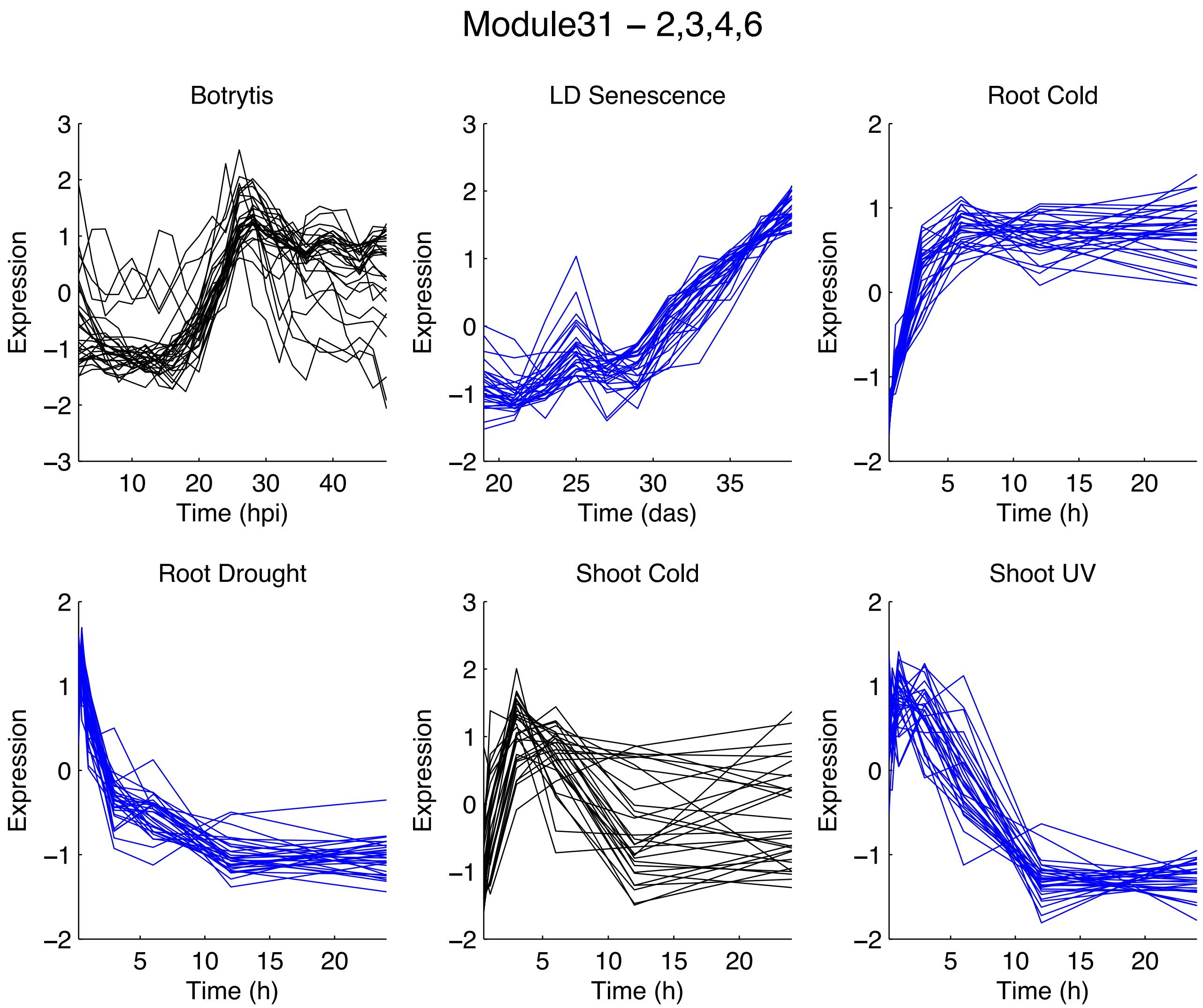
Module31 – 2,3,4,6
Botrytis
LD Senescence
Root Cold
Root Drought
Shoot Cold
Shoot UV
Expression
Time (hpi)
Time (das)
Time (h)

Module32 – 3,4,6

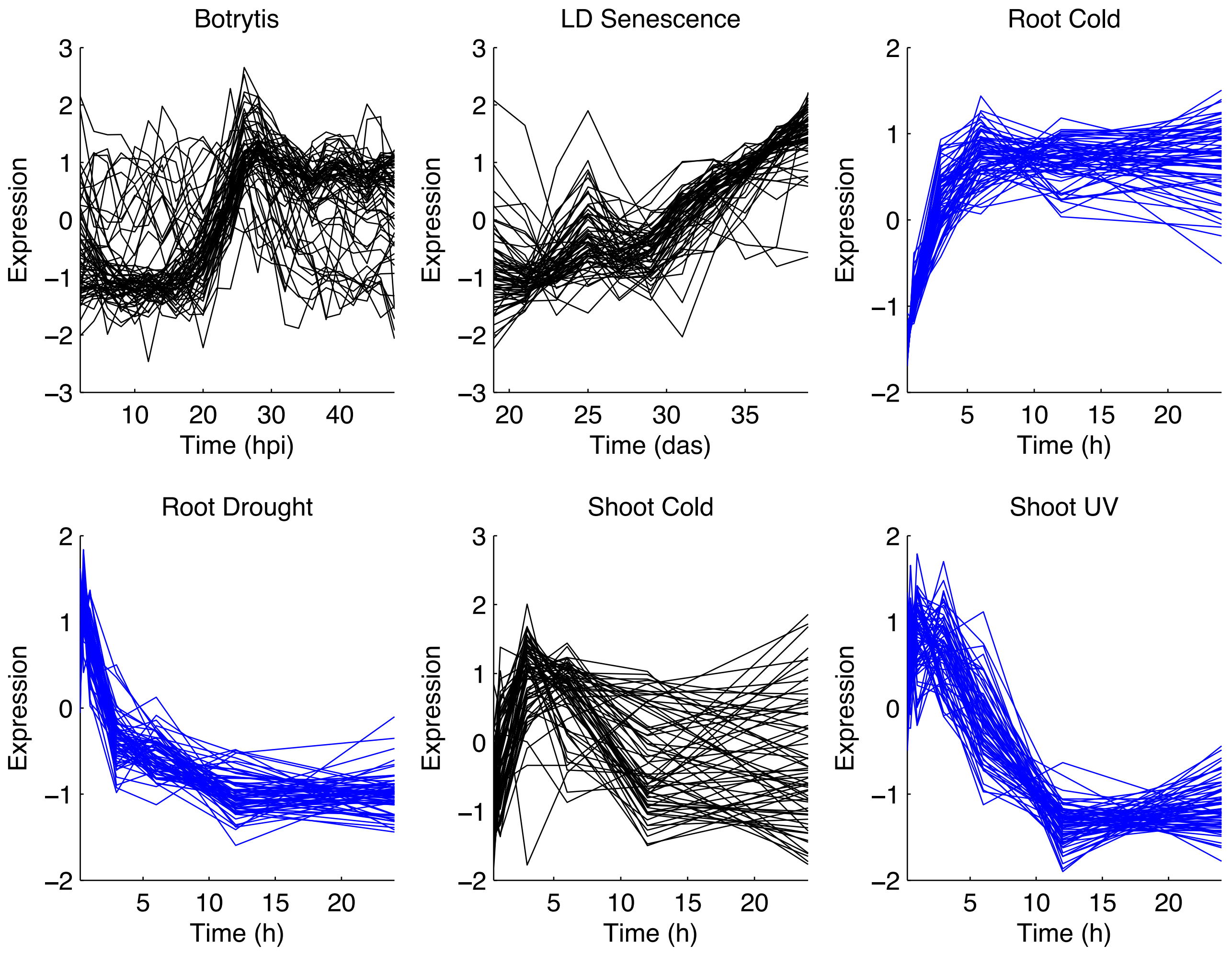

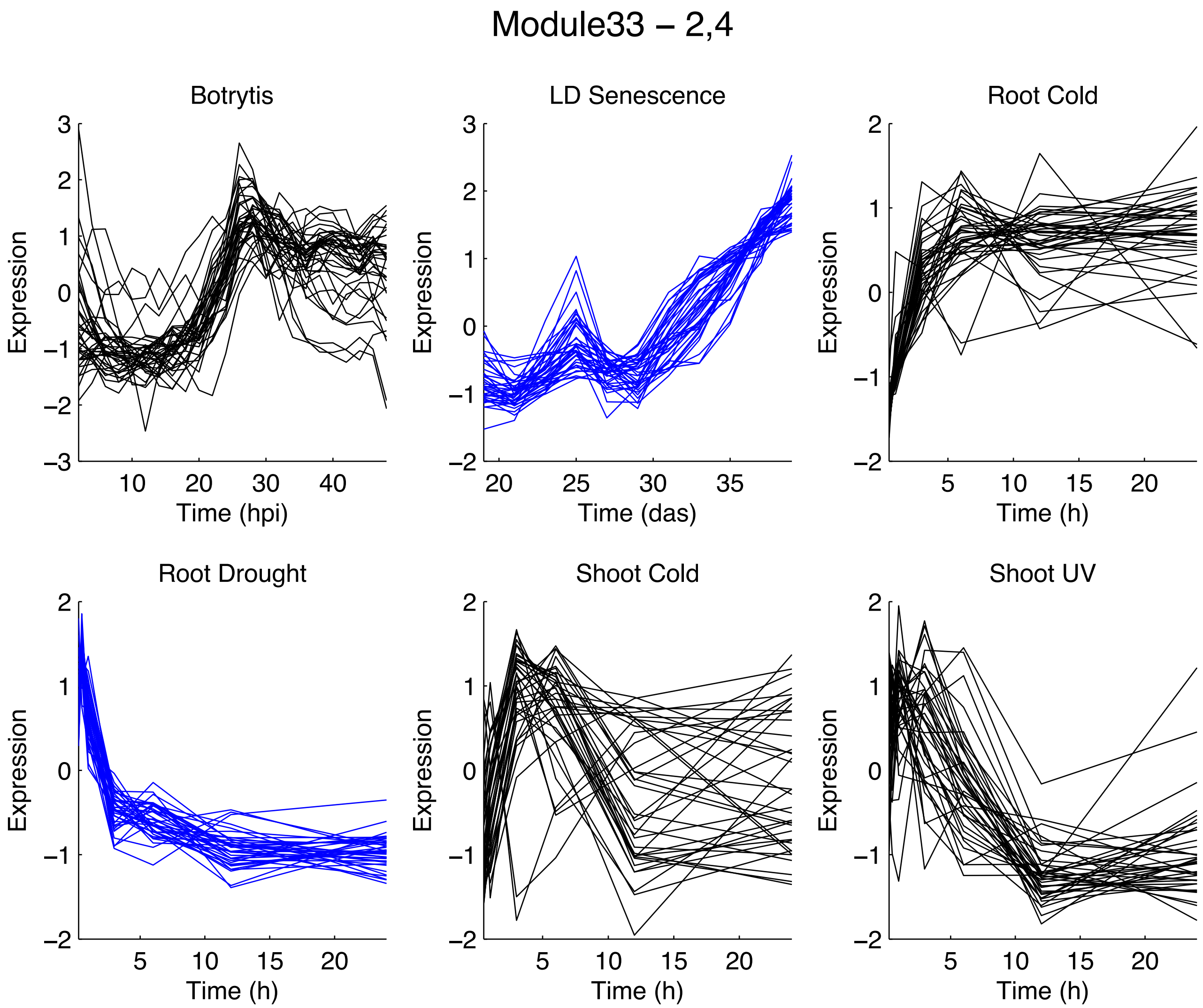

Module33 – 2,4
Botrytis
LD Senescence
Root Cold
Root Drought
Shoot Cold
Shoot UV
Expression
Time (hpi)
Time (das)
Time (h)

Module34 – 3,6

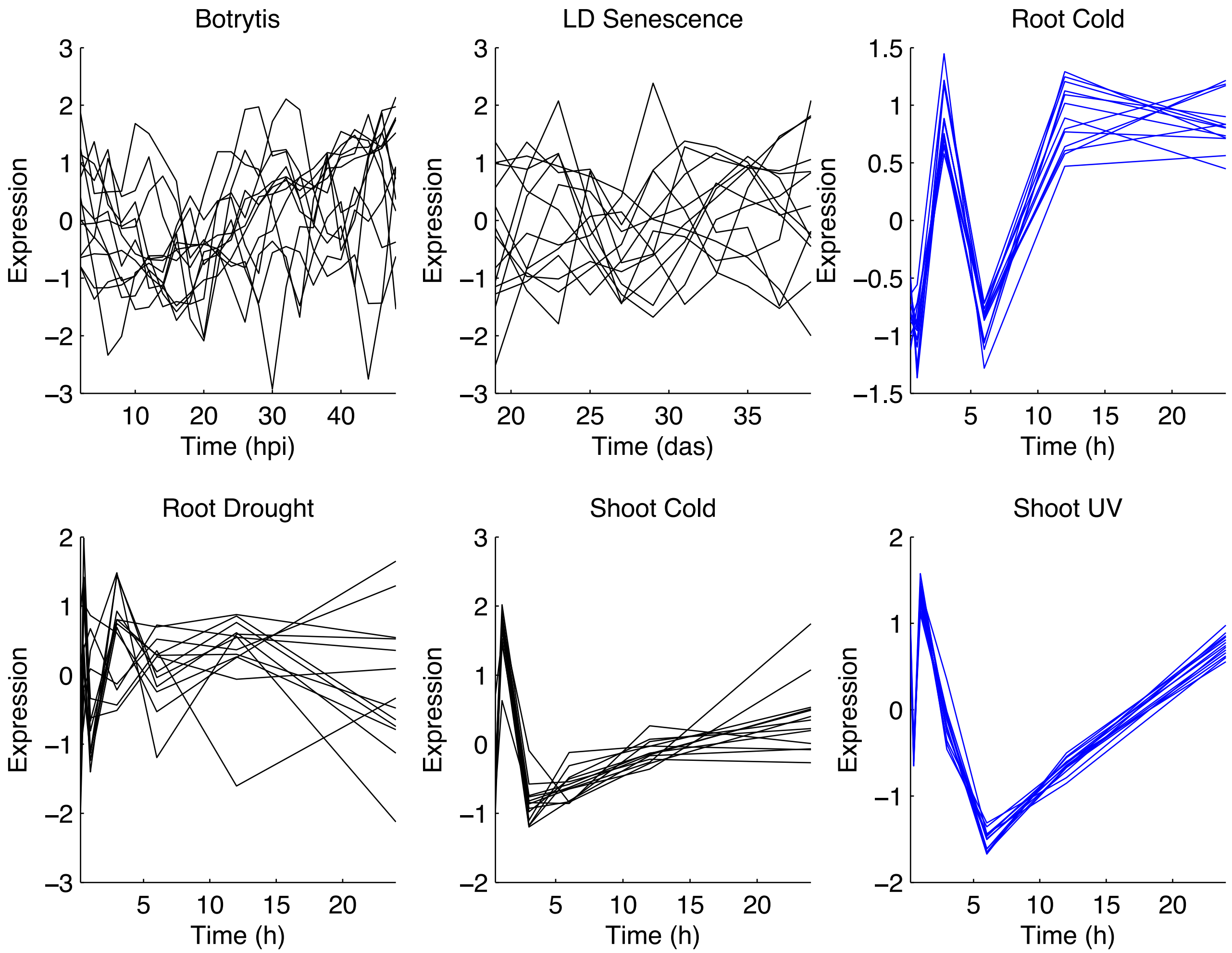
Botrytis
Expression
Time (hpi)
LD Senescence
Time (das)
Root Cold
Time (h)
Root Drought
Shoot Cold
Shoot UV

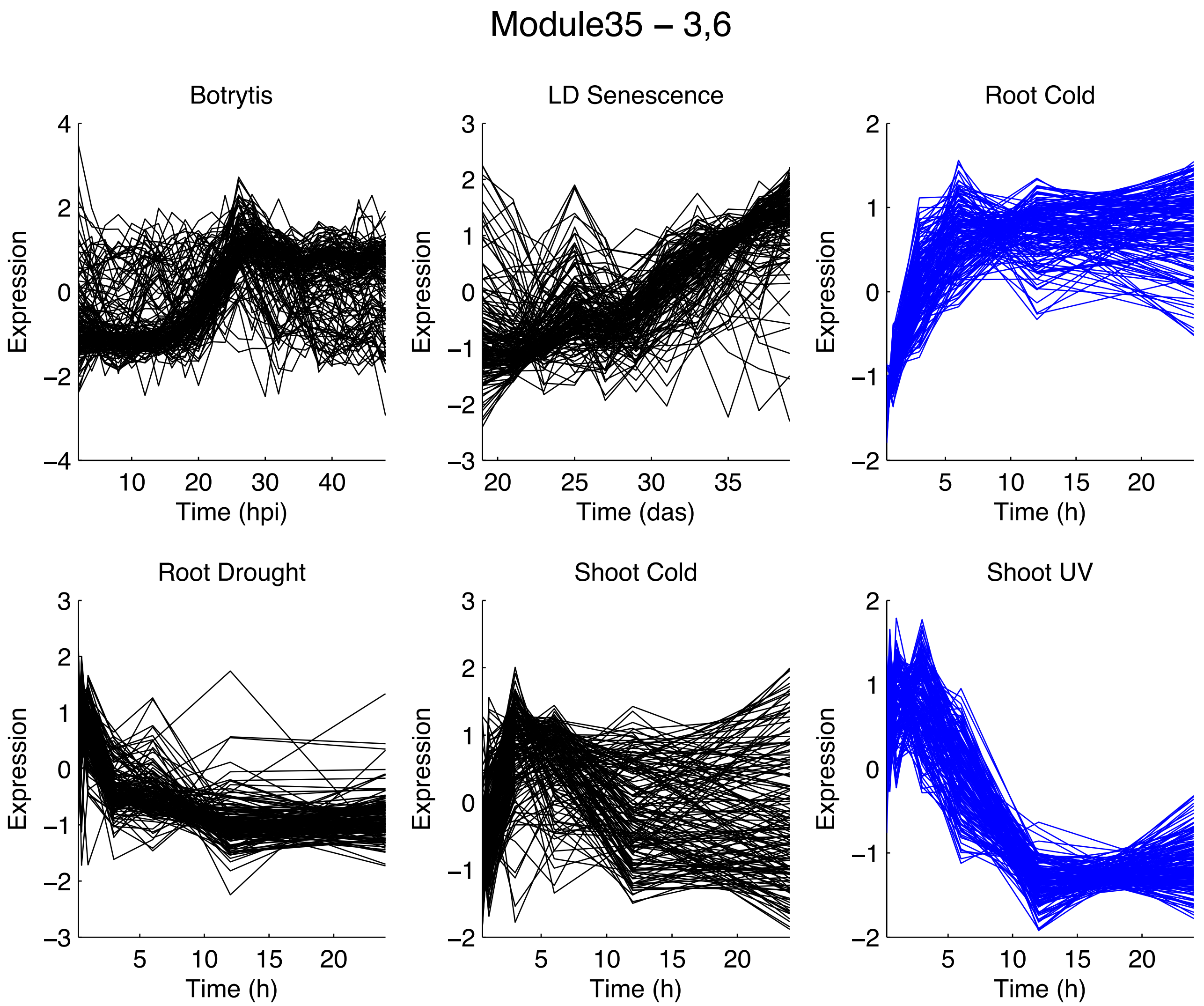
Module35 – 3,6
Botrytis
LD Senescence
Root Cold
Root Drought
Shoot Cold
Shoot UV
Expression
Time (hpi)
Time (das)
Time (h)

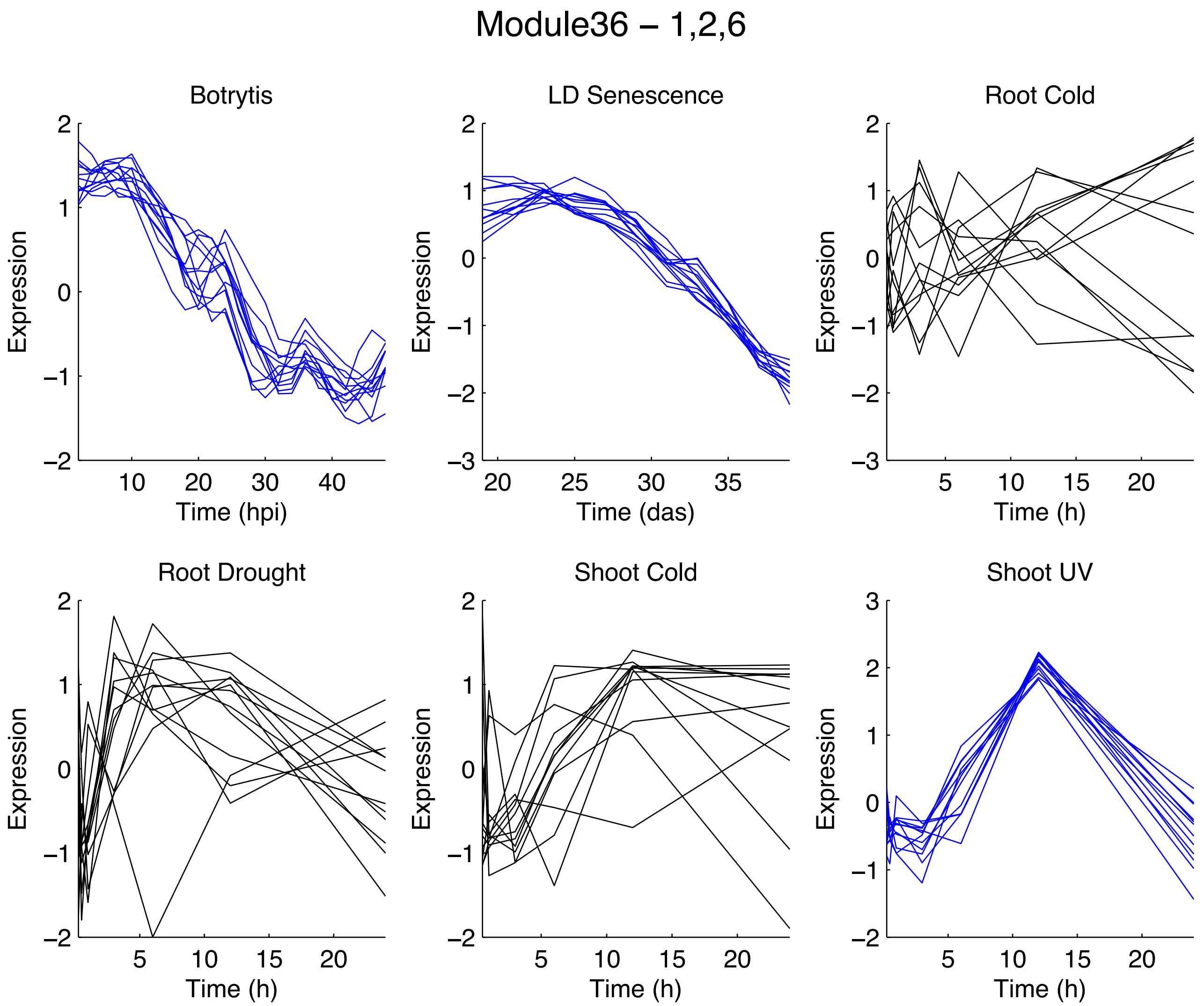

Module36 – 1,2,6
Botrytis
LD Senescence
Root Cold
Root Drought
Shoot Cold
Shoot UV
Expression
Time (hpi)
Time (das)
Time (h)

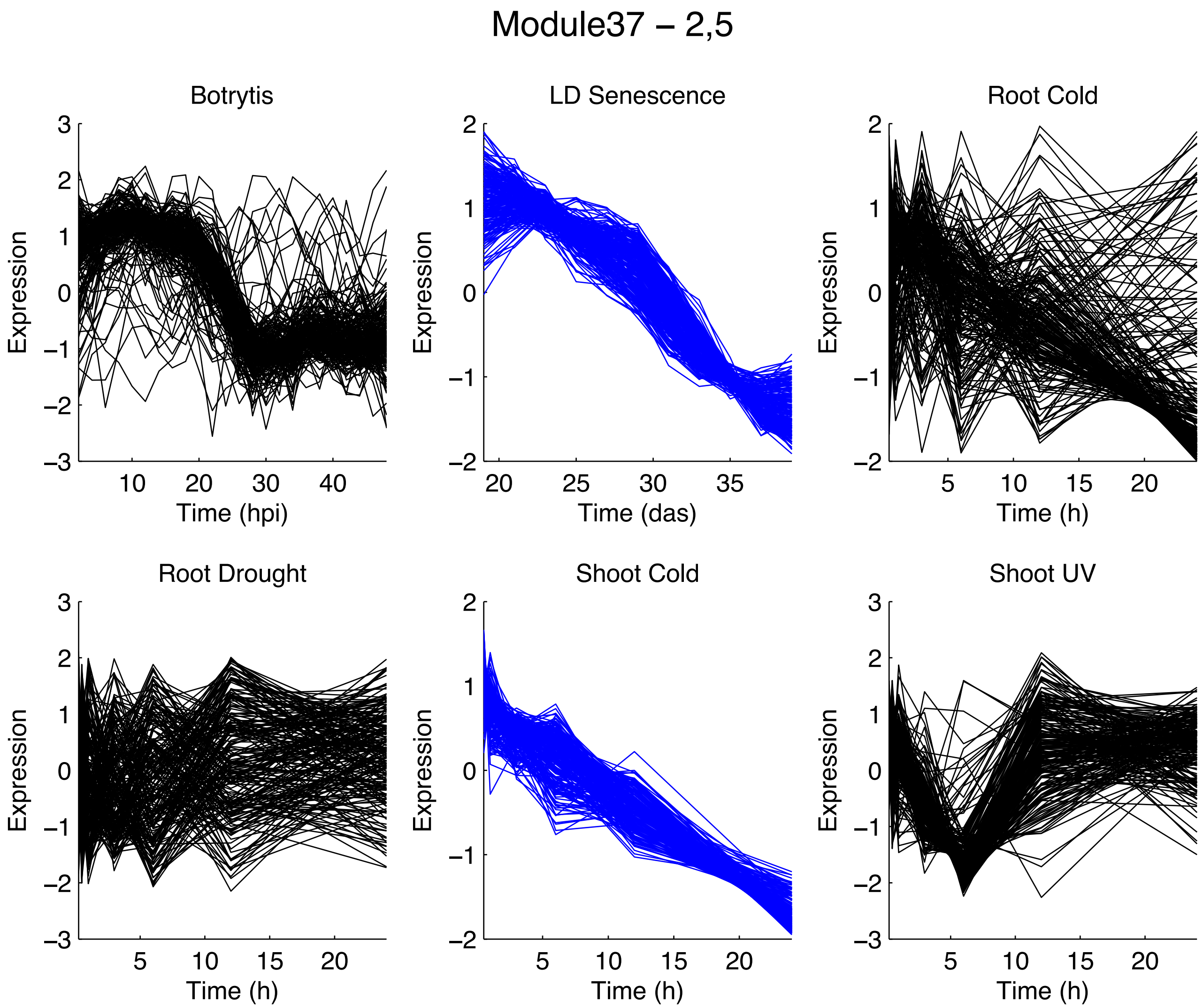
Module37 – 2,5
Botrytis
LD Senescence
Root Cold
Root Drought
Shoot Cold
Shoot UV
Expression
Time (hpi)
Time (das)
Time (h)

Module38 – 1,5,6

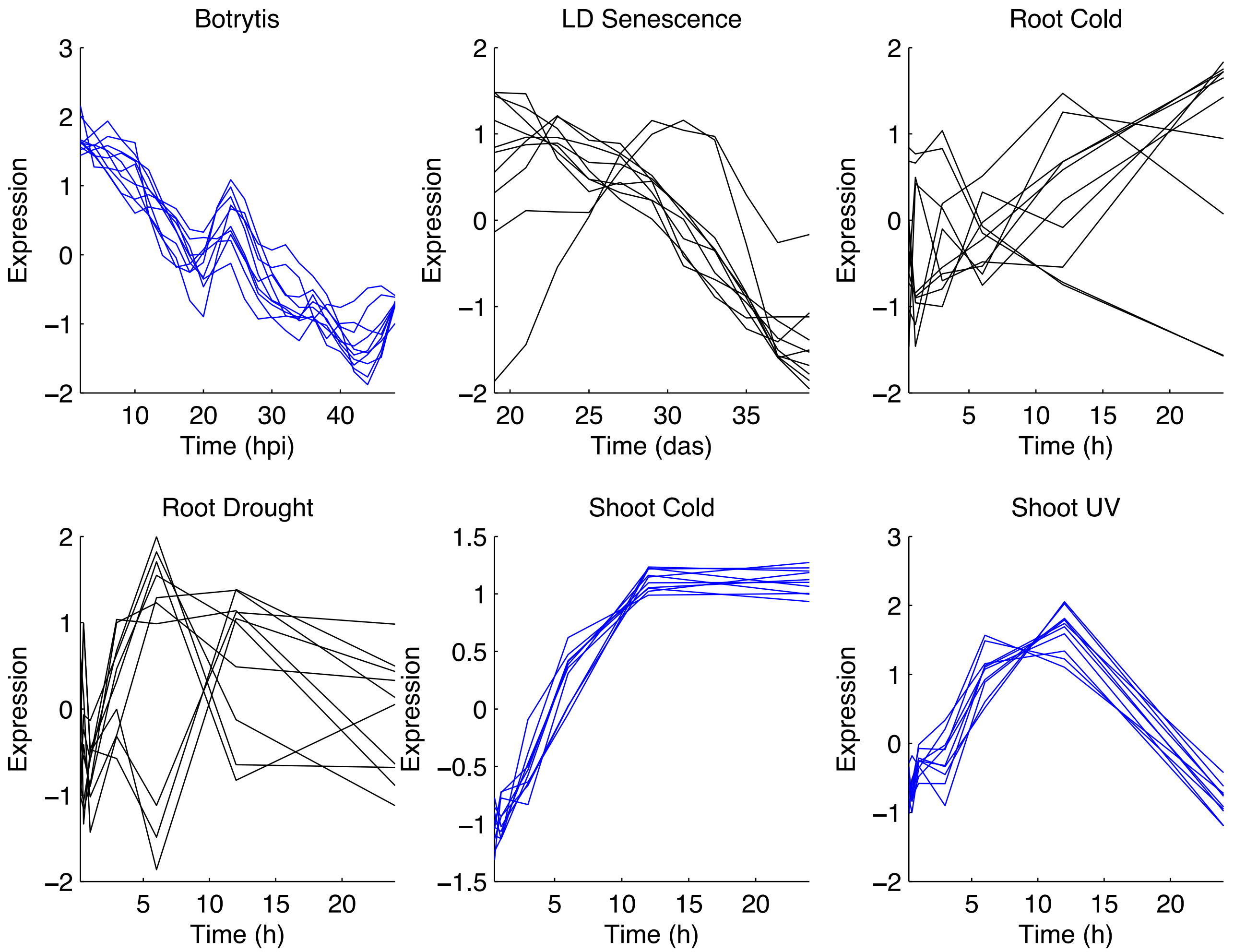

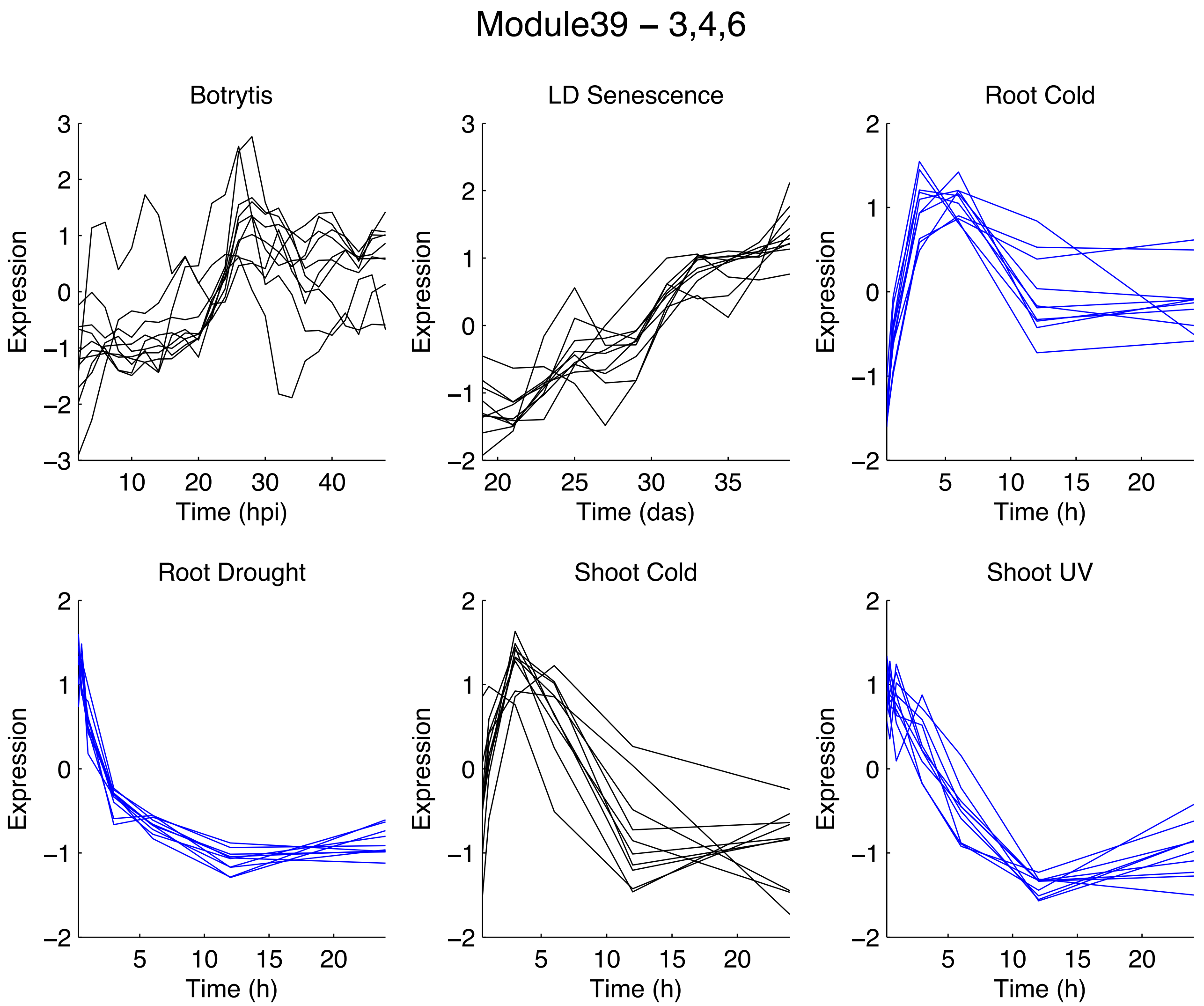
Module39 – 3,4,6
Botrytis
LD Senescence
Root Cold
Root Drought
Shoot Cold
Shoot UV
Expression
Time (hpi)
Time (das)
Time (h)

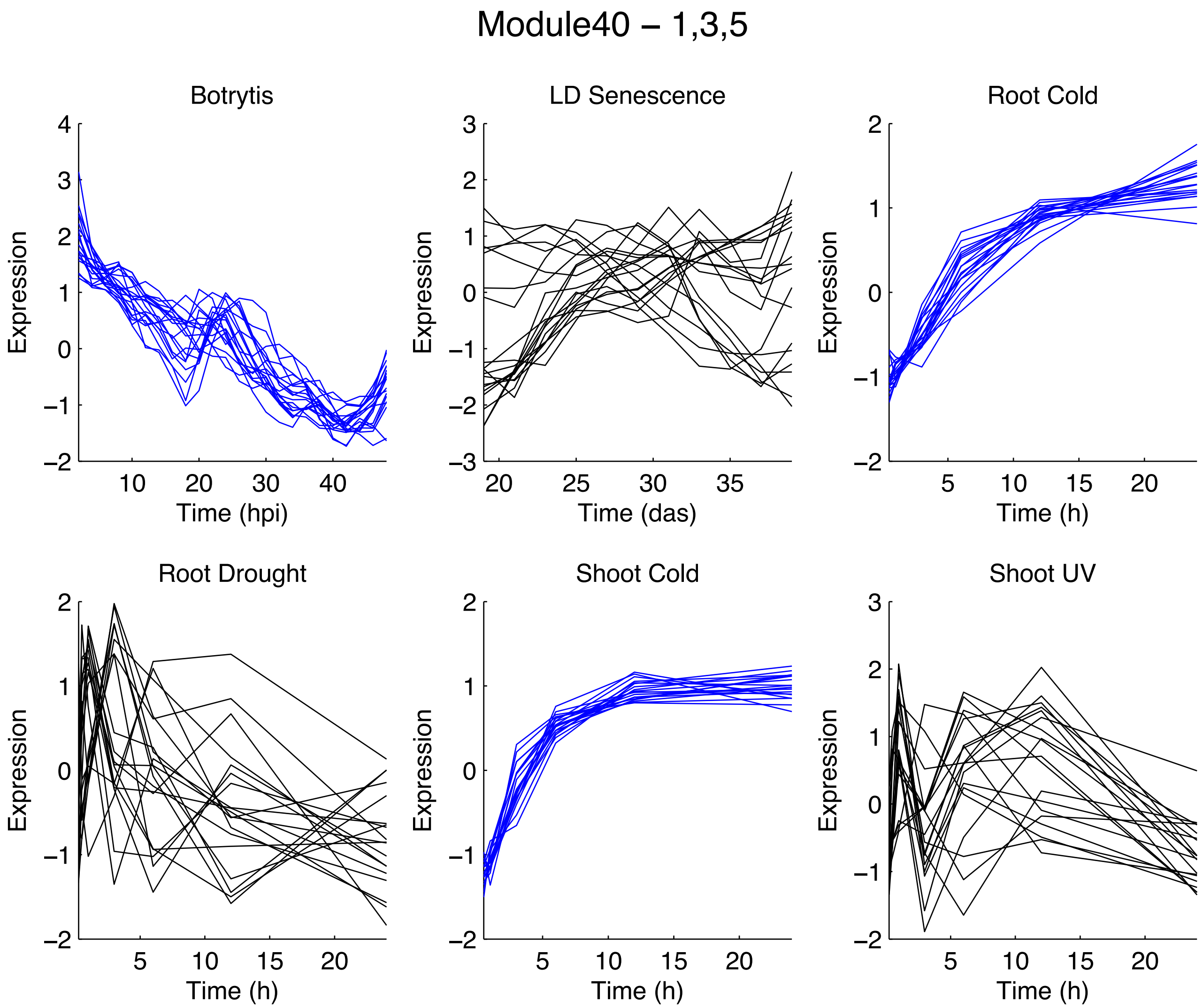
Module40 – 1,3,5
Botrytis
LD Senescence
Root Cold
Root Drought
Shoot Cold
Shoot UV
Expression
Time (hpi)
Time (das)
Time (h)

Module41 – 5,6

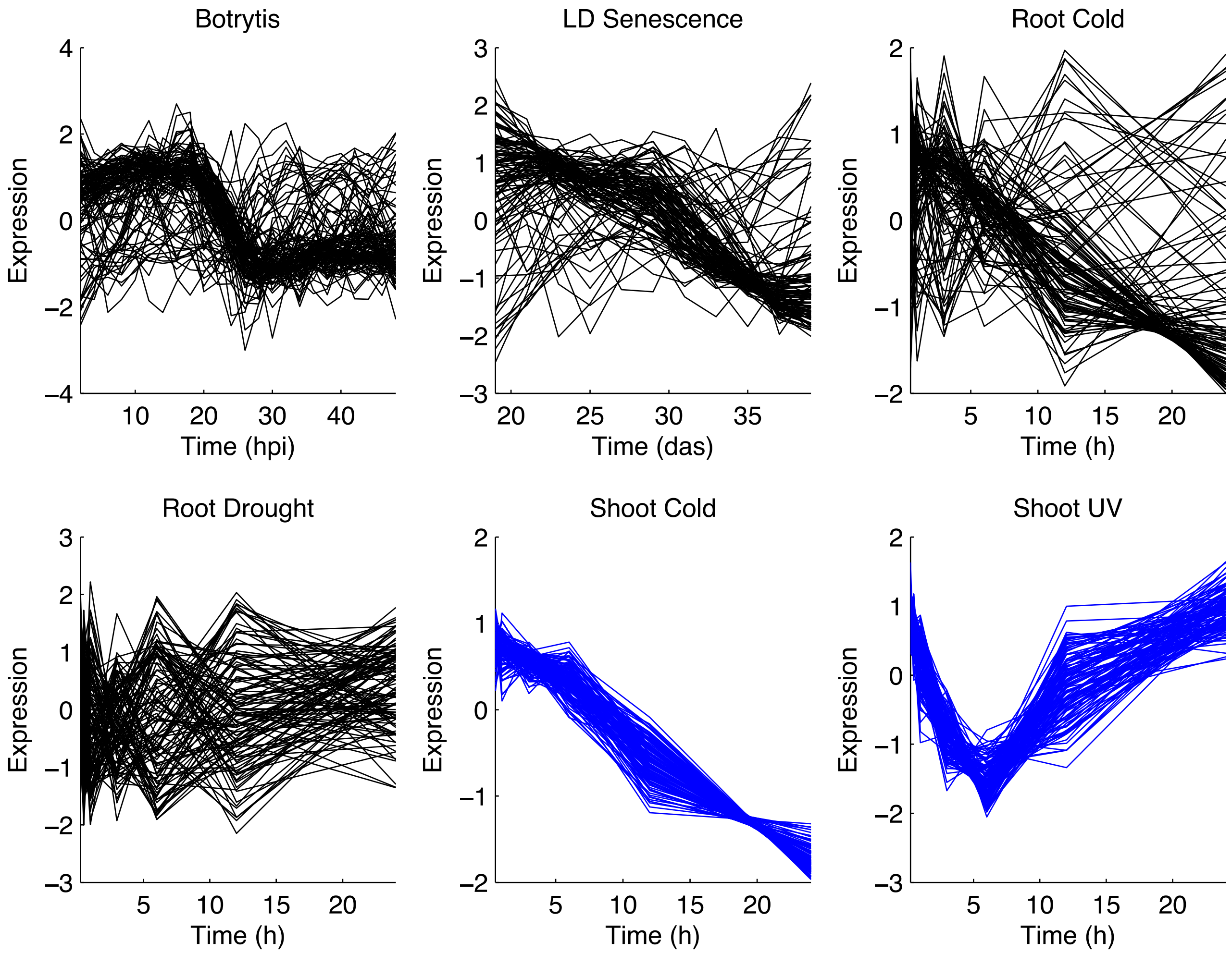

Botrytis
Expression
Time (hpi)
LD Senescence
Expression
Time (das)
Root Cold
Expression
Time (h)
Root Drought
Expression
Time (h)
Shoot Cold
Expression
Time (h)
Shoot UV
Expression
Time (h)

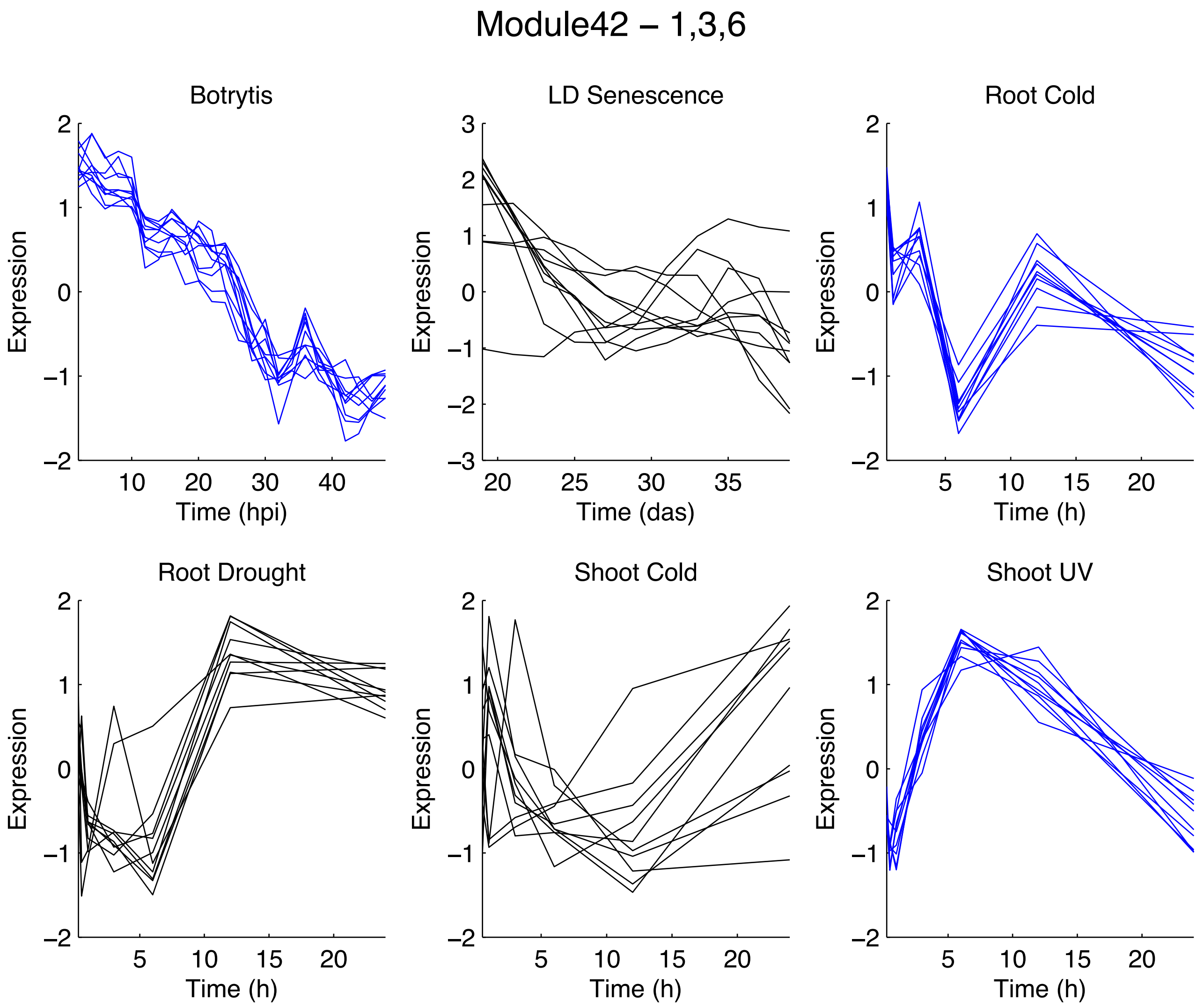
Module42 – 1,3,6
Botrytis
LD Senescence
Root Cold
Root Drought
Shoot Cold
Shoot UV
Expression
Time (hpi)
Time (das)
Time (h)

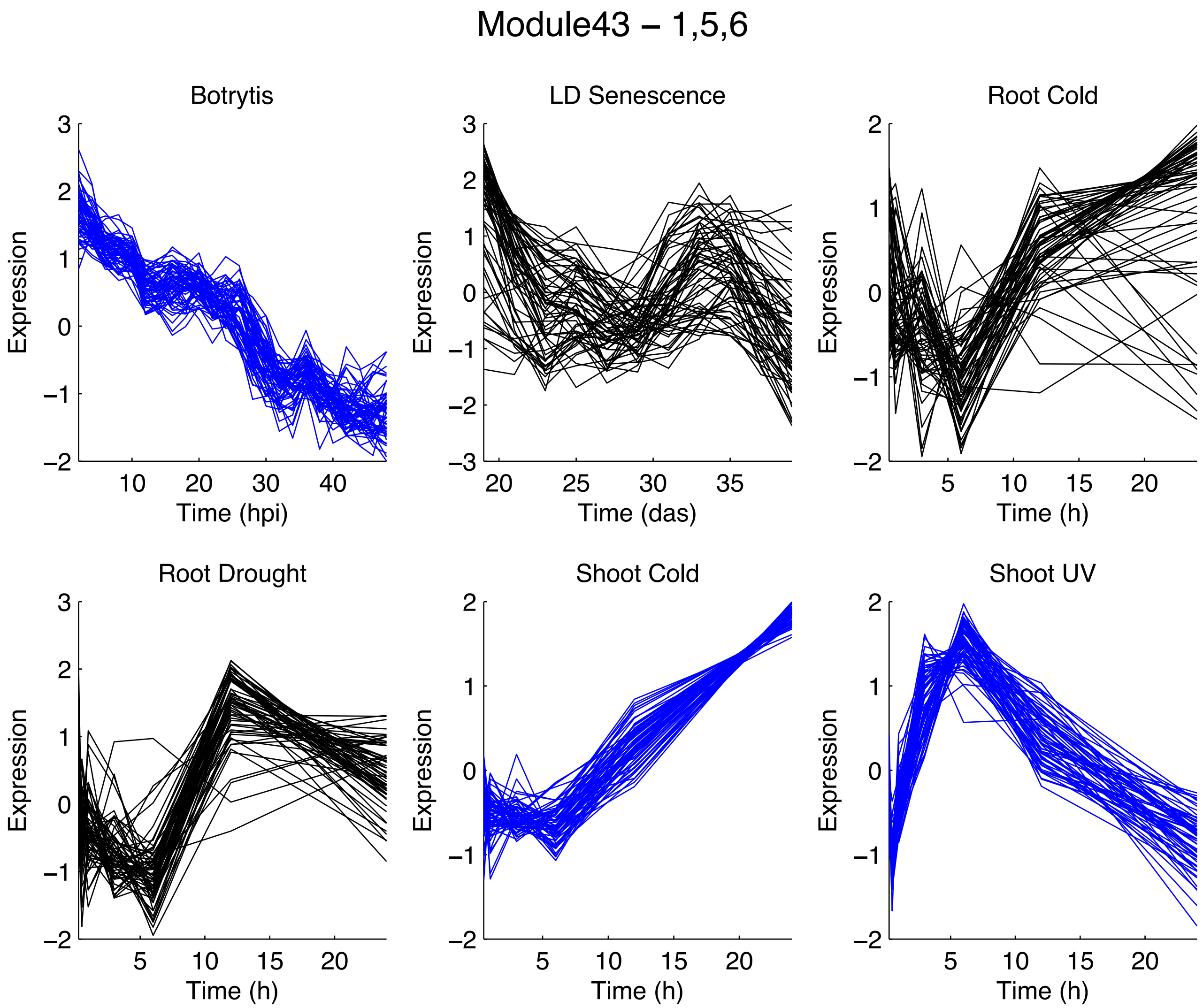
Module43 – 1,5,6
Botrytis
LD Senescence
Root Cold
Root Drought
Shoot Cold
Shoot UV
Expression
Time (hpi)
Time (das)
Time (h)

Module44 – 1,3,5,6

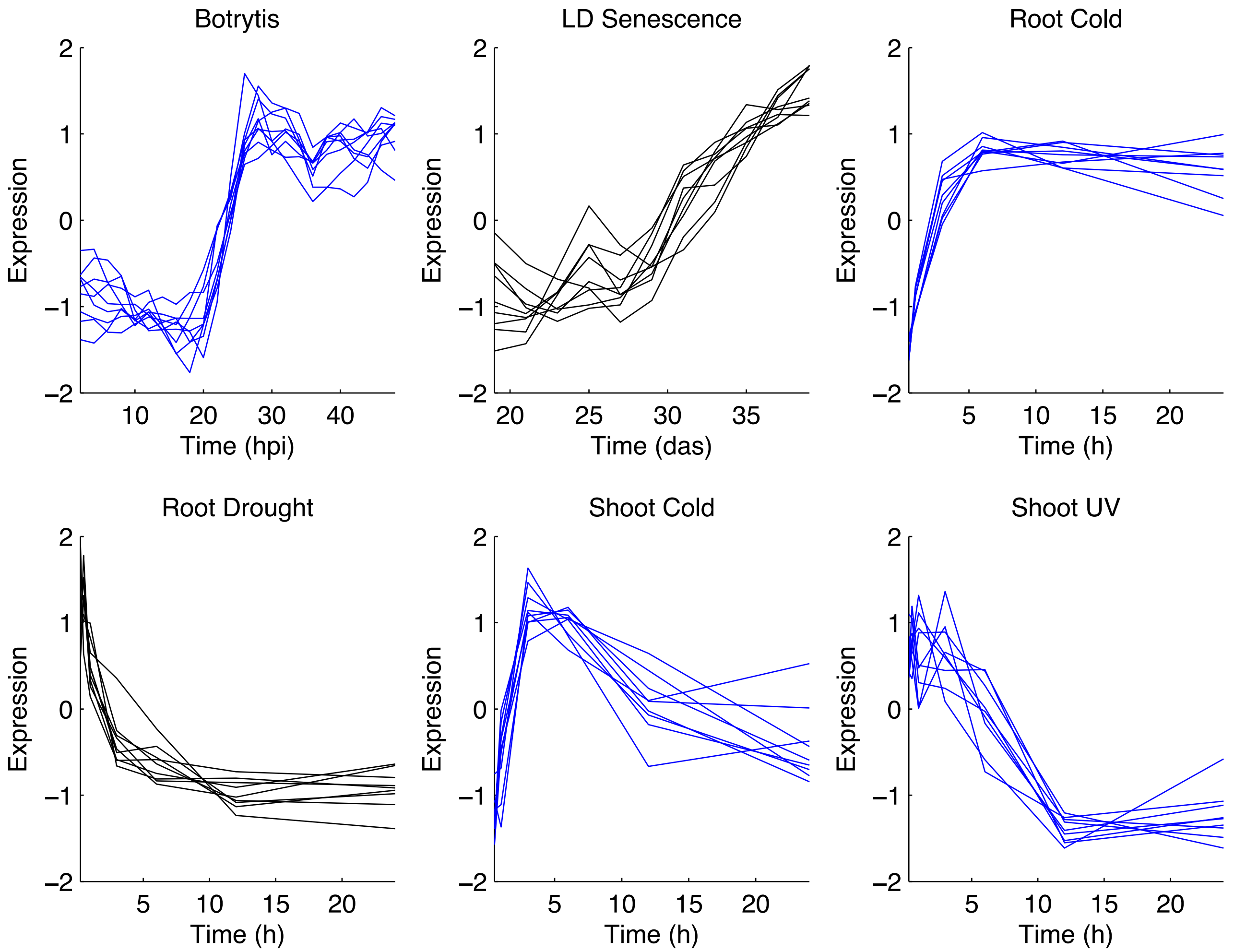

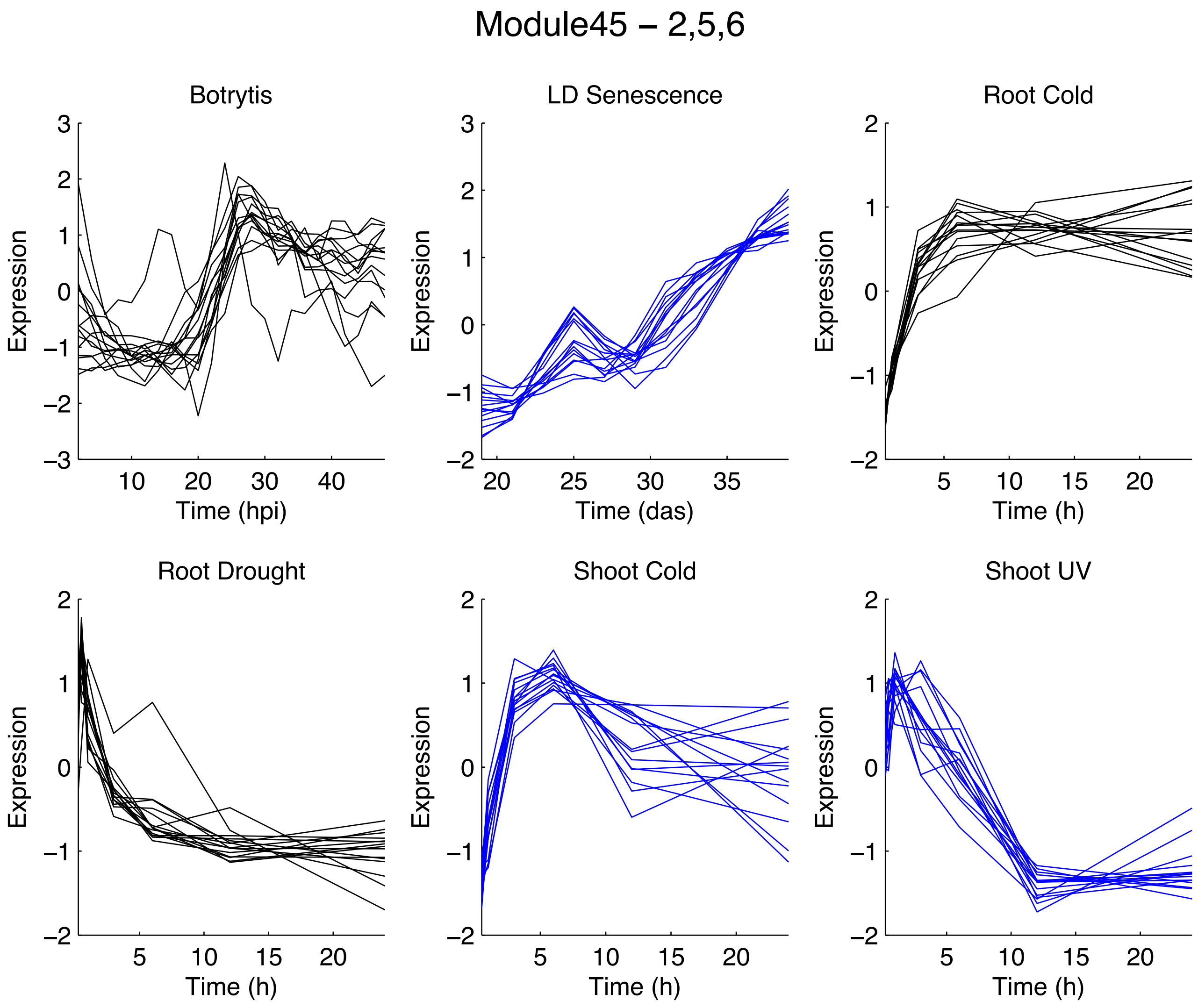

Module45 – 2,5,6
Botrytis
LD Senescence
Root Cold
Root Drought
Shoot Cold
Shoot UV
Expression
Time (hpi)
Time (das)
Time (h)

Module46 – 3,5

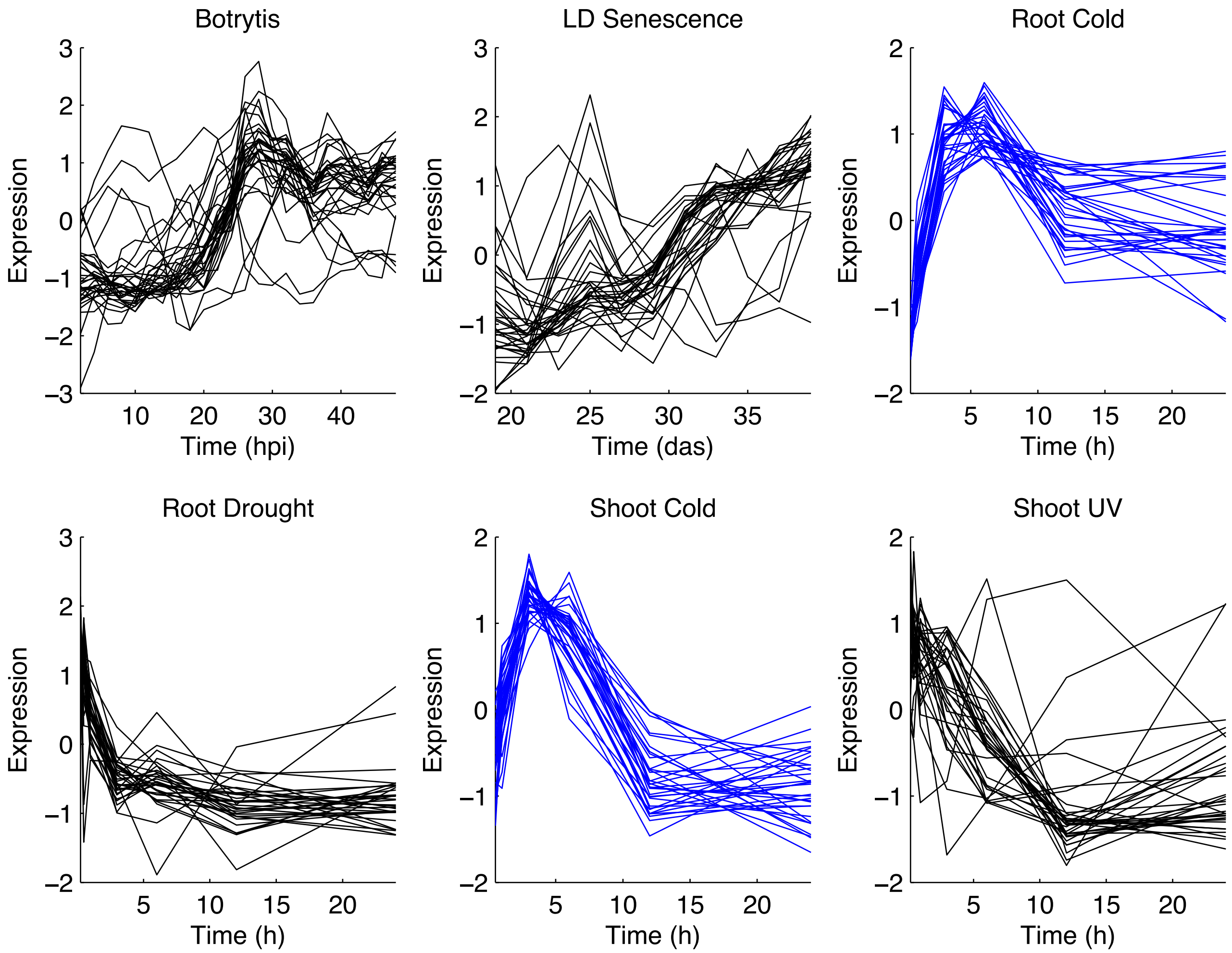

Botrytis
LD Senescence
Root Cold
Root Drought
Shoot Cold
Shoot UV
Expression
Time (hpi)
Time (das)
Time (h)
3
2
1
0
−1
−2
−3
10
20
30
40
25
35
5
15

Module47 – 1,3,5

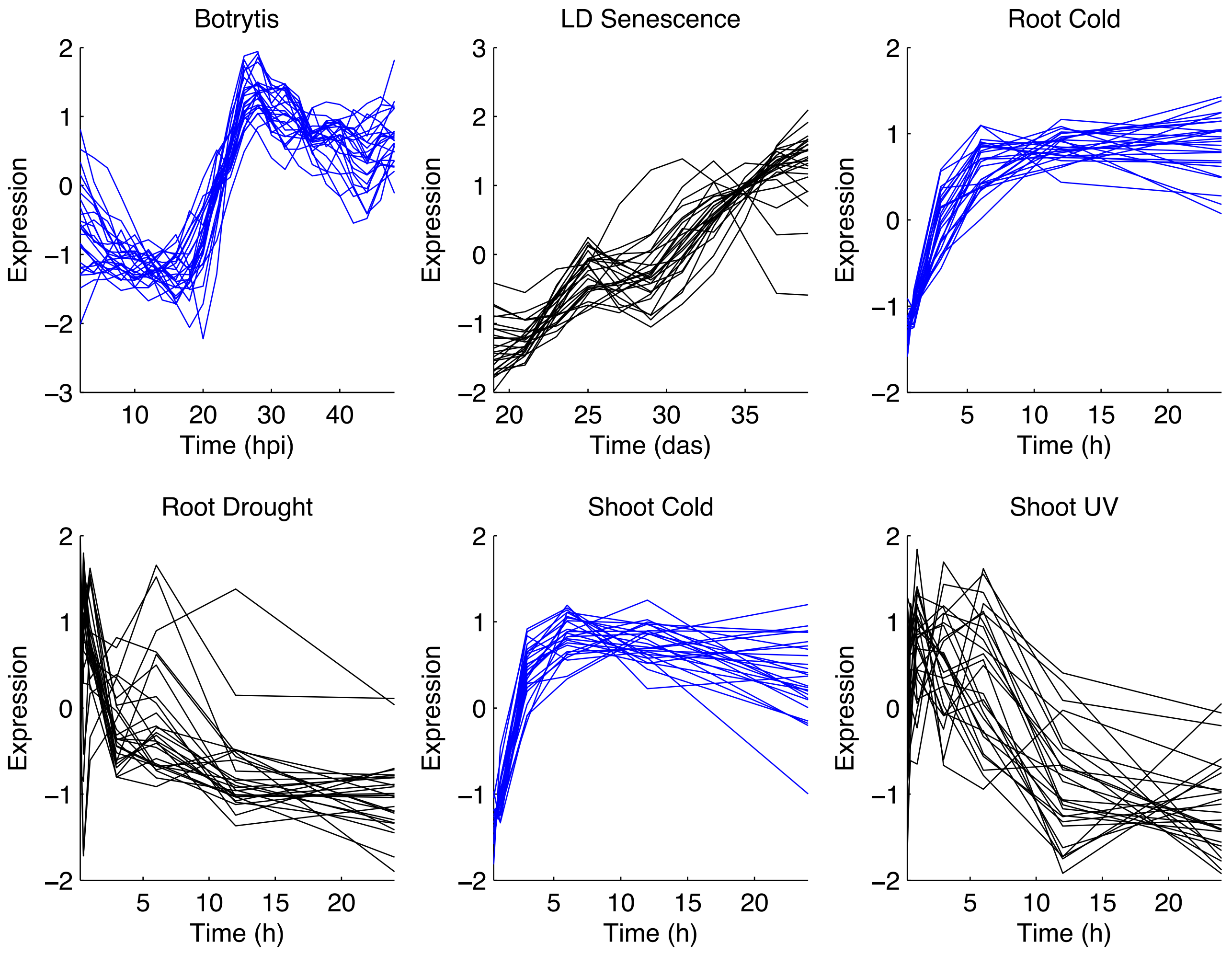

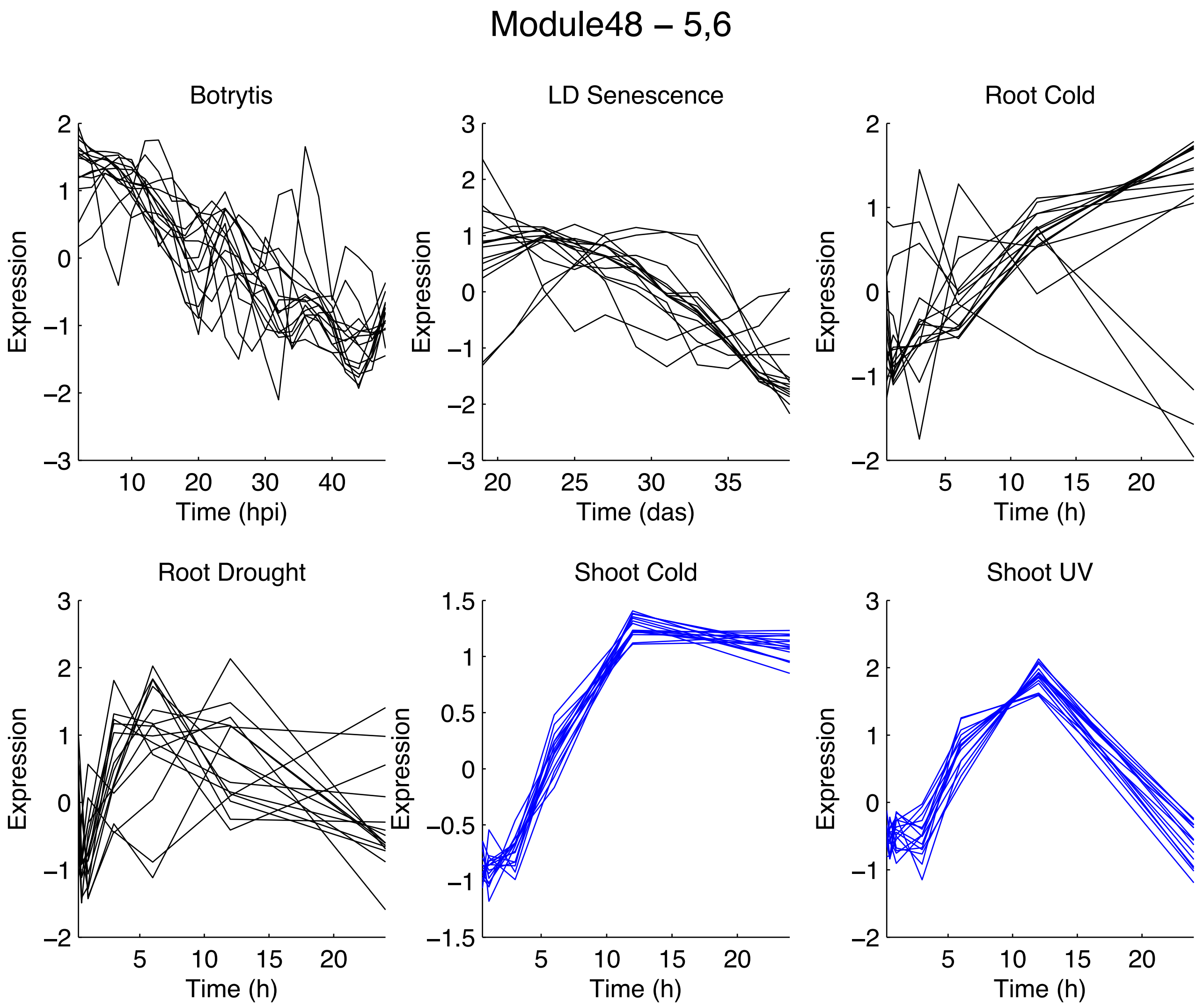
Module48 – 5,6
Botrytis
LD Senescence
Root Cold
Root Drought
Shoot Cold
Shoot UV
Expression
Time (hpi)
Time (das)
Time (h)

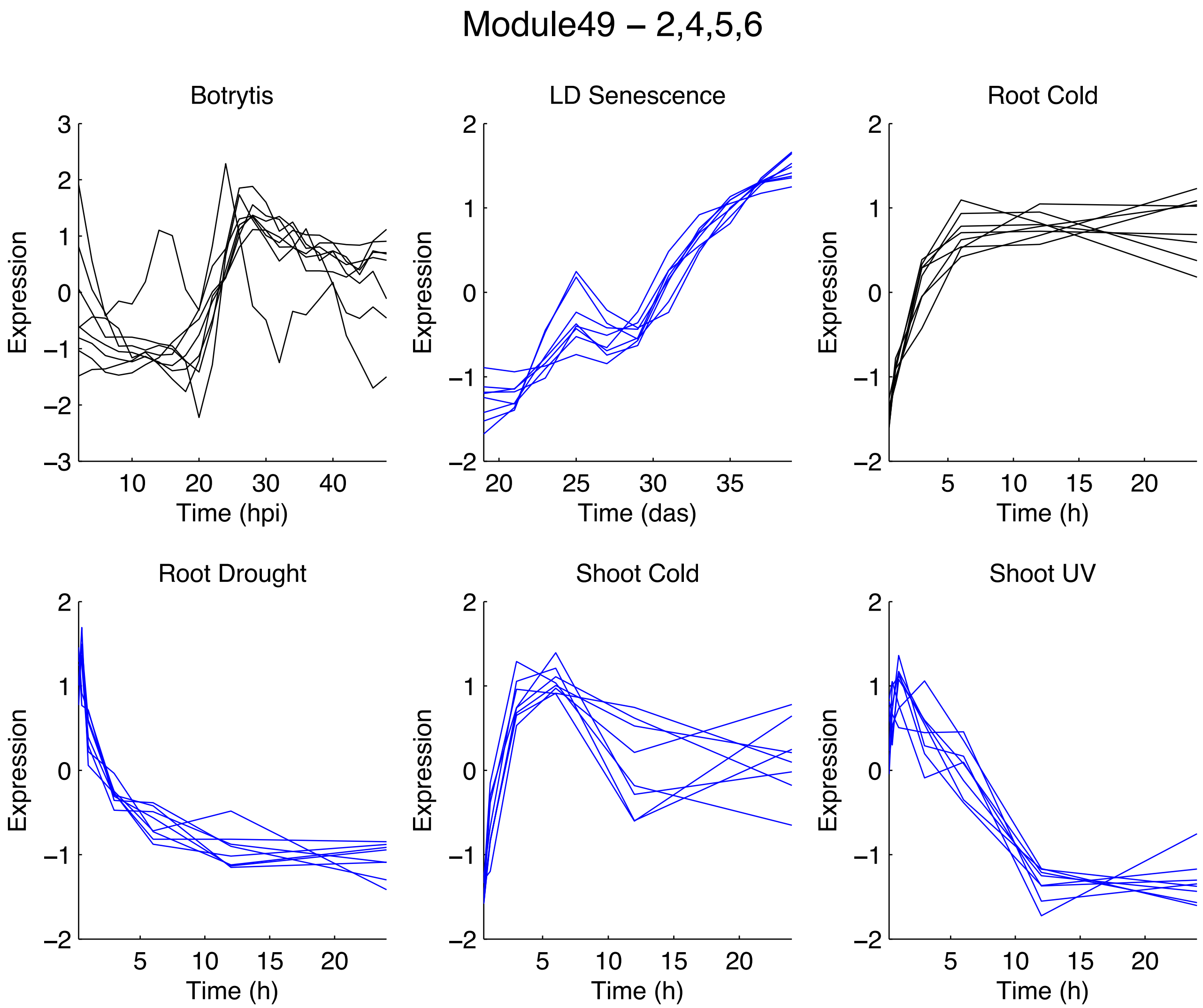
Module49 – 2,4,5,6
Botrytis
LD Senescence
Root Cold
Root Drought
Shoot Cold
Shoot UV
Expression
Time (hpi)
Time (das)
Time (h)

Module50 – 2,4,5

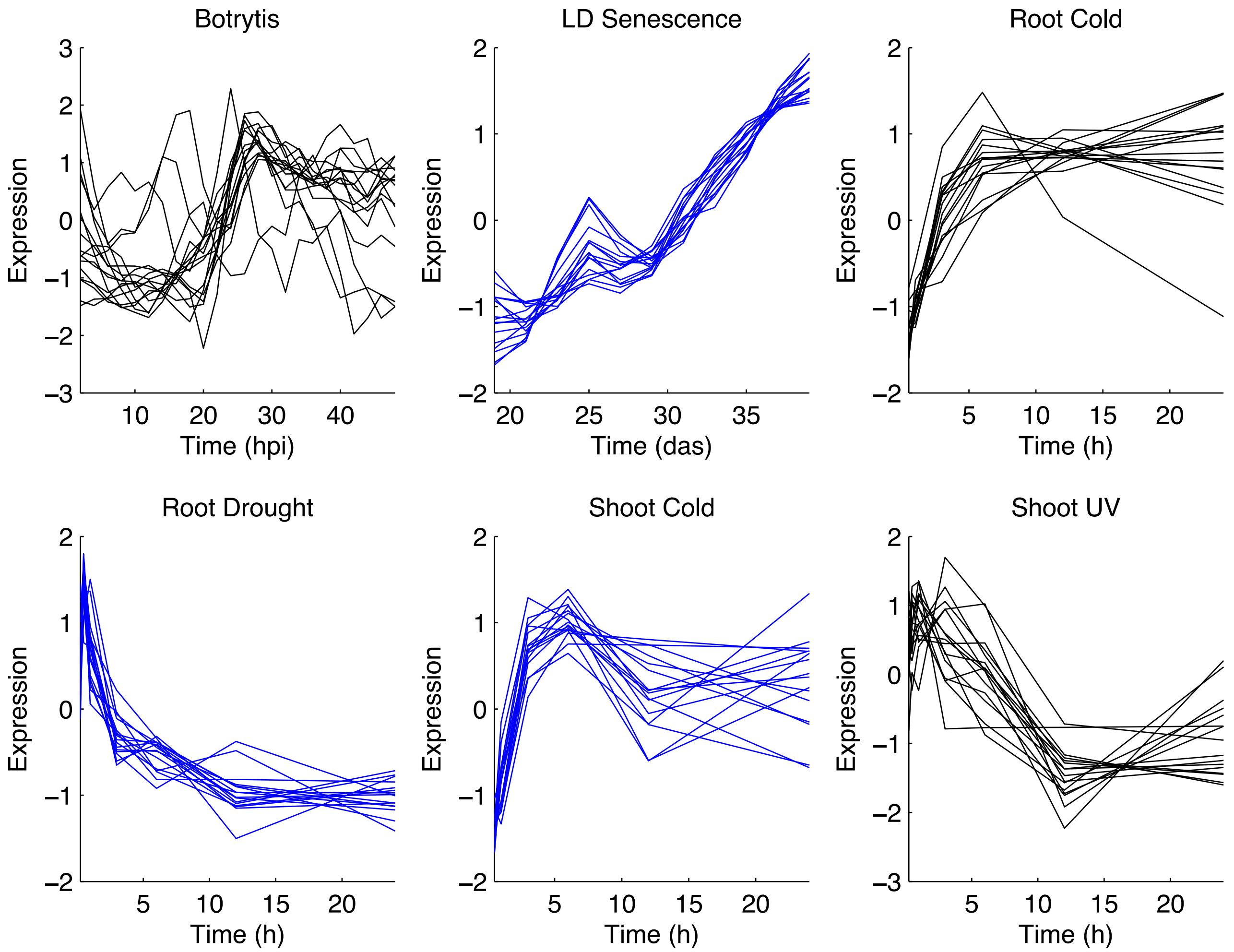

Module51 – 1,2,5

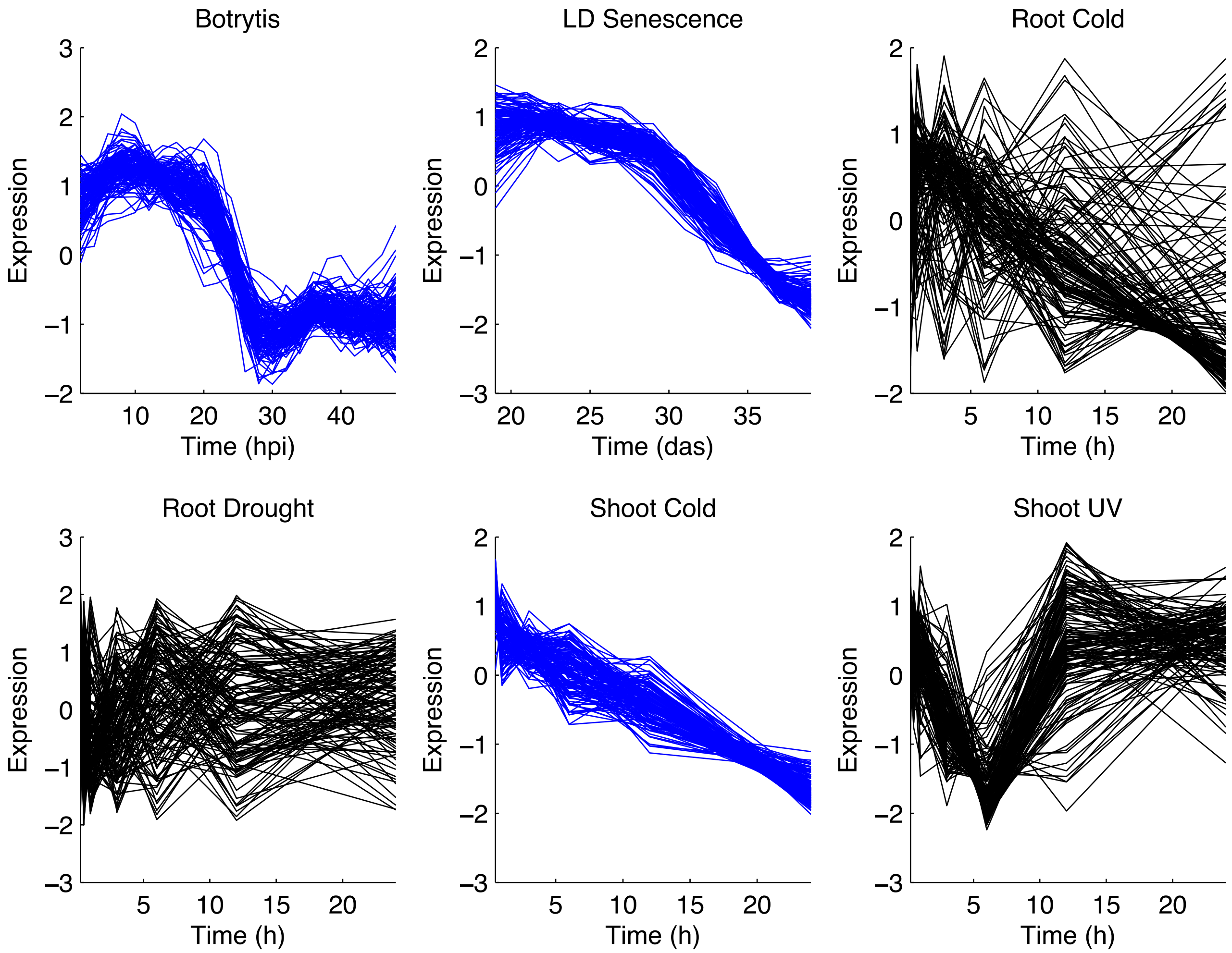

Module52 – 3,4

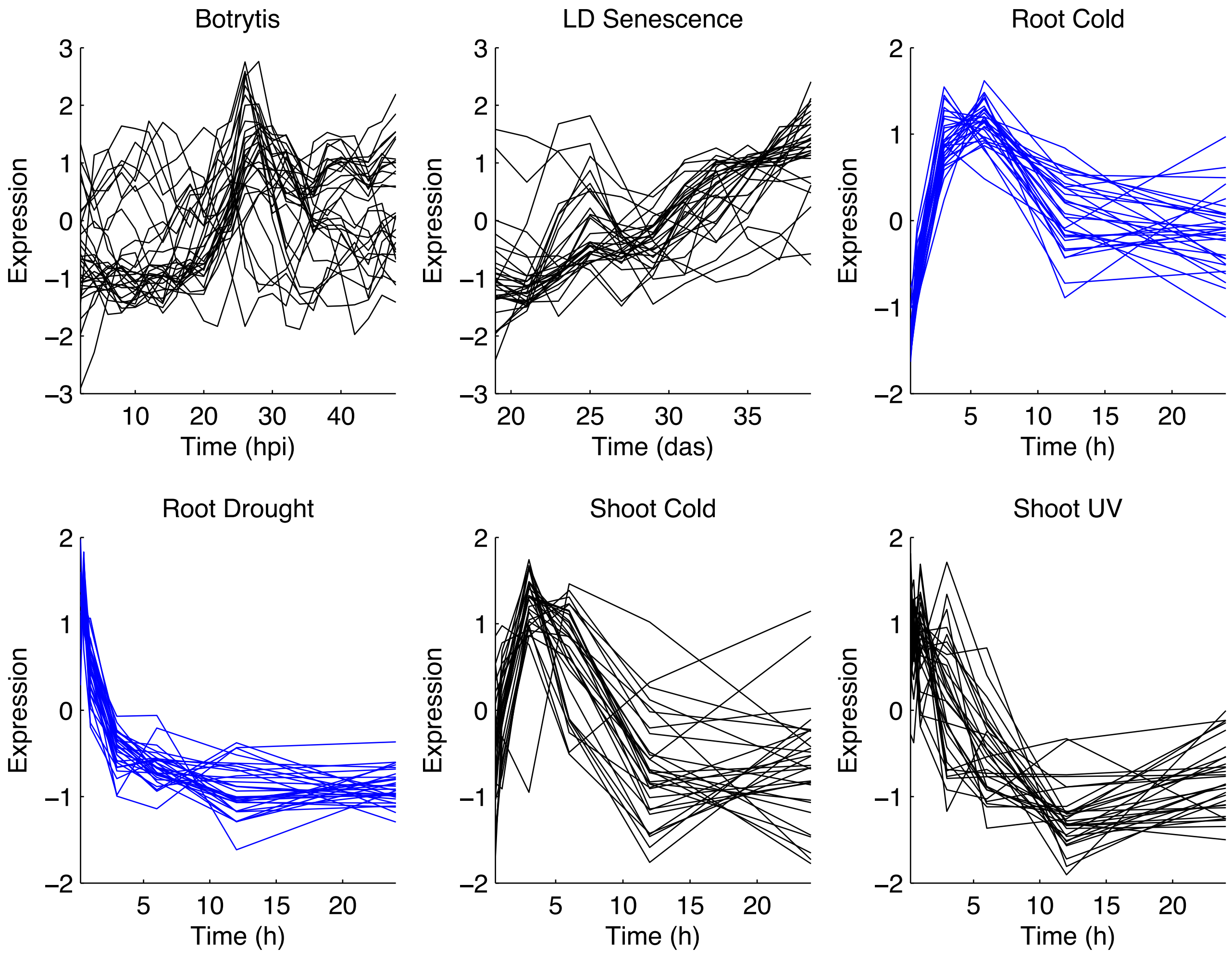

Botrytis
LD Senescence
Root Cold
Root Drought
Shoot Cold
Shoot UV
Expression
Time (hpi)
Time (das)
Time (h)

Module53 – 1,2,6

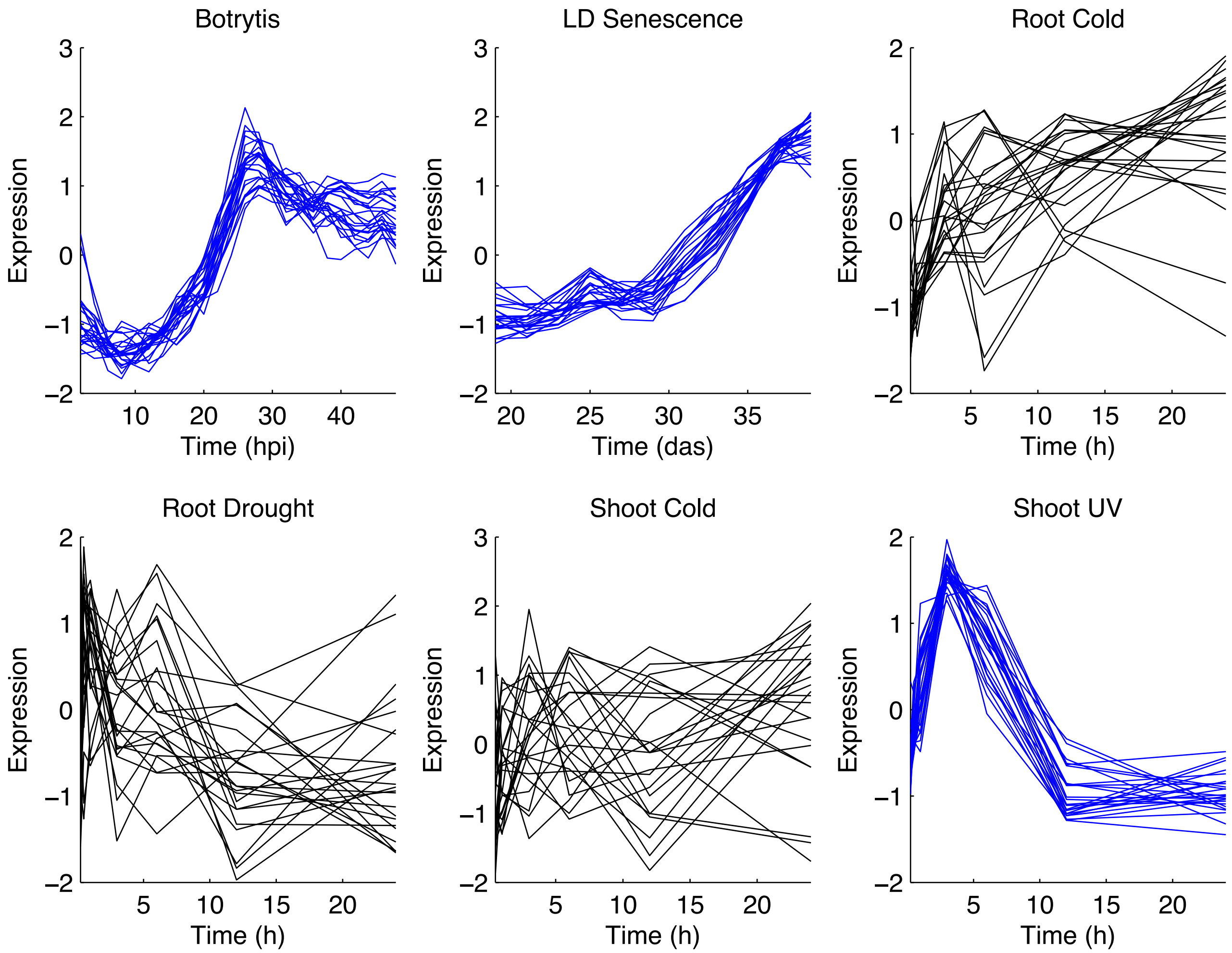

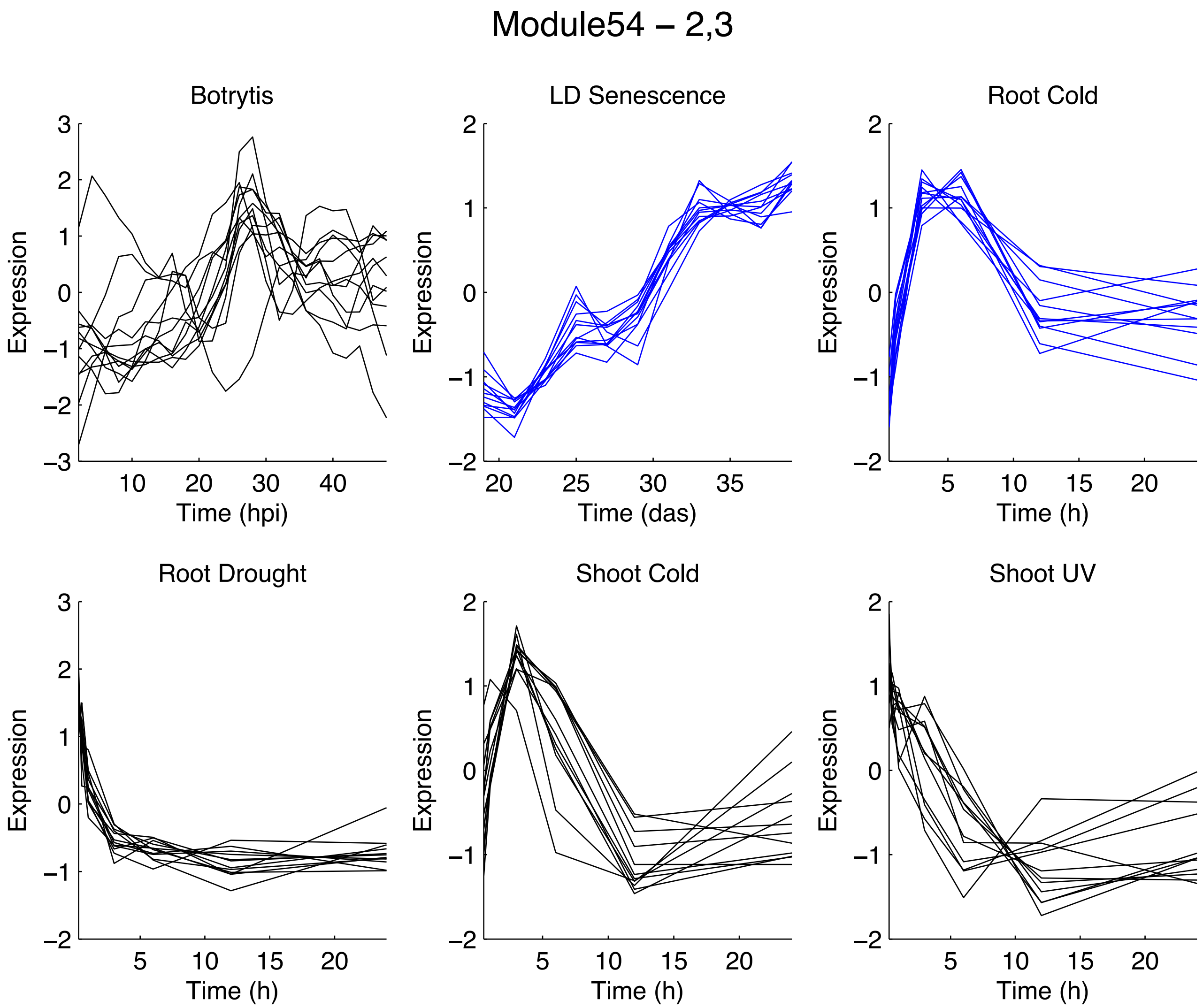

Module54 – 2,3
Botrytis
LD Senescence
Root Cold
Root Drought
Shoot Cold
Shoot UV
Expression
Time (hpi)
Time (das)
Time (h)

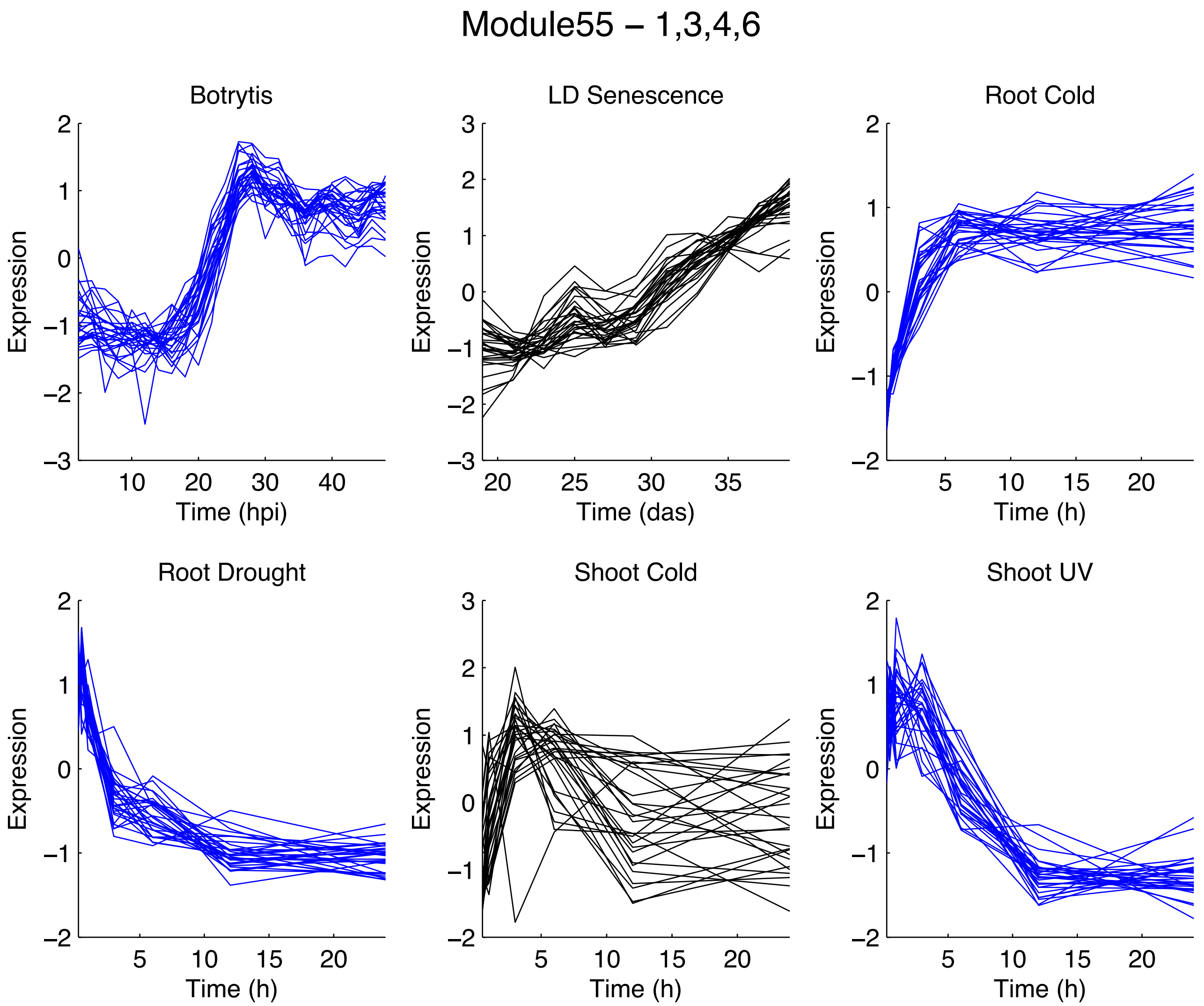
Module55 – 1,3,4,6
Botrytis
LD Senescence
Root Cold
Root Drought
Shoot Cold
Shoot UV
Expression
Time (hpi)
Time (das)
Time (h)

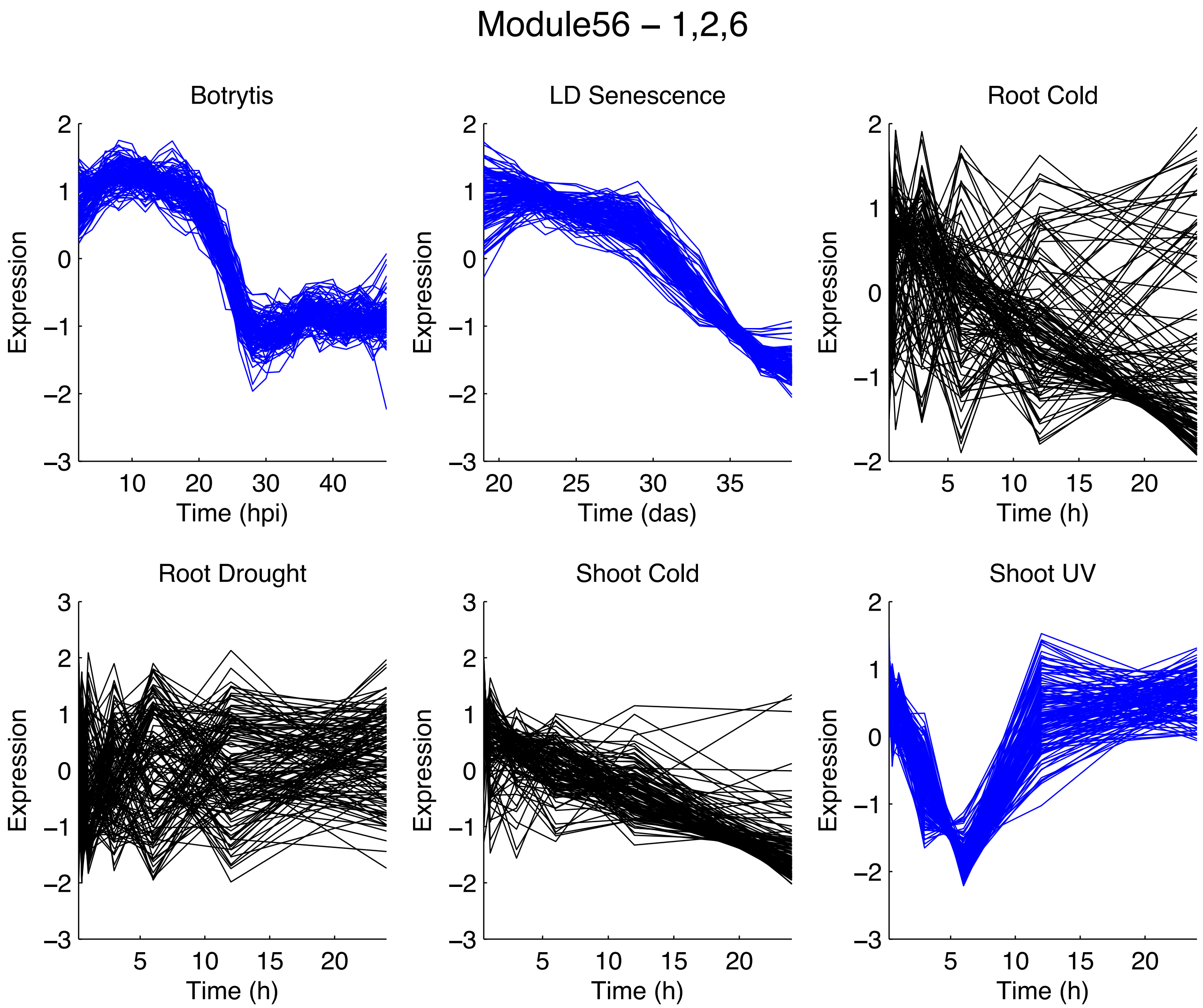
Module56 – 1,2,6
Botrytis
LD Senescence
Root Cold
Root Drought
Shoot Cold
Shoot UV
Expression
Time (hpi)
Time (das)
Time (h)

Module57 – 3,5

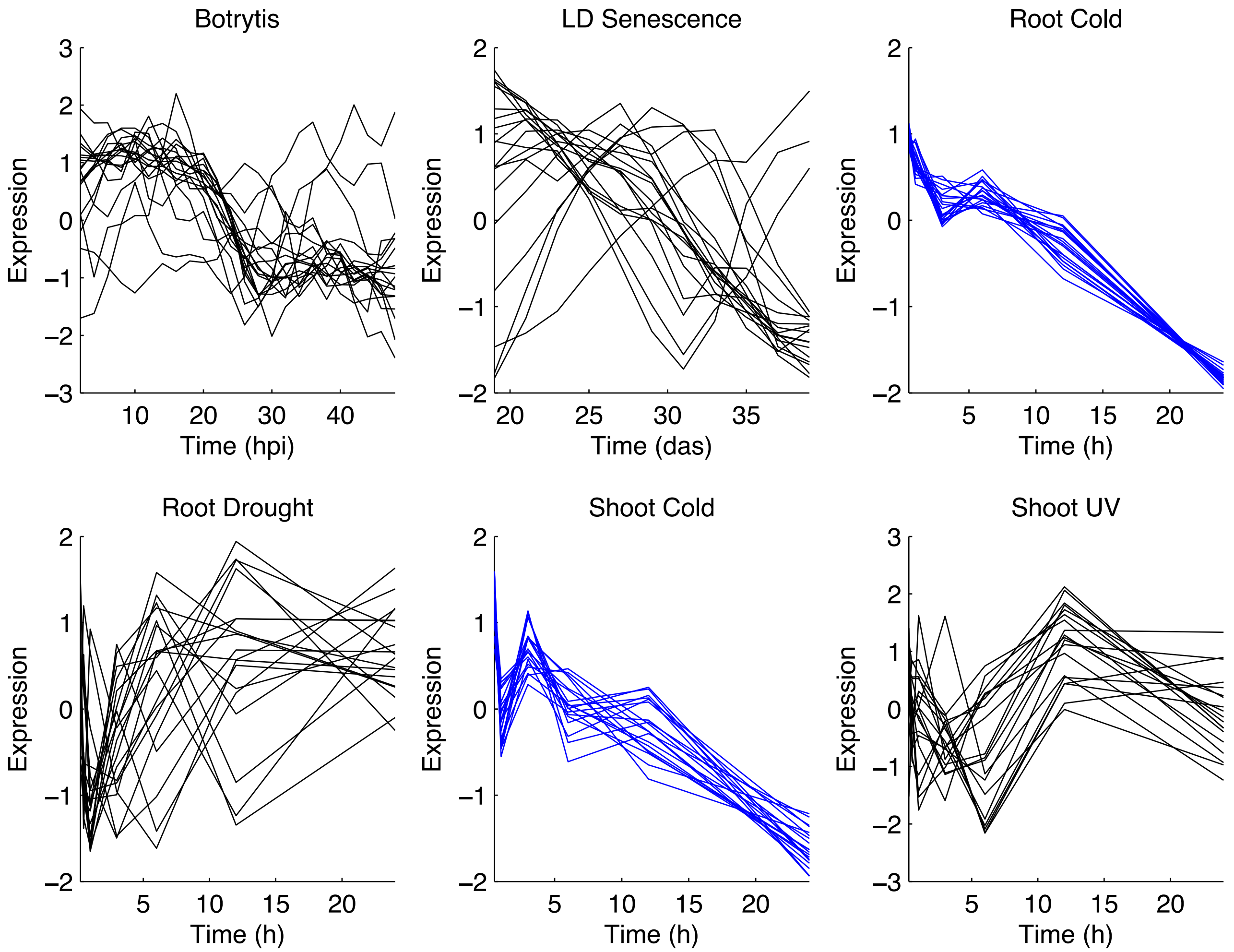

Botrytis
LD Senescence
Root Cold
Root Drought
Shoot Cold
Shoot UV
Expression
Time (hpi)
Time (das)
Time (h)

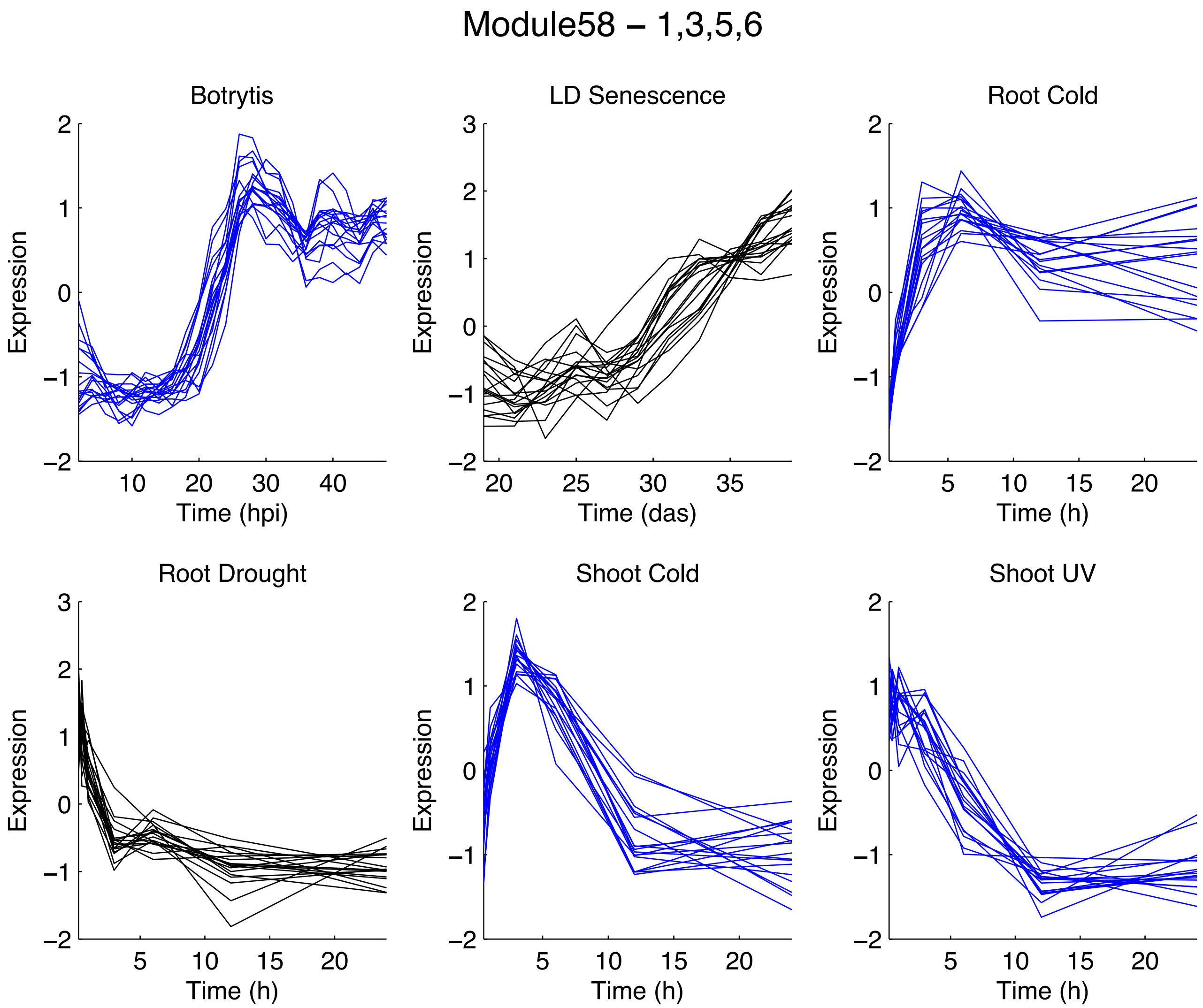
Module58 – 1,3,5,6
Botrytis
LD Senescence
Root Cold
Root Drought
Shoot Cold
Shoot UV
Expression
Time (hpi)
Time (das)
Time (h)

Module59 – 3,4,5

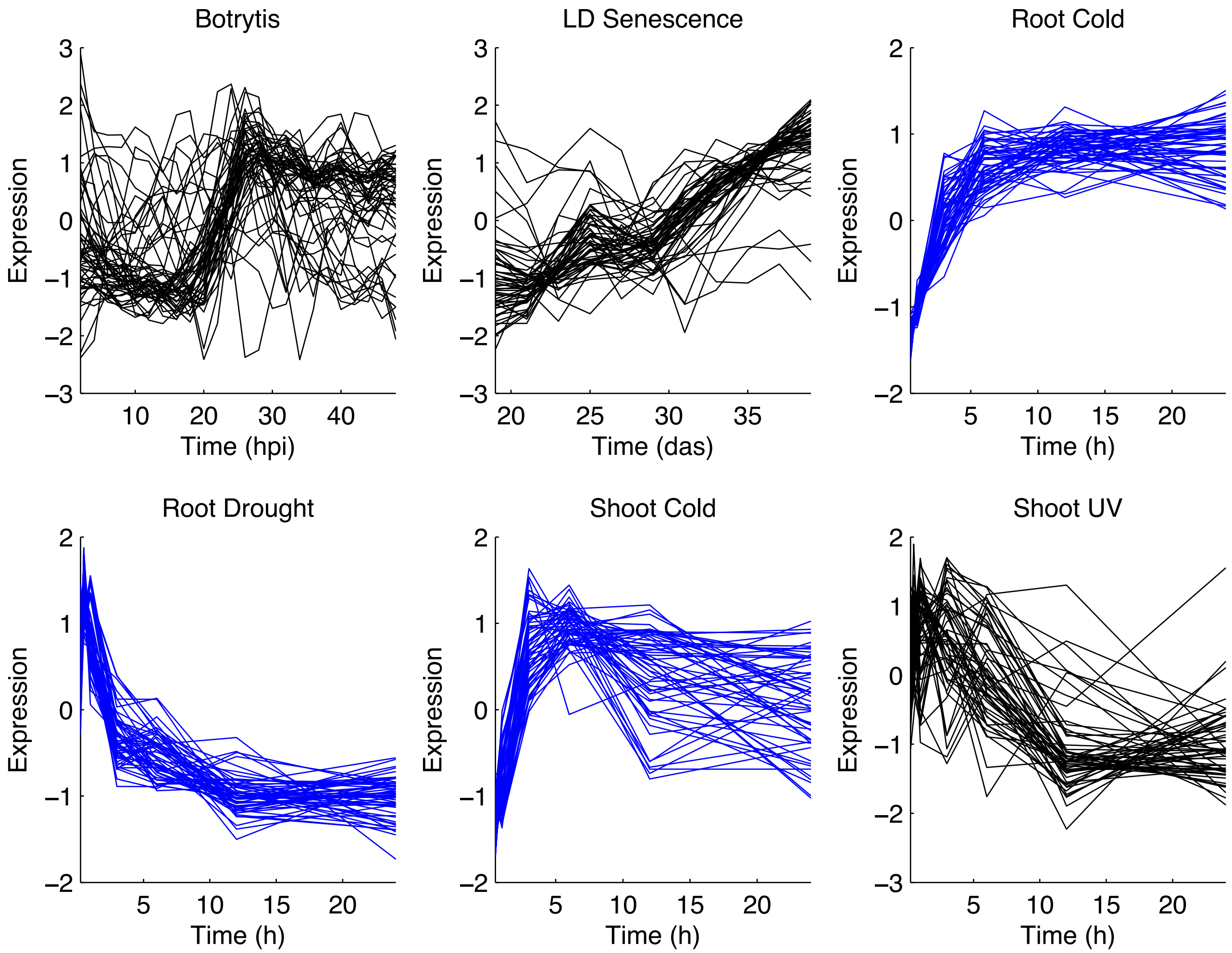

Module60 – 1,6

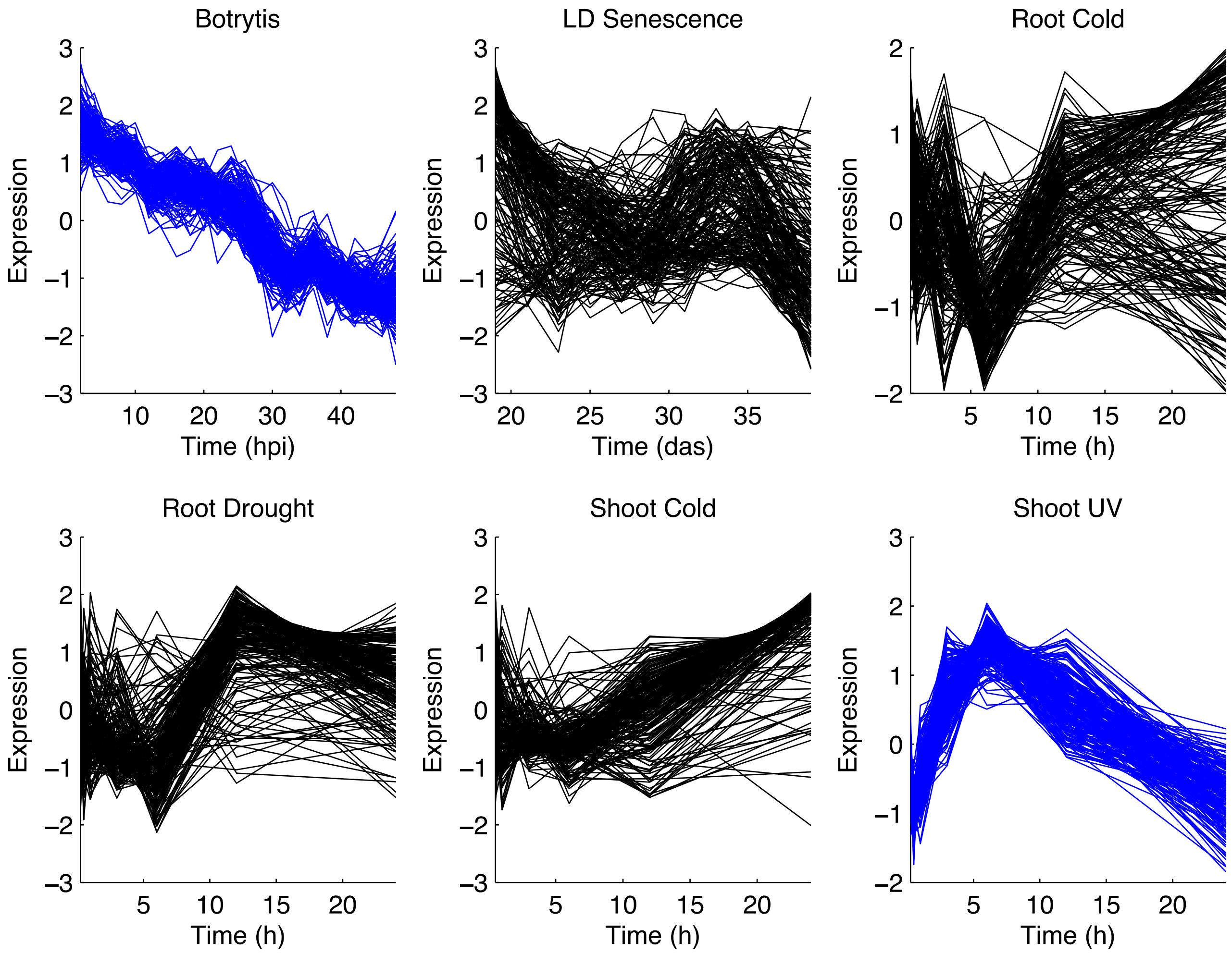

Botrytis
LD Senescence
Root Cold
Root Drought
Shoot Cold
Shoot UV
Expression
Time (hpi)
Time (das)
Time (h)

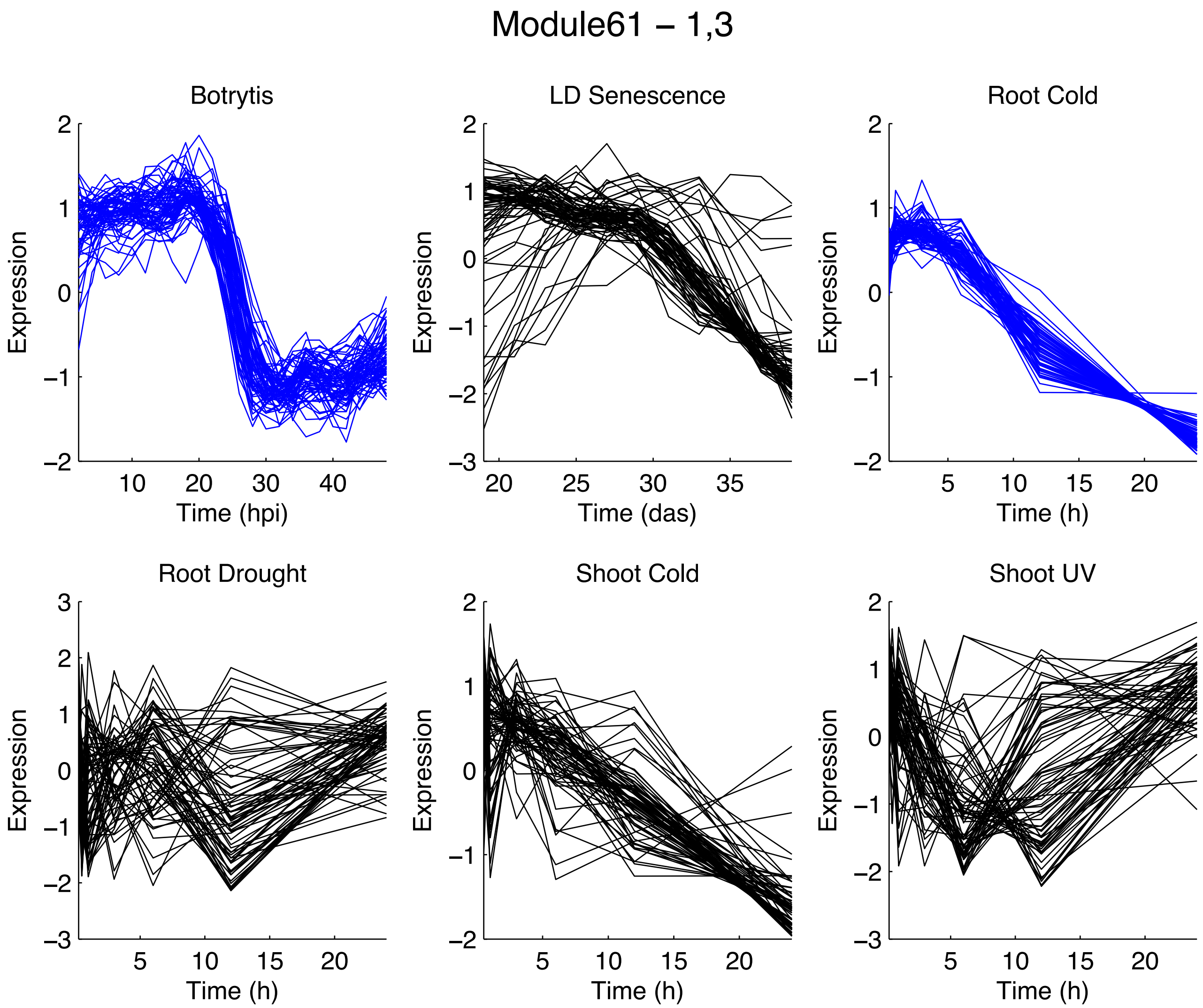
Module61 – 1,3
Botrytis
Expression
Time (hpi)
LD Senescence
Time (das)
Root Cold
Time (h)
Root Drought
Shoot Cold
Shoot UV

Module62 – 5,6

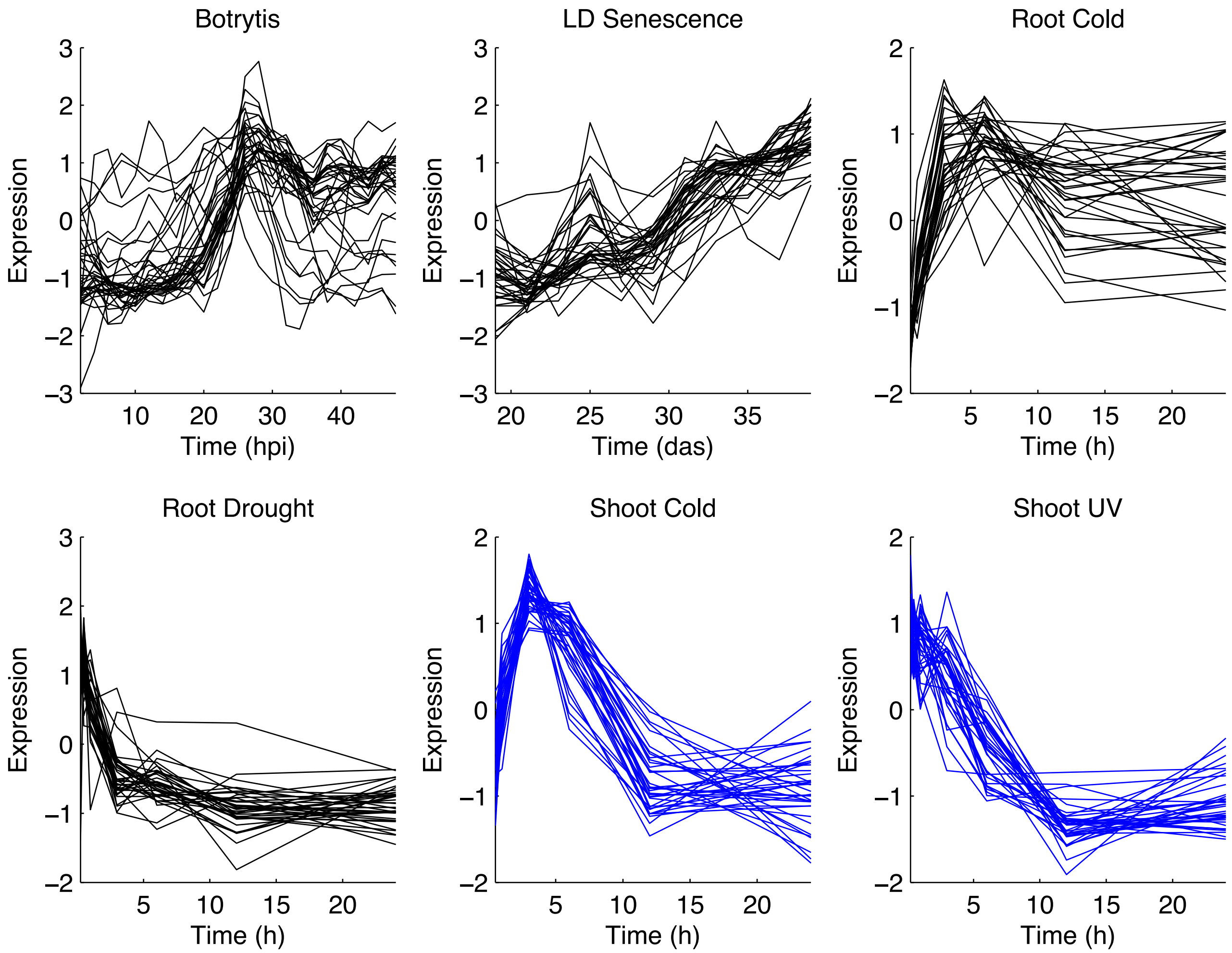
Botrytis
LD Senescence
Root Cold
Root Drought
Shoot Cold
Shoot UV
Expression
Time (hpi)
Time (das)
Time (h)
3
2
1
0
−1
−2
−3
10
20
30
40
25
35
5
15

Module63 – 1,5

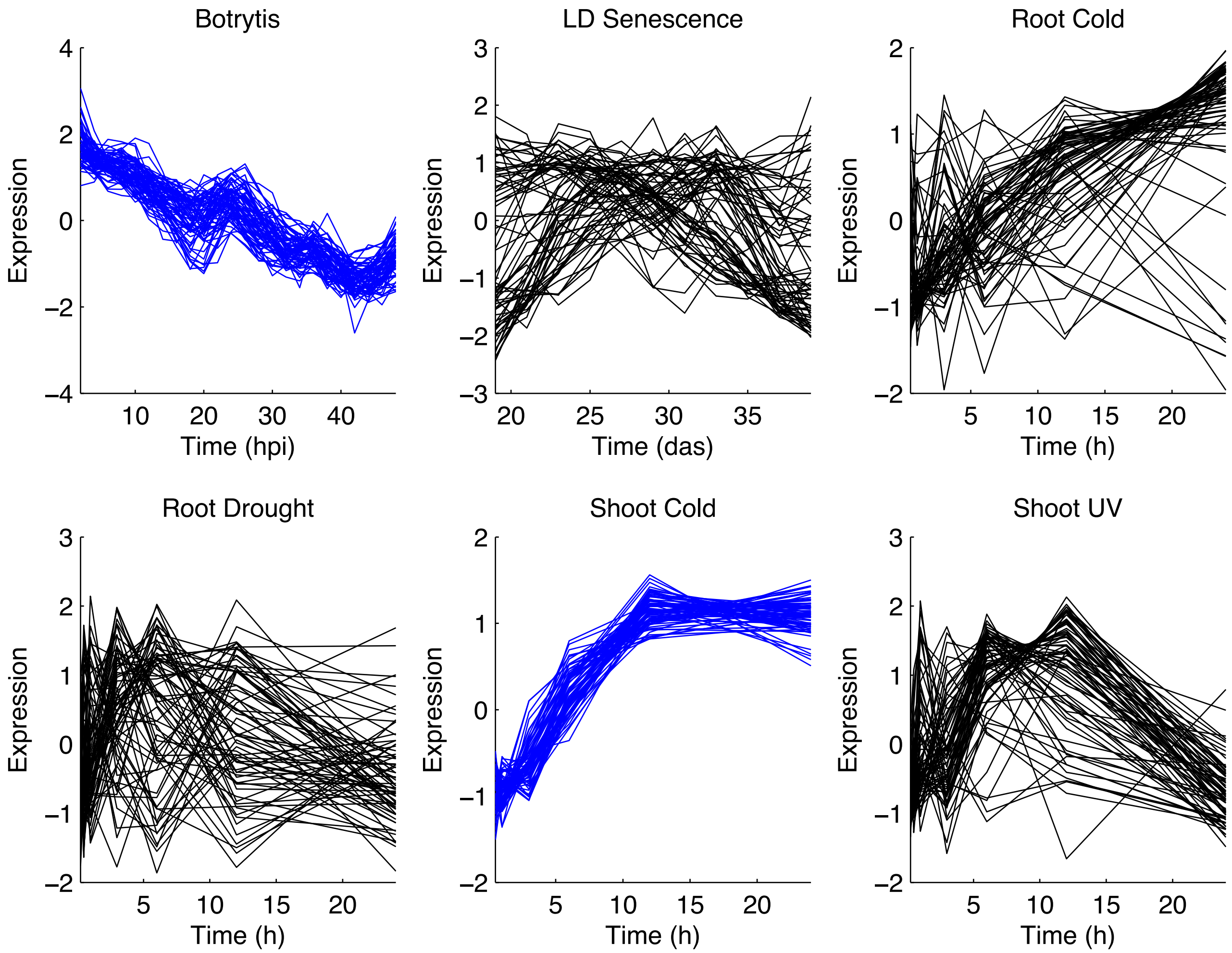

Botrytis
LD Senescence
Root Cold
Root Drought
Shoot Cold
Shoot UV
Expression
Time (hpi)
Time (das)
Time (h)

Module64 – 1,3

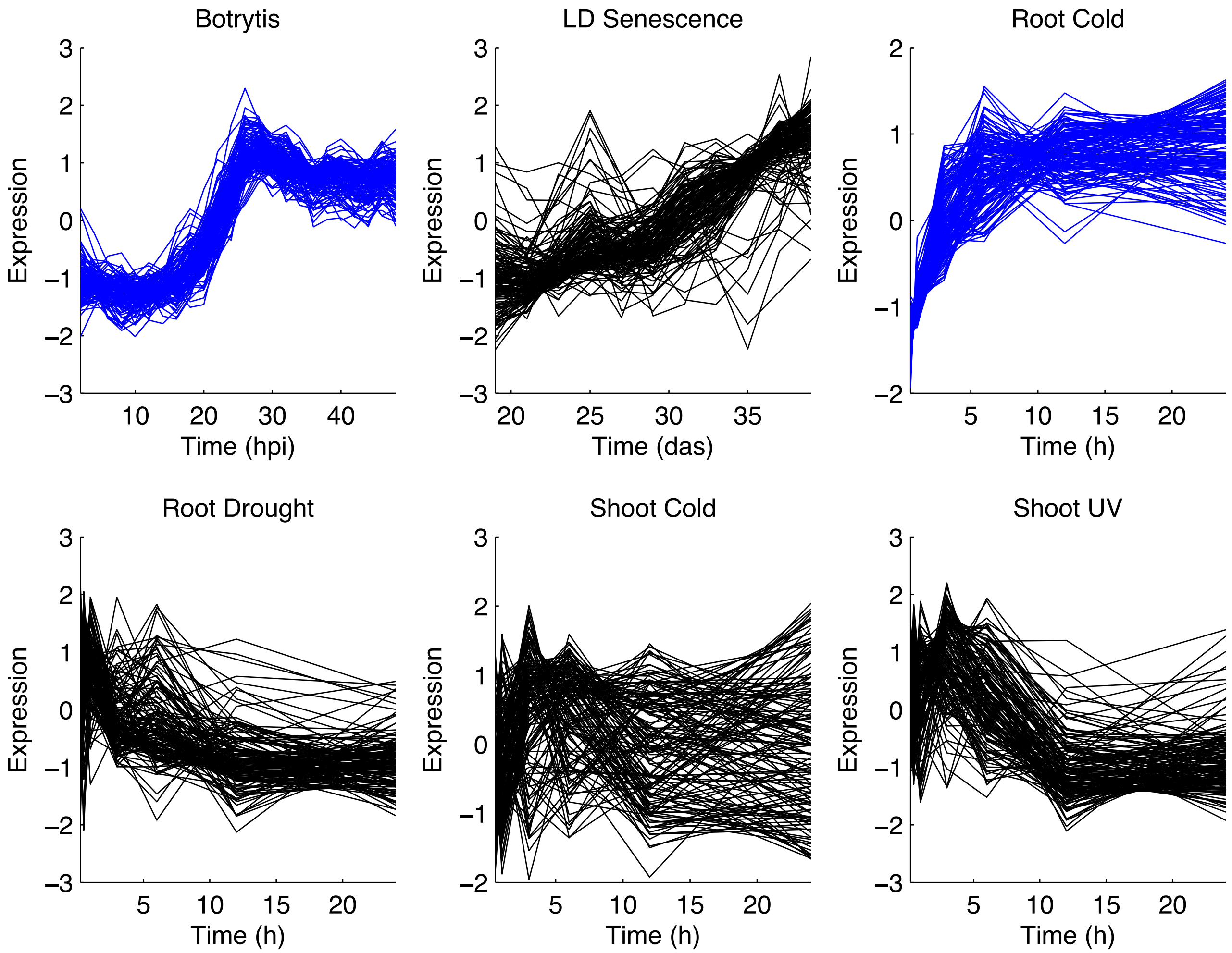

Botrytis
LD Senescence
Root Cold
Root Drought
Shoot Cold
Shoot UV
Expression
Time (hpi)
Time (das)
Time (h)

Module65 – 1,6

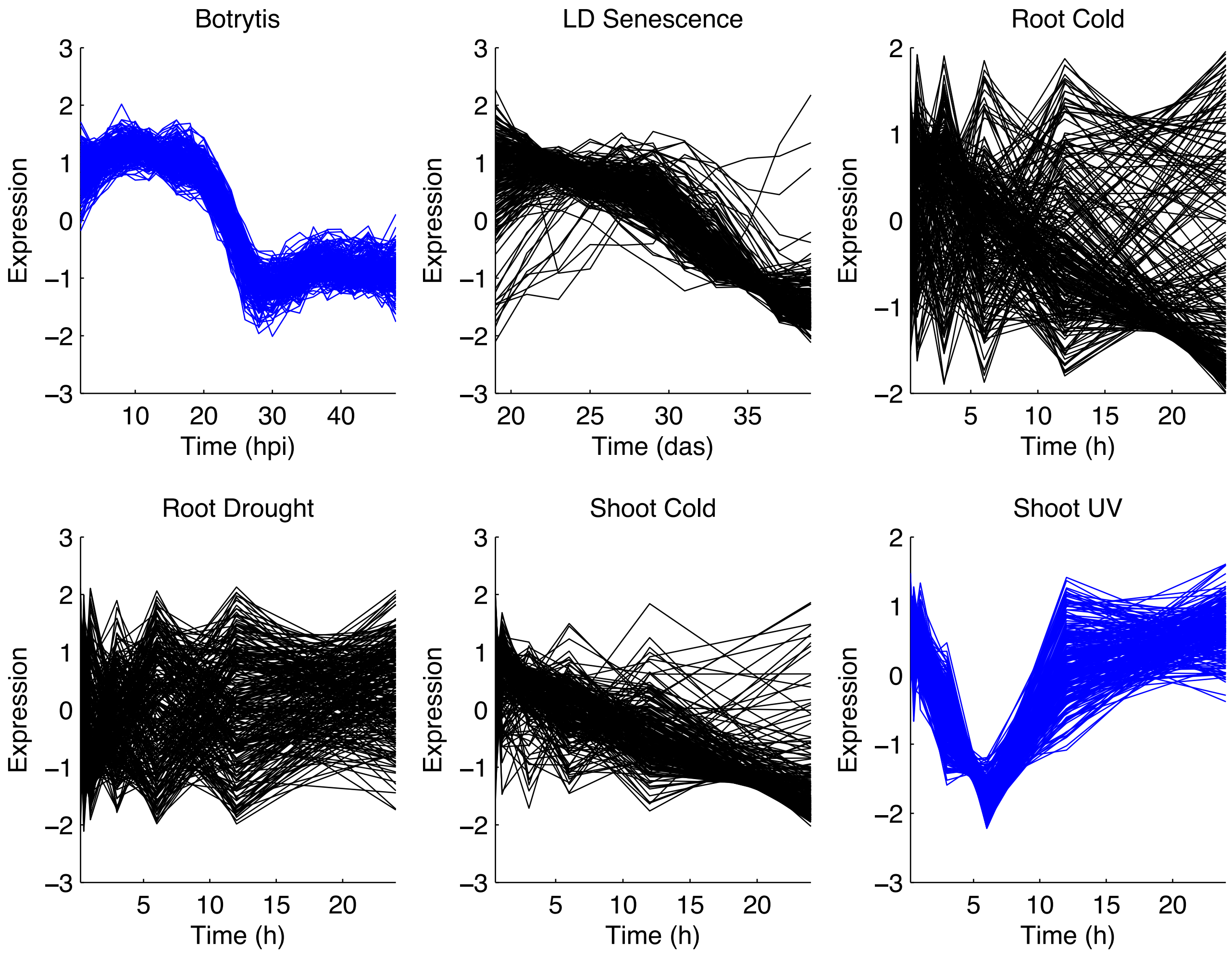
Botrytis
Expression
Time (hpi)
3
2
1
0
−1
−2
−3
10
20
30
40
LD Senescence
Expression
Time (das)
3
2
1
0
−1
−2
−3
20
25
30
35
Root Cold
Expression
Time (h)
2
1
0
−1
−2
5
10
15
20
Root Drought
Expression
Time (h)
3
2
1
0
−1
−2
−3
5
10
15
20
Shoot Cold
Expression
Time (h)
3
2
1
0
−1
−2
−3
5
10
15
20
Shoot UV
Expression
Time (h)
2
1
0
−1
−2
−3
5
10
15
20

Module66 – 1,4,5,6

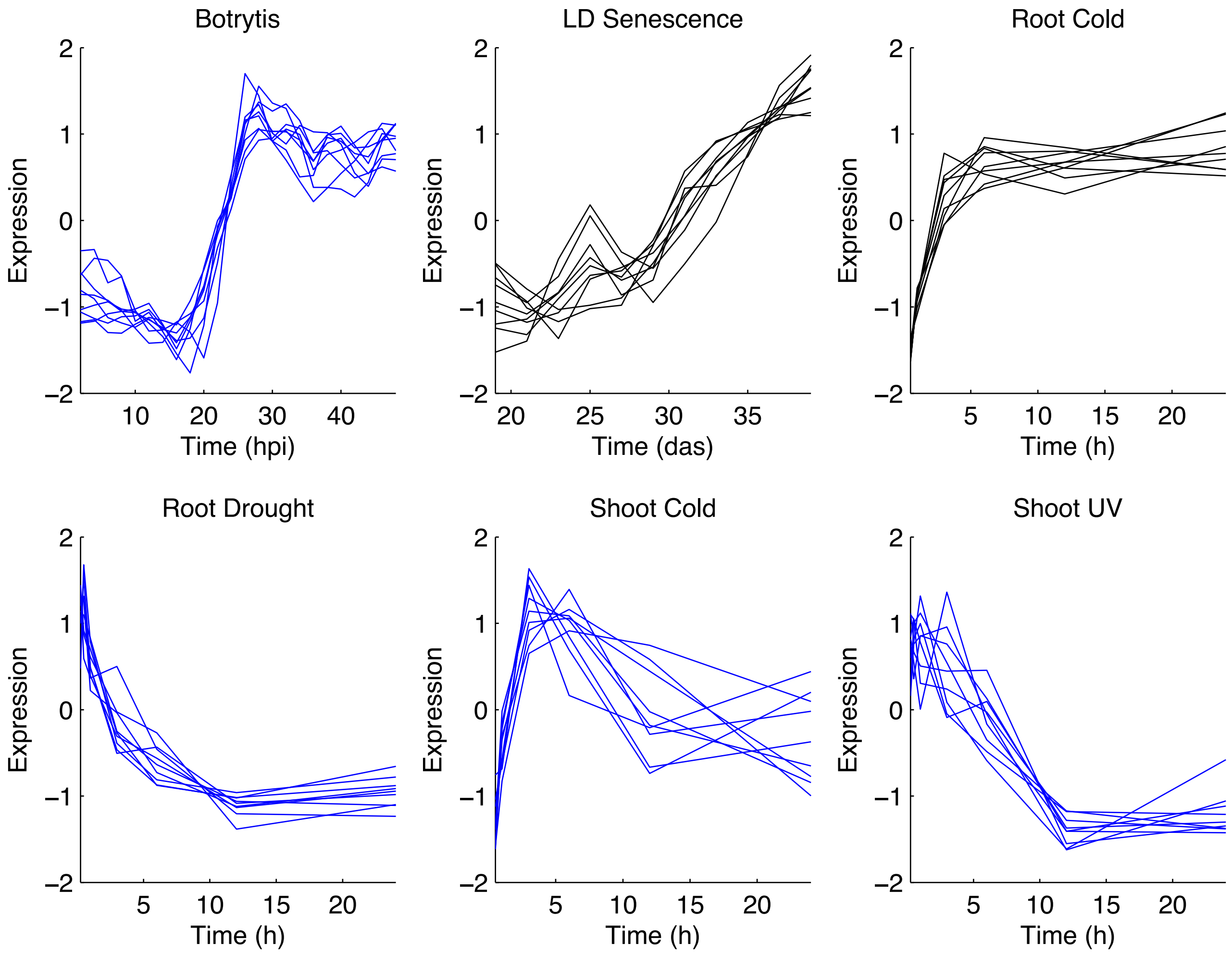

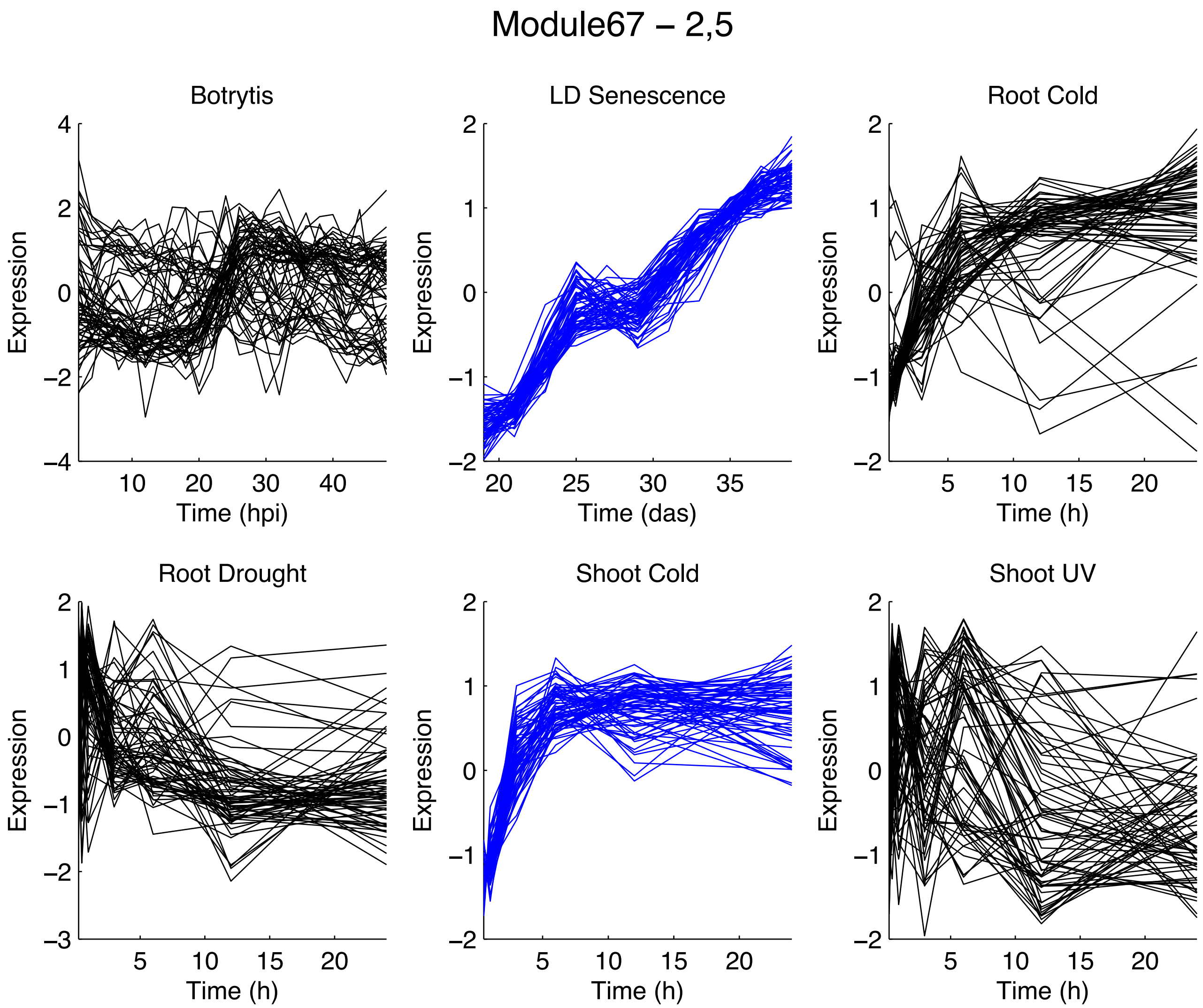
Module67 – 2,5
Botrytis
LD Senescence
Root Cold
Root Drought
Shoot Cold
Shoot UV
Expression
Time (hpi)
Time (das)
Time (h)

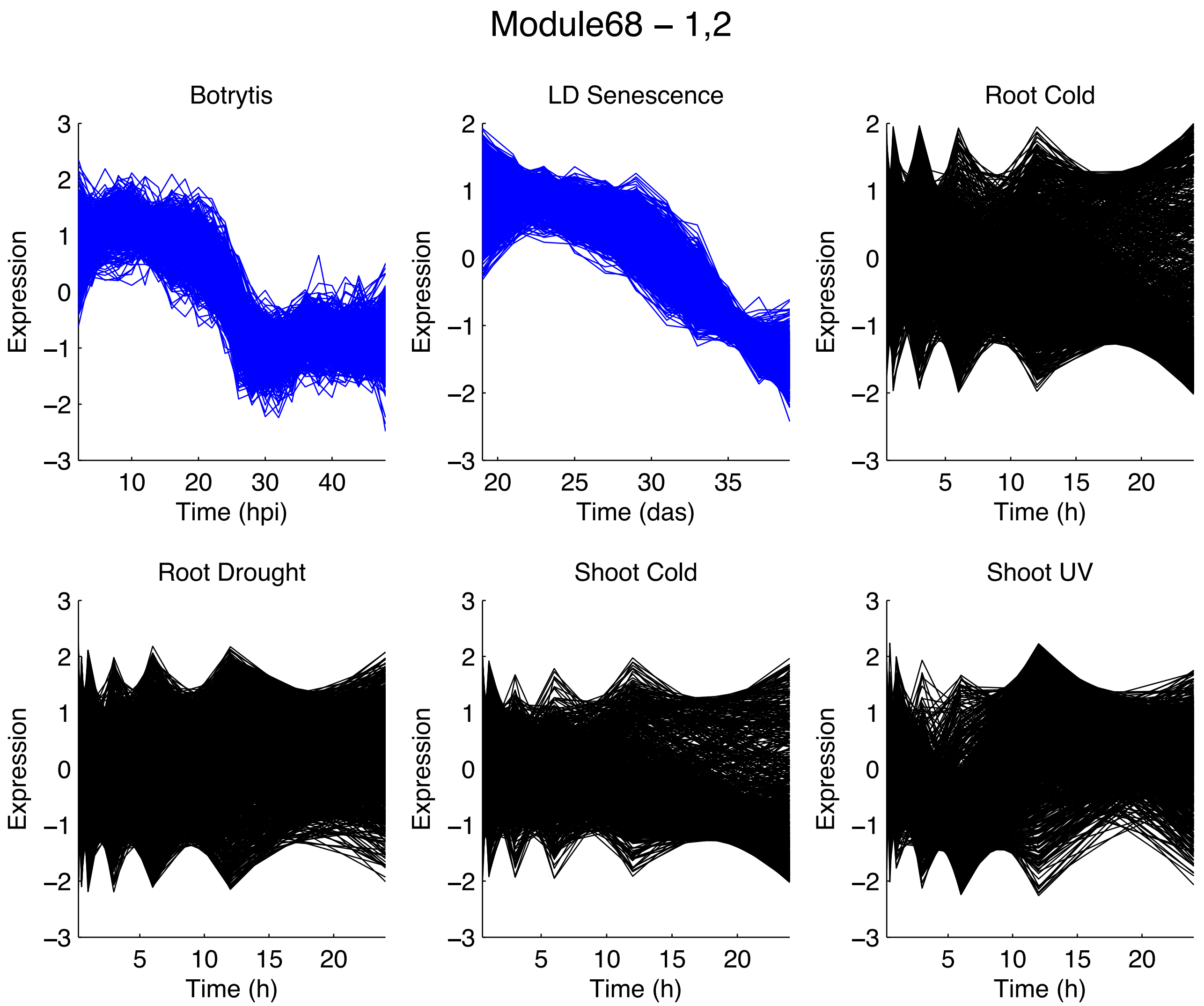
Module68 – 1,2
Botrytis
LD Senescence
Root Cold
Root Drought
Shoot Cold
Shoot UV
Expression
Time (hpi)
Time (das)
Time (h)

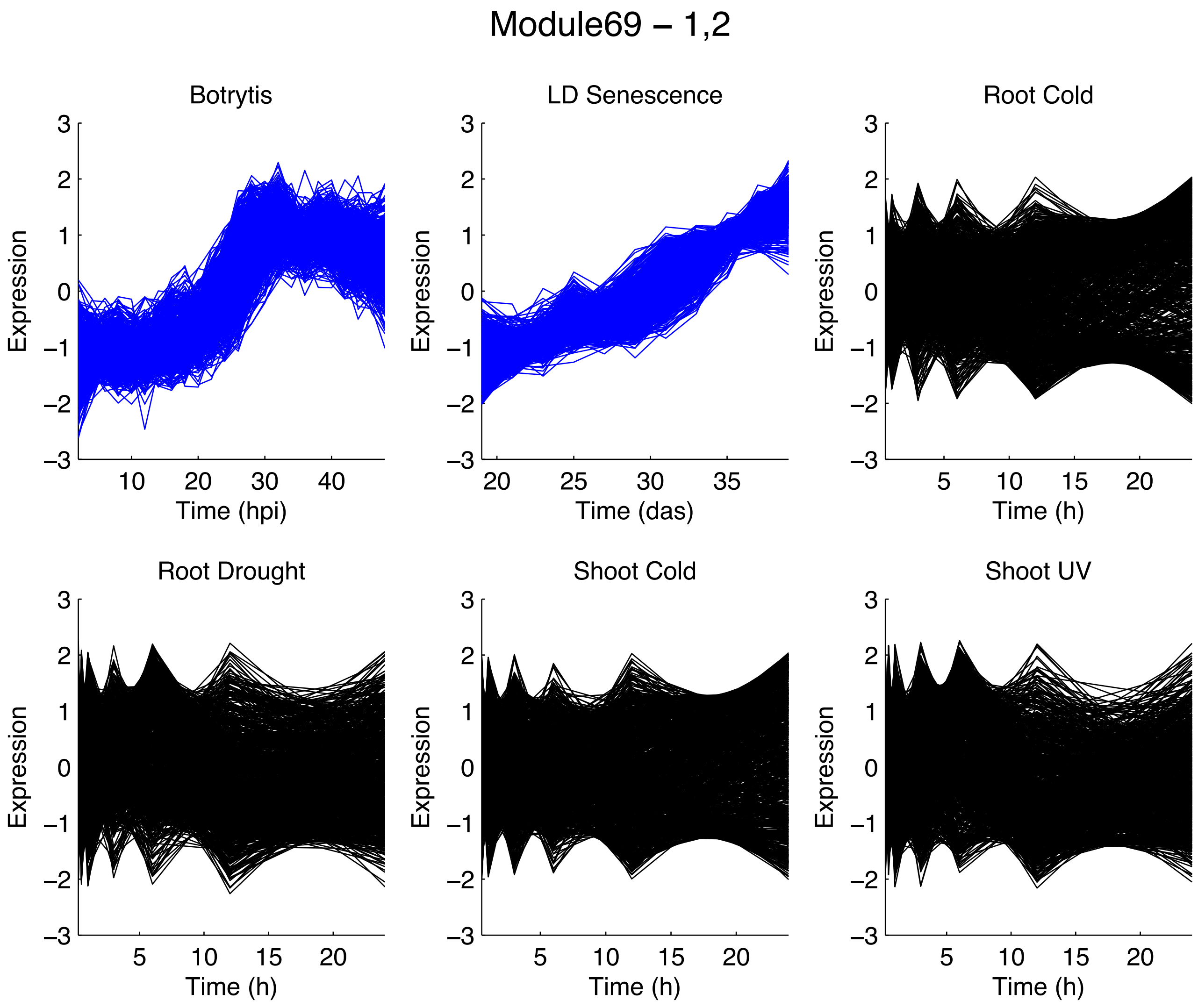
Module69 – 1,2
Botrytis
LD Senescence
Root Cold
Root Drought
Shoot Cold
Shoot UV
Expression
Time (hpi)
Time (das)
Time (h)

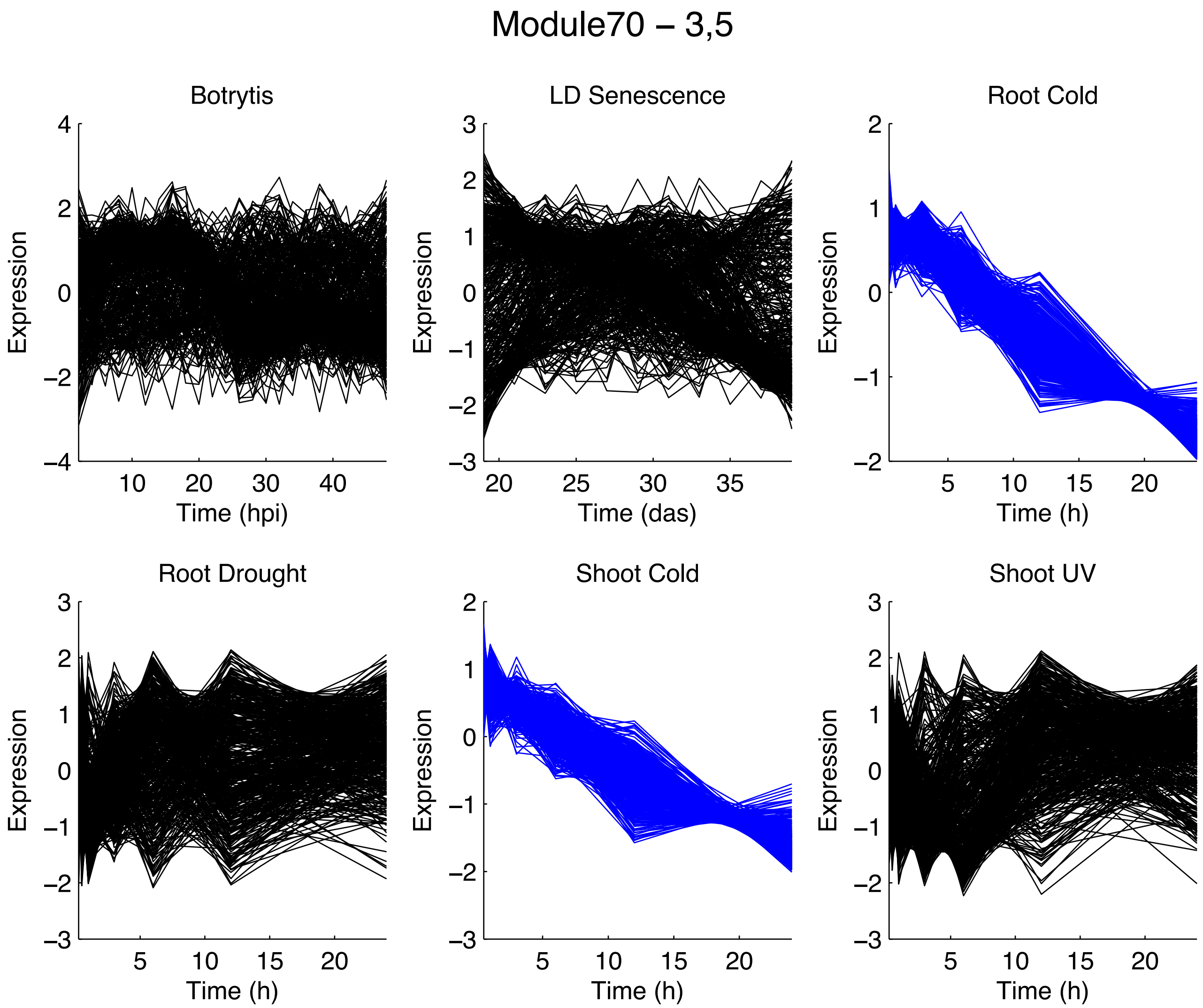
Module70 – 3,5
Botrytis
LD Senescence
Root Cold
Root Drought
Shoot Cold
Shoot UV
Expression
Time (hpi)
Time (das)
Time (h)

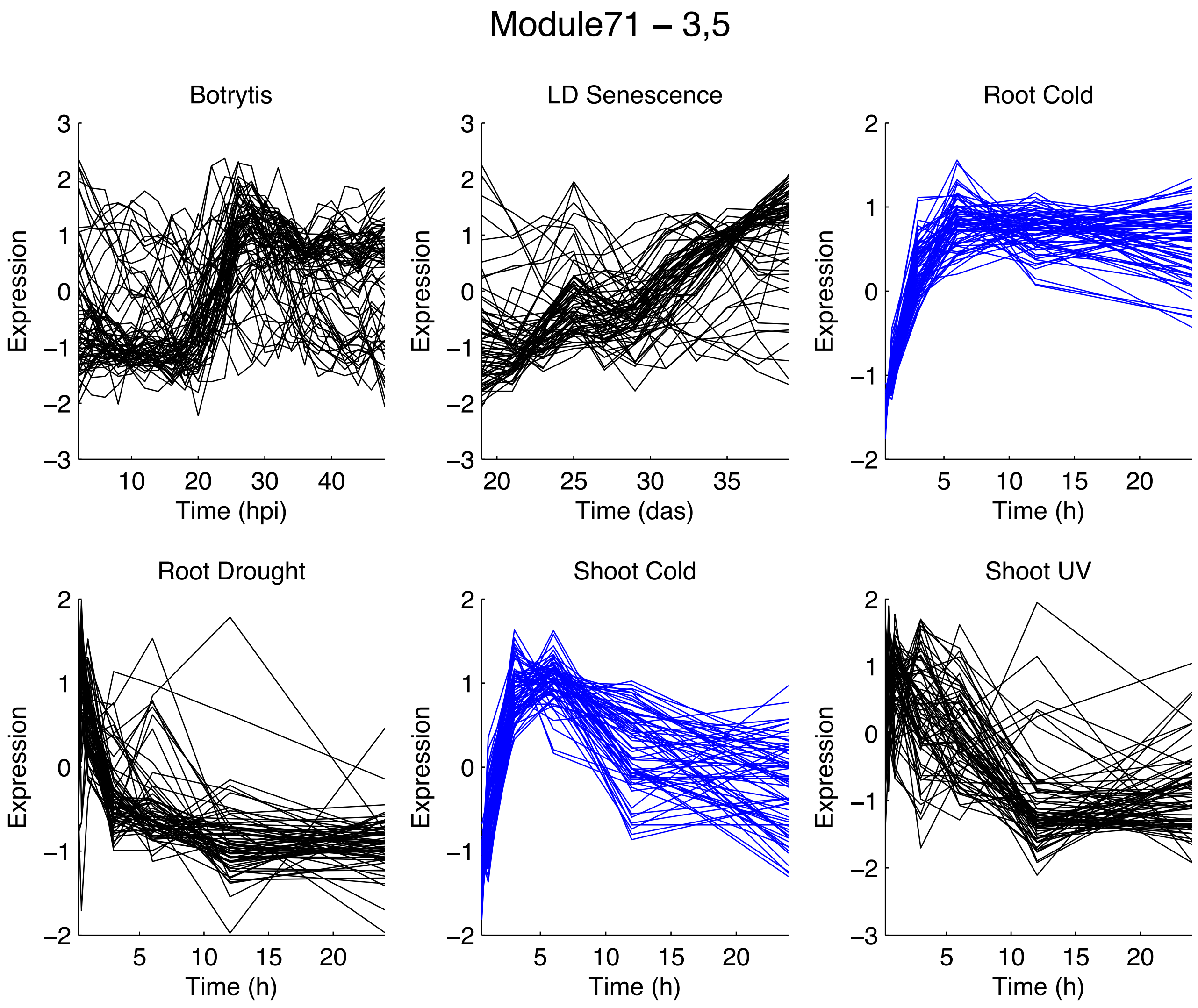
Module71 – 3,5
Botrytis
LD Senescence
Root Cold
Root Drought
Shoot Cold
Shoot UV
Expression
Time (hpi)
Time (das)
Time (h)

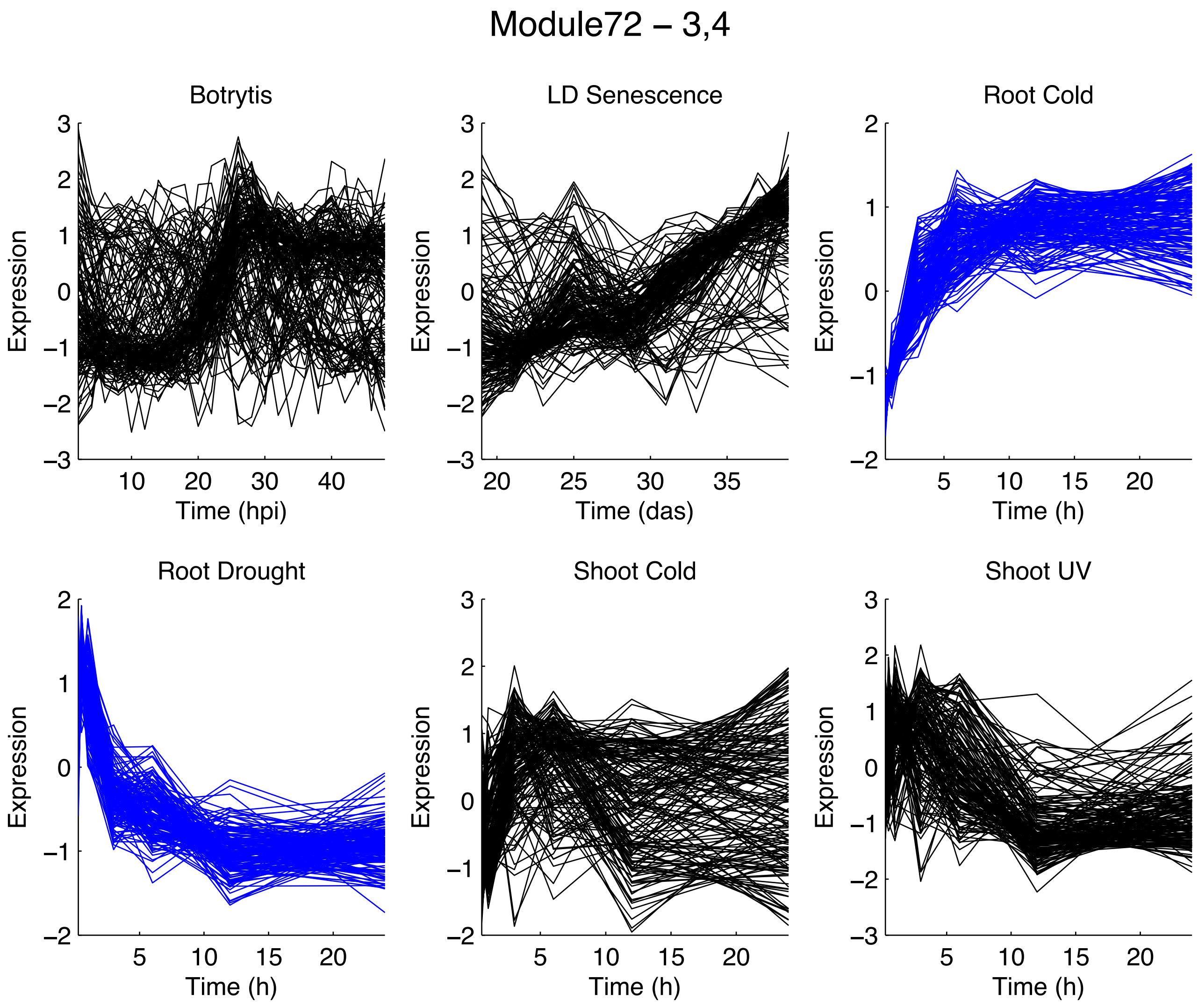

Module72 – 3,4
Botrytis
LD Senescence
Root Cold
Root Drought
Shoot Cold
Shoot UV
Expression
Time (hpi)
Time (das)
Time (h)

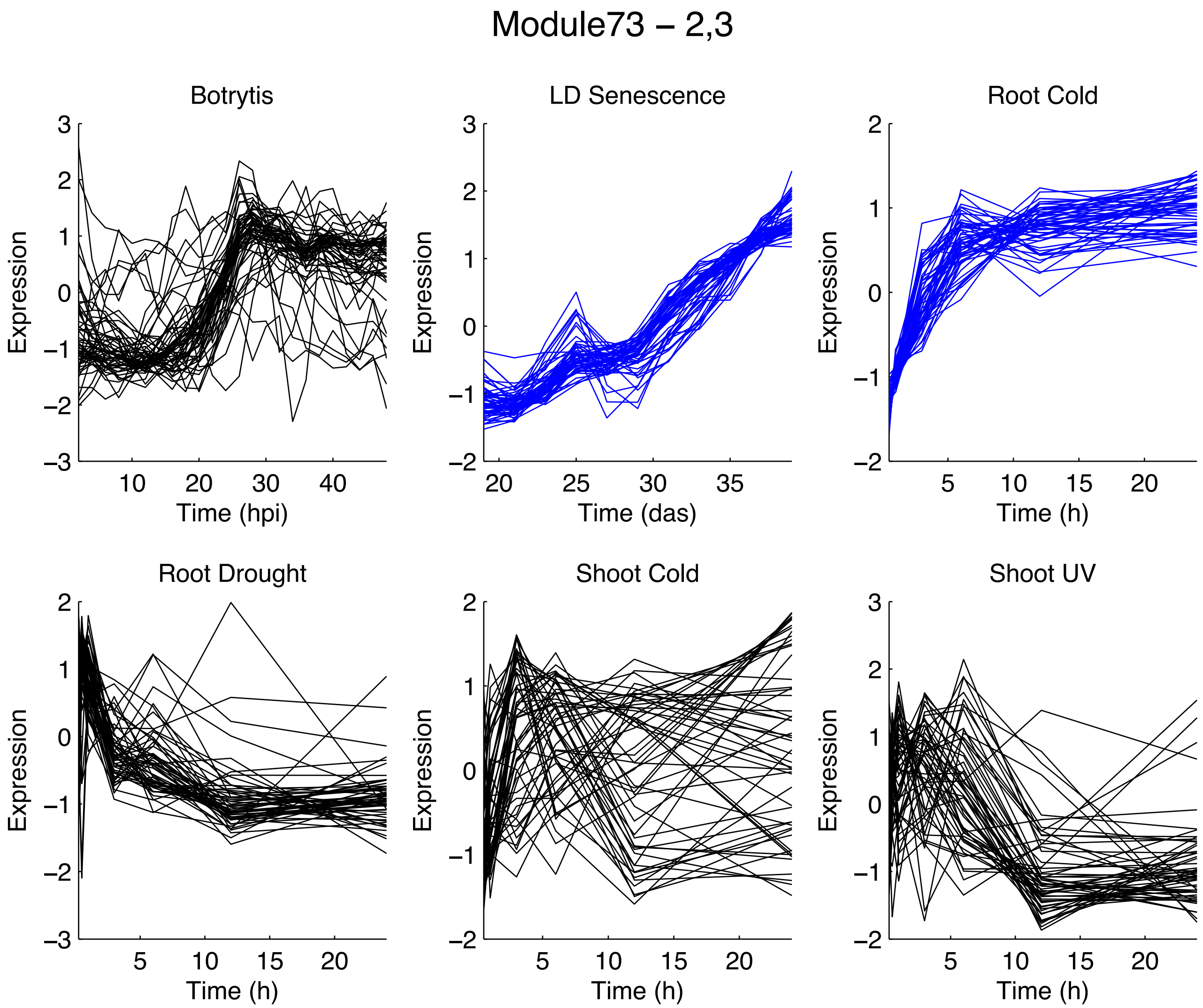

Module73 – 2,3
Botrytis
LD Senescence
Root Cold
Root Drought
Shoot Cold
Shoot UV
Expression
Time (hpi)
Time (das)
Time (h)

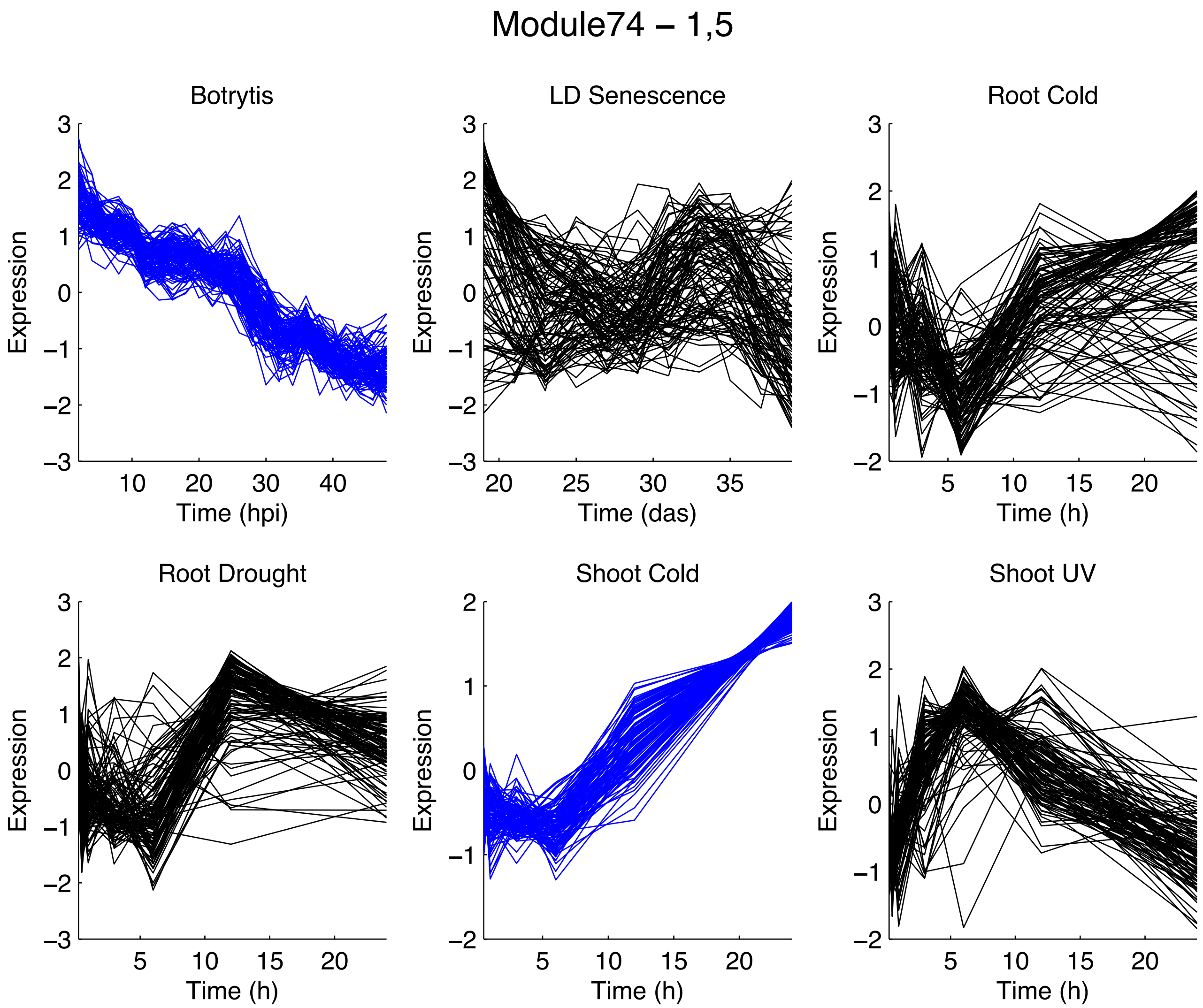
Module74 – 1,5
Botrytis
LD Senescence
Root Cold
Root Drought
Shoot Cold
Shoot UV
Expression
Time (hpi)
Time (das)
Time (h)

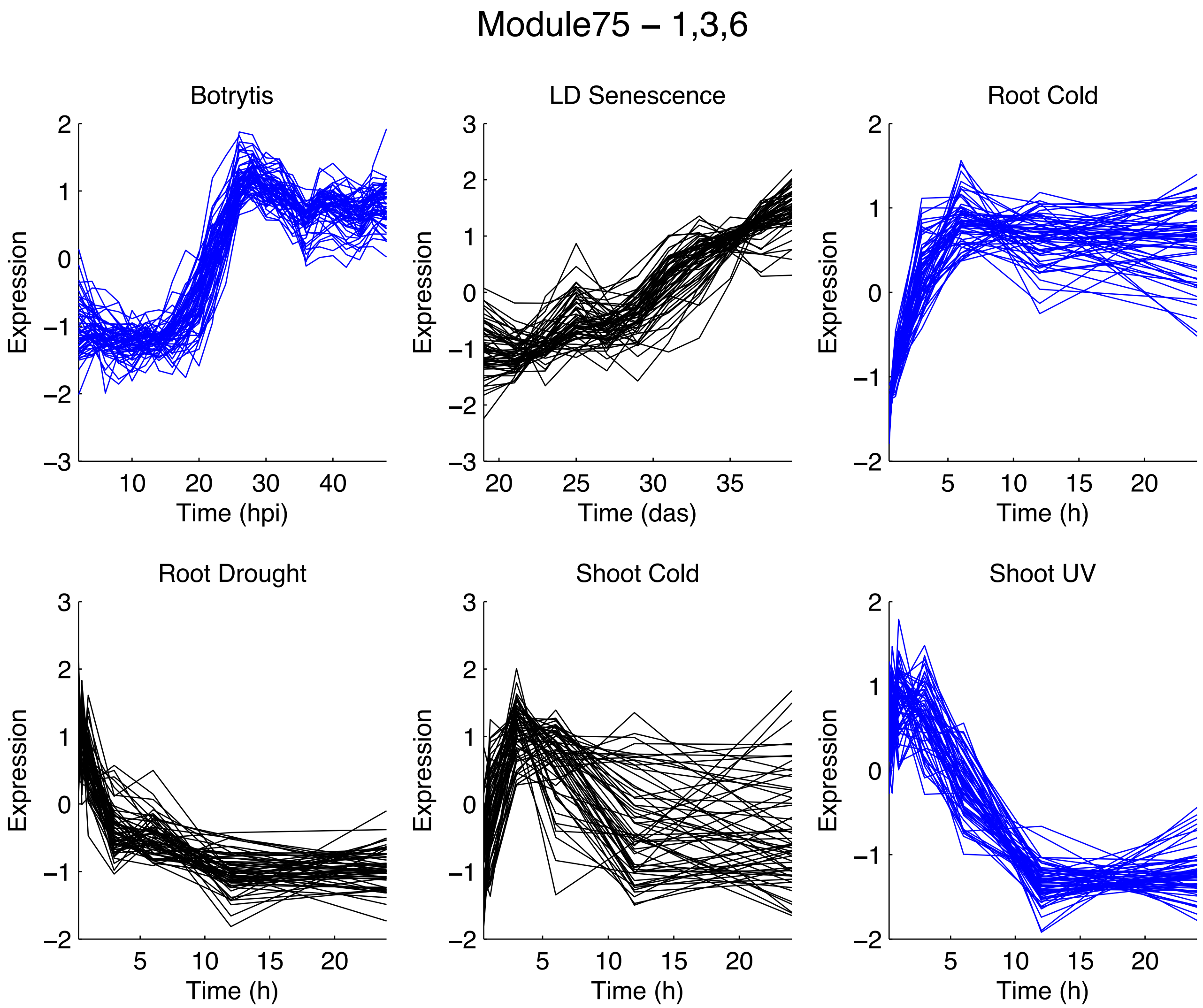
Module75 – 1,3,6
Botrytis
LD Senescence
Root Cold
Root Drought
Shoot Cold
Shoot UV
Expression
Time (hpi)
Time (das)
Time (h)

Module76 – 2,6

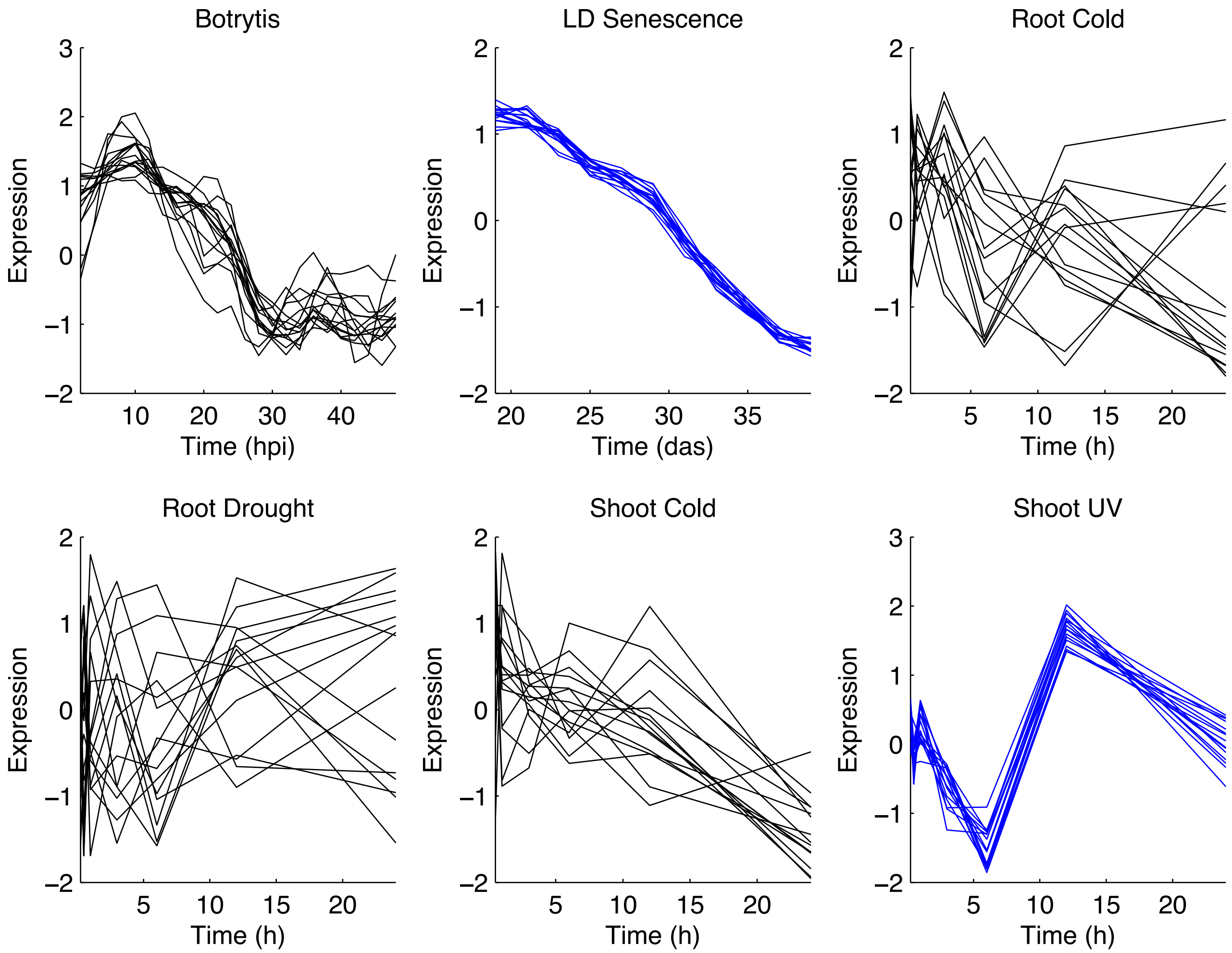
Botrytis
LD Senescence
Root Cold
Root Drought
Shoot Cold
Shoot UV
Expression
Time (hpi)
Time (das)
Time (h)

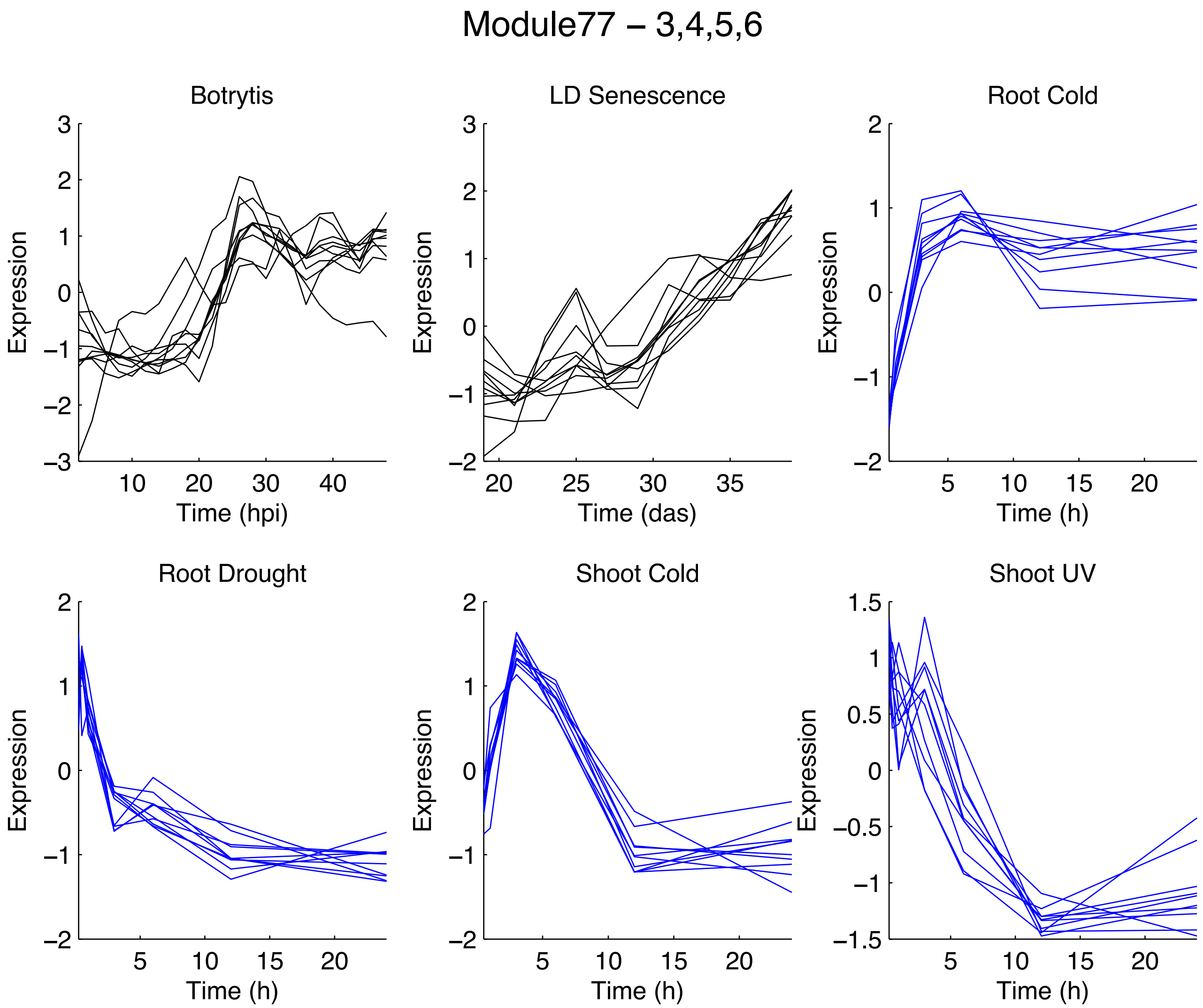
Module77 – 3,4,5,6
Botrytis
LD Senescence
Root Cold
Root Drought
Shoot Cold
Shoot UV
Expression
Time (hpi)
Time (das)
Time (h)

Module78 – 5,6

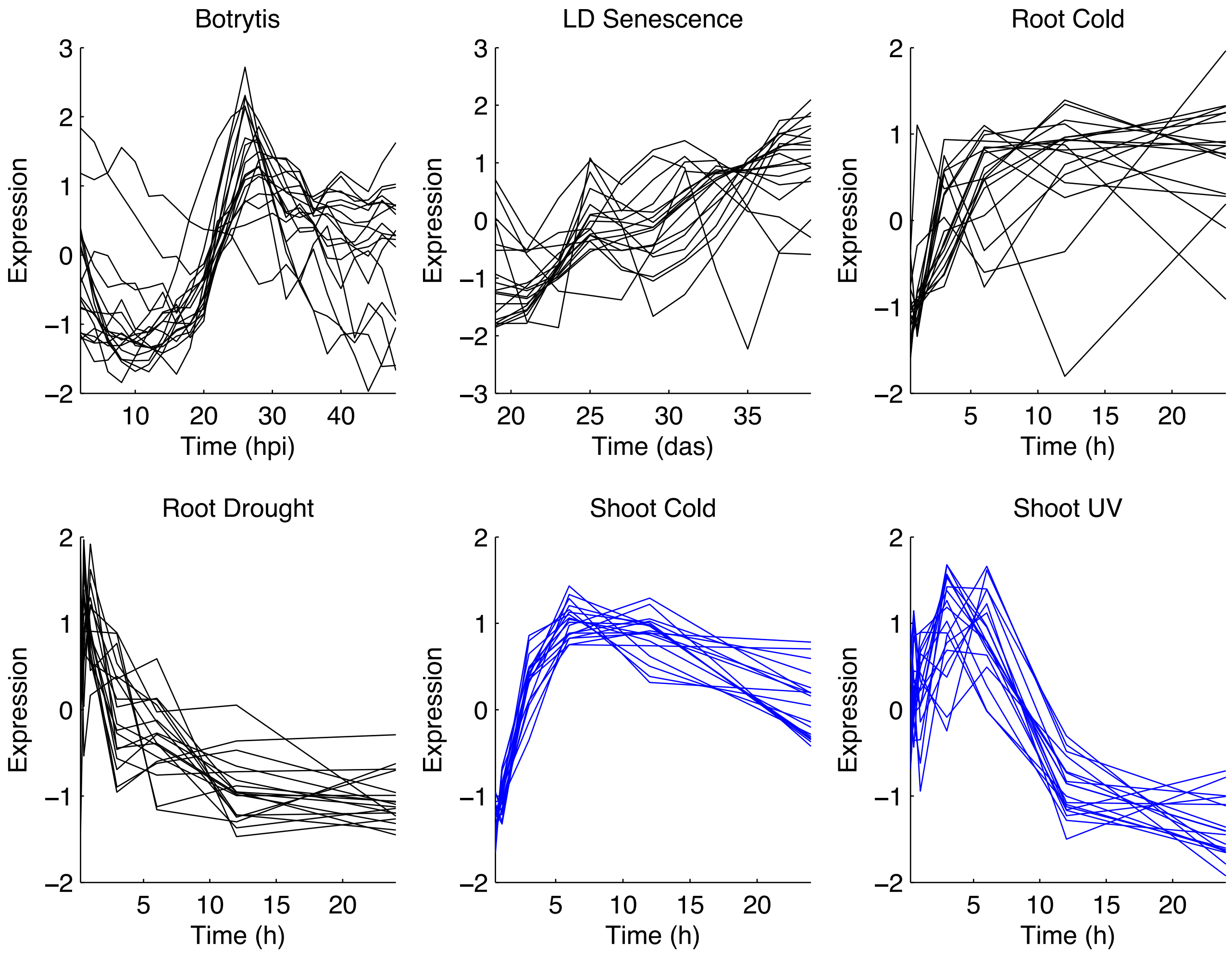
Botrytis
LD Senescence
Root Cold
Root Drought
Shoot Cold
Shoot UV
Expression
Time (hpi)
Time (das)
Time (h)
3
2
1
0
−1
−2
−3
10
20
30
40
25
35
5
15
